# Nineteenth century French rose (*Rosa* sp.) germplasm shows a shift over time from a European to an Asian genetic background

**DOI:** 10.1093/jxb/erw269

**Published:** 2016-07-12

**Authors:** Mathilde Liorzou, Alix Pernet, Shubin Li, Annie Chastellier, Tatiana Thouroude, Gilles Michel, Valéry Malécot, Sylvain Gaillard, Céline Briée, Fabrice Foucher, Cristiana Oghina-Pavie, Jérémy Clotault, Agnès Grapin

**Affiliations:** ^1^IRHS, Agrocampus Ouest, INRA, Université d’Angers, SFR 4207 QuaSaV, 49071, Beaucouzé, France; ^2^Flower Research Institute, Yunnan Academy of Agricultural Sciences, Kunming 650205, China; ^3^Université d’Angers, UMR CNRS 6258 CERHIO, Centre de recherches historiques de l’Ouest, 5 bis Bd Lavoisier 49045 Angers, France

**Keywords:** Diversity, genetic structure, historical resources, hybridization, ornamental plant, ploidy level, *Rosa* sp., SSR markers.

## Abstract

Hybridization with introduced genetic resources is commonly practiced in ornamental plant breeding to introgress desired traits. The 19th century was a golden age for rose breeding in France. The objective here was to study the evolution of rose genetic diversity over this period, which included the introduction of Asian genotypes into Europe. A large sample of 1228 garden roses encompassing the conserved diversity cultivated during the 18th and 19th centuries was genotyped with 32 microsatellite primer pairs. Its genetic diversity and structure were clarified. Wide diversity structured in 16 genetic groups was observed. Genetic differentiation was detected between ancient European and Asian accessions, and a temporal shift from a European to an Asian genetic background was observed in cultivated European hybrids during the 19th century. Frequent crosses with Asian roses throughout the 19th century and/or selection for Asiatic traits may have induced this shift. In addition, the consistency of the results with respect to a horticultural classification is discussed. Some horticultural groups, defined according to phenotype and/or knowledge of their pedigree, seem to be genetically more consistent than others, highlighting the difficulty of classifying cultivated plants. Therefore, the horticultural classification is probably more appropriate for commercial purposes rather than genetic relatedness, especially to define preservation and breeding strategies.

## Highlight

The impact of breeding on the genetic diversity and structure of roses, during the 19th century in Europe, was studied using a genetic and historical interdisciplinary approach.

## Introduction

Breeding plants has been and still is a matter of combining new interesting traits with traits already present. This is particularly true with ornamental plants which are prone to fashion trends. Ornamental breeding is often geared towards creating diversity and generating novelty more than improving agronomic traits. In France, in the early 19th century, innovation in roses was obtained either via seeds derived from random intermating of selected garden varieties or by vegetative propagation of sports (Oghina-Pavie, 2015). In the 1830s and 1840s, artificial crossing was adapted to roses, with a progressive shift towards controlled hybridization (Oghina-Pavie, 2015).

With >24 000 varieties available (listed in Roberts *et al.*, 2003), roses are among the most sold and popular ornamental plants. Their cultivation is ancient, known during Roman antiquity (Pliny, 77; Touw, 1982) and even 5000 years ago in China (Wang, 2005). Wild species are mostly found in Asia and Europe, but some are native to North America and North Africa (Gudin, 2000). Roses are of various ploidy levels, ranging from 2*x* to 10*x*, with a monoploid number of *x*=7 chromosomes (Hurst, 1925, 1927; Yokoya *et al.*, 2000; Jian *et al.*, 2010).

Genetic relationships are complex in the *Rosa* genus due to the high number of species (100–250 according to various authors) of various ploidy levels, their worldwide distribution, and the high level of interspecific hybridization. Consequently, classifying cultivated and botanical roses has always been controversial. The botanical classification of wild roses published by Rehder (1940), and updated by Wissemann (2003), divides the genus *Rosa* into four subgenera: *Hesperodos*, *Hulthemia*, *Platyrhodon*, and *Rosa*, with the latter being divided into 11 sections. It is often said that even though they have broad phenotypic diversity, cultivated rose ancestors derived from 7–10 species from the sections *Synstylae* (*R. moschata*, *R. wichurana*, and *R. multiflora*), *Rosa* (*R. gallica*), *Indicae* (*R. chinensis* and *R. gigantea*), and *Pimpinellifoliae* (*R. foetida*). *Rosa spinosissima* in section *Pimpinellifoliae*, and *R. cinnamomea* and *R. rugosa* in section *Cinnamomeae* made a small but noticeable contribution to the current diversity (Martin *et al.*, 2001; Adumitresei and Stănescu, 2009; Smulders *et al.*, 2011). Cultivated roses are usually horticulturally classified based on their phenotype. The American Rose Society (ARS) horticultural classification scheme (2000) divides *Rosa* accessions into three groups: (i) species also referred to as wild or botanical roses (wild roses cultivated in botanical gardens); (ii) old garden roses that existed prior to 1867, the year of the creation of the first Hybrid Tea ‘La France’, containing 21 subdivisions; and (iii) modern roses containing 13 subdivisions (Roberts *et al.*, 2003). It is considered as a reference, but is still a work in progress (Cairns, 2003).

Before the 18th century, two major domestication areas existed, one in Europe and the other in Asia (Maia and Venard, 1976; Smulders *et al.*, 2011). In Europe until the 19th century, roses were hardy, cold resistant, and flowered only once (occasional repeat-flowering roses can be found, e.g. ‘Quatre saisons’ according to Hurst, 1941). The color range was limited to red, pink, white, and probably pale yellow. Roses were diploid (*R. moschata*), tetraploid (*R. gallica*, *R. damascena*, and *R. centifolia*), or hexaploid (*R. alba*). Simultaneously, in Asia, another pool of mainly diploid roses (*R. semperflorens* and *R. indica*) was bred. They had interesting traits, such as continuous flowering and tea perfume. In the early 19th century in France, there was growing interest in roses for ornamental purposes, as indicated by the high number of rose ‘hybridizers’. At this time, Asian roses were introduced into Europe and progressively used in breeding. This hybridization resulted in the emergence of the Hybrid Tea roses from which most modern roses derived. The introduction of Asian roses may have had an impact on the genetic diversity of roses during the 18th to 19th centuries (Wylie, 1954; Iwata *et al.*, 2000; Joyaux, 2003; Marriott, 2003).

In previous studies, molecular markers have been used to characterize the genetic diversity of specific groups of roses representing one region (Wu *et al.*, 2000; De Cock, 2008; Samiei *et al.*, 2010) or one classification subdivision (see, for example, Martin *et al.*, 2001; Gardes *et al.*, 2005; Babaei *et al.*, 2007; Vukosavljev *et al.*, 2013). The samples ranged from 15 (Iwata *et al.*, 2000) to 218 (Gardes *et al.*, 2005) accessions with a noteworthy sample of 2161 roses (De Cock, 2008). Scariot *et al.* (2006) studied the genetic relationships of 65 old garden roses. The present study was aimed at obtaining a broader view of the evolution of the diversity of roses bred in France during the 19th century by assessing a larger sample of roses from this period. Indeed, thanks to vegetative propagation and the passion of some people (private and public rose gardens), century-old garden roses were preserved, making them a good material to study the impact of breeding, as cultivars of different periods are still alive. The goal was to test the following hypotheses: (i) genetic differentiation between ancient European and Asian accessions; (ii) genetic differentiation in European accessions bred before and after the time when Asian accessions were widely introduced into European germplasm; and (iii) genetic consistency in the horticultural classification.

## Materials and methods

### Plant material

A total of 1459 rosebushes were harvested between 2007 and 2013 from 10 rose gardens in France (addresses listed in Supplementary Table S1 at *JXB* online). After genotyping—since analyses may be sensitive to missing data—1228 genotypes (Supplementary Table S2, data available at Zenodo DOI 10.5281/zenodo.56704) having <30% missing data were kept for most of the analyses. No particular category (botanical or cultivated, geographic origin, ploidy, breeding year, and horticultural group) was affected by the deleted genotypes. The 1228 accessions included 940 European garden roses from the 18th and 19th centuries (631 roses from France) including seven roses considered as old garden roses but with an unknown breeding year. This set represented the existing conserved diversity of French roses from this period. Other roses were used as a comparison: 15 roses were widely used in Europe before the 18th century, 46 were roses that would have been bred in China (including 23 roses with an unknown breeding year), 56 were contemporary roses from the 20th to 21st centuries (including five roses from Asia), and 118 were botanical roses including 33 roses from Europe and 54 from Asia.

The contemporary roses chosen were among the most widely known and sold (http://www.worldrose.org/awards/hof/hof.asp). The botanical roses were either cultivated individuals from wild species (individuals or offspring of an individual sampled in its natural environment that had not been bred) or an accession coming from a wild species but presenting a remarkable trait and therefore called a cultivar.

**Fig. 1.**
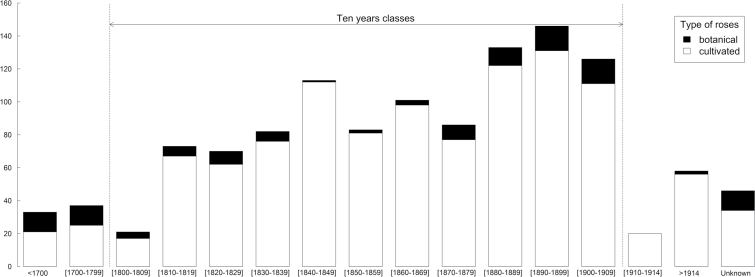
Breeding year distribution for the 1110 bred roses and years of introduction for the 118 botanical roses in 15 periods.

Several informative variables were collected on each accession: breeding year, horticultural group (according to the ARS classification), geographic origin, and ploidy level. To enhance the accuracy, the breeding year information was collected by historians from different historical sources (Supplementary Notes S1). Otherwise, breeding dates in surveyed rose garden databases, in the web database HelpMeFind (http://www.helpmefind.com/rose), or in the book ‘Modern roses 12’ (Young *et al.*, 2007), were kept. The years 1700 and 1914 were chosen as limits for the 18th to 19th century period, as the beginning of the First World War was more a break point in France for the 19th century breeding activities than 1900 (referred to as the ‘long 19th century’). The accessions were then distributed into 15 temporal classes: before 1700; 1700–1799; 11 classes of 10 years from 1800 to 1909; 1910–1914; and after 1914 (Fig. 1).

**Fig. 2.**
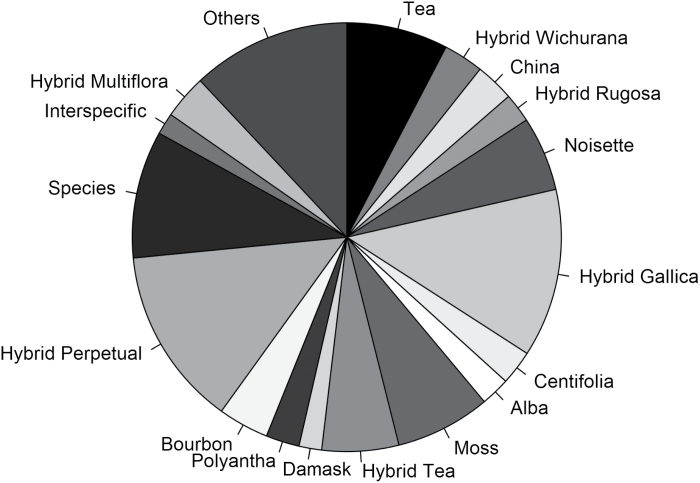
Proportion of assignation to the horticultural classification of the 1228 roses.

The same sources were used for the horticultural classification and geographic origin. In the case of differences between the classification data sources, the book ‘Modern roses 12’ (Young *et al.*, 2007), using the ARS classification, was used as reference. The main represented horticultural groups were Hybrid Perpetual, Hybrid Gallica, Tea, Moss, and Hybrid Tea (Fig. 2).

Classification according to the ARS, published in ‘Modern roses 12’ (Young *et al.*, 2007) was used as reference.

**Fig. 3.**
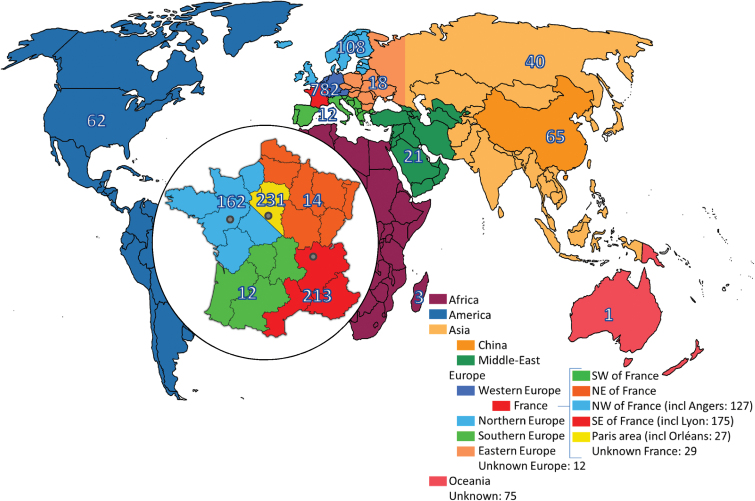
Areas of origin of the 1228 sampled roses. The number of roses from each region is displayed. In the circle, a focus is made on France.

Concerning geographical data, France was divided into eight areas based on the location of rose breeders: four large areas (northeast, northwest, southeast, and southwest) and four limited areas around cities where rose breeding was more popular during this period (Angers, Paris, Lyon, and Orléans). The rest of Europe was divided into four regions: western, eastern, northern, and southern Europe. Finally, the rest of the world was divided into five areas, according to the continents: Africa (corresponding to Réunion, former Bourbon Island, where Bourbon roses appeared in the 19th century), America, Asia, the Middle-East, and Oceania (Fig. 3).

Ploidy was measured on young fresh leaves by flow cytometry for a subsample of 353 genotypes representative of the diversity. A 1cm^2^ piece of the material was chopped up with the same quantity of pea leaf (*Pisum sativum*) tissue, used as an internal reference standard, in a Petri dish with 500 µl of cell lysis buffer. After this step, 1.5ml of this buffer mixed with DAPI was added. This suspension was filtered through a 30 µm nylon mesh and the fluorescence was analyzed with a Partec PA II flow cytometer (Partec GmbH, Münster, Germany) equipped with a mercury arc lamp (HBO/100). Ploidy levels for other accessions were obtained from the literature (Roberts *et al.*, 2003; Joyaux, 2005; Nadeem, 2012; Vukosavljev *et al.*, 2013; http://data.kew.org/cvalues/; http://www.helpmefind.com/rose) or extrapolated from other accessions of the same horticultural groups. For example, *Gallicanae* accessions whose ploidy level was not directly assessed were assumed to be tetraploids as reported for all assessed accessions in this group (Wissemann, 2003).

### Genotyping

Healthy young leaves were harvested, deep frozen, and lyophilized. DNA was extracted from ~10mg of lyophilized leaves. Extractions were conducted using the Qiagen 96 Plant Kit (Qiagen, Hilden, Germany) or the NucleoSpin^®^ 96 Plant II Core Kit (Macherey-Nagel, Düren, Germany) according to the manufacturer’s instructions. To optimize the use of the Qiagen 96 Plant Kit, an incubation step at 65 °C for 30min was added after lysis, just before adding Buffer P3. DNA quality was checked on 1.2% agarose gels. DNA quantification was evaluated by the fluorescence intensity of extracts in agarose gel containing λ phage DNA standards of 5–100ng µl^–1^ (Promega, Fitchburg, WI, USA) or with DNA-specific fluorescent Hoechst 33258 using a microplate reader (FLUOstar Omega, BMG Labtech, Ortenberg, Germany). DNA was then normalized at 5ng µl^–1^ with the Zephyr^®^ Compact Liquid Handling Workstation (PerkinElmer, Woodbridge, ON, Canada).

All individuals were genotyped with 32 microsatellite primer pairs [simple sequence repeats (SSRs); Süss and Schultze, 2003; Yan *et al.*, 2005; Zhang *et al.*, 2006; Hibrand-Saint Oyant *et al.*, 2008; Meng *et al.*, 2009; Spiller *et al.*, 2011; Gar *et al.*, 2011] covering each linkage group with 3–6 markers on each (Supplementary Table S1). All primer pairs were amplified using 4-plex PCR with 1× Qiagen Multiplex PCR Master Mix, 1.25 µM of each primer, and 10ng of DNA in a final volume of 5 µl. The following program was implemented using a DNA Engine Tetrad 2 thermal cycler (Bio-Rad, Richmond, CA, USA): preliminary denaturation for 15min at 95 °C, followed by 30s at 95 °C, 1min 30s at 55 °C (ramping from 95 °C to 55 °C by 1 °C s^–1^), and 75s at 72 °C (ramping from 55 °C to 72 °C by 1 °C s^–1^) repeated for 35 cycles, and then a final elongation step for 15min at 60 °C and 15min at 72 °C. In each 96-well plate, four controls were added (‘Black Baccara’, ‘Old Blush’, *Rosa*×*wichurana*, and ‘The Fairy’) and one well served as a negative control (water). Amplification products were analyzed with an ABI 96-capillary 3730XL DNA Analyser (ABI Prism, Applied Biosystems, Foster City, CA, USA) at the Gentyane platform in Clermont-Ferrand (France). Allele scoring was performed by two different persons with GeneMapper 4.1 software (Applied Biosystems) and scores were compared using an R procedure developed by F. Vallée, F. Dupuis, and A. Pernet (personal communication). When the two scores differed, the best one was chosen in agreement with the two readers. For an individual, a total absence of peak for one primer pair was considered as missing data. As some peaks were not assigned to the right bin (noticeable according to their size), a technique analogous to that explained in Amos *et al.* (2007) was used: allele sizes were visually checked and, in some cases, reassigned to the right bin with closer allele sizes. Due to the difficulty in estimating the SSR allele dosage in polyploids, alleles were coded as presence/absence; that is, sometimes named ‘allele phenotypes’ (Becher *et al.*, 2000; Rodzen *et al.*, 2004; Roullier *et al.*, 2013). It is theoretically possible to determine the number of alleles using methods such as MAC-PR (Esselink *et al.*, 2004), but they may be biased and hard to apply successfully for all markers (Vanderpoorten *et al.*, 2011; Vukosavljev *et al.*, 2013), and some studies suggest that they are not reliable for individuals with high or unknown ploidy levels (Scariot *et al.*, 2006; Helsen *et al.*, 2009; Sampson and Byrne, 2012).

### Diversity analysis

To assess the diversity, the number of observed alleles (*Ao*), the mean number of alleles per individual (*Am*), the effective number of alleles (*Ae*), and the number of rare alleles were calculated for each SSR. The following formula was used to calculate *Am* for SSR α: *Am* α$=\frac{\sum^{\text{​}}n_{k\alpha }}{N_{\alpha }},$ where *n*_*k*α_ is the number of alleles carried by individual *k* for SSR α and *N*_α_ is the number of genotyped individuals for SSR α. *Ae* for SSR α was then calculated according to the formula from Hamrick and Godt (1990): *Ae* α$=\frac{1}{\sum^{\text{​}}{\left(\frac{N_{i\alpha }\;}{N_{\alpha }\;}\right)}^{2}}$, where *N*_*i*α_ is the number of individuals carrying allele *i* for SSR α and *N*_α_ is the number of genotyped individuals for SSR α. Alleles were considered rare when they were present in <1% of the individuals.

### Distance analysis

Measuring genetic dissimilarity between diploid or polyploid organisms with dominant markers is still a real challenge (Kosman and Leonard, 2005). The Dice genetic distance (Dice, 1945) appeared to be the most suitable because it overlooks a shared absence of alleles and places more importance on the shared presence of alleles (Babaei *et al.*, 2007). The distances were calculated using 1000 bootstraps with the DARwin v6.0.5 software package (Perrier *et al.*, 2003; Perrier and Jacquemoud-Collet, 2006) on the presence/absence matrix obtained after transformation of the Genemapper export with Genemapper2Darwin, which is a program written in C++ using Bio++ (Guéguen *et al.*, 2013) by S. Gaillard (personal communication). The distances were calculated with 1126 individuals having <20% missing data.

### Structure analysis

A ‘without *a priori* method’, discriminant analysis of principal components (DAPC implemented in the adegenet R package; Jombart, 2008), was conducted on individuals having <30% missing data. It is a clustering method using a few synthetic variables (discriminant functions). This method maximizes differences between groups, and minimizes variation within groups. STRUCTURE gives analogous results (Pritchard *et al.*, 2000). However, the DAPC method, unlike STRUCTURE, is used without requirement for Hardy–Weinberg equilibrium (Jombart *et al.*, 2010) and is therefore well suited for this type of data set, which includes different ploidy levels, ambiguity in the allele copy number for some individuals, overlapping generations, uncertainty of marker inheritance patterns in polyploids, and deviation from panmixis due to a sample that contains related individuals (Vallejo-Marin and Lye, 2013).

First, the K-means method was used to define groups. This method consisted of running several K-means (here, from 2 to 35), with the most probable group number (K) being inferred from the smallest value in the Bayesian information criterion (BIC). Since the optimal K number varied between each of these runs, the optimal K was calculated 1000 times and the most common value was chosen. DAPC analysis was conducted while retaining 13 principal components explaining 36.3% of the total variance. This corresponded to the optimal value according to the a-score optimization procedure proposed in the DAPC tutorial (Jombart, 2013). The analysis gave, for each individual, the probability of membership in the different groups. An individual was considered to be well assigned to its genetic group when the membership probability was >0.8.

As some of our results show genetic structuration according to the ploidy level, a simulation framework was designed in order to test for a bias for ploidy level on the detection of genetic structure. Microsatellite data sets for 10 populations with a low genetic differentiation level were simulated, from which individuals with different ploidy levels were generated. The framework’s hypothesis is that no ploidy effect on genetic structure would mean a genetic clustering according to the initial simulated populations whatever the ploidy level, rather than according to a similar ploidy level whatever the origin of the simulated individuals. Ten subpopulations have been simulated using the software DIY-ABC 1.0.4.36 (Cornuet *et al.*, 2008). These subpopulations derived from a unique ancestral population 50 generations ago. The size of each subpopulation was stable over time and is 500 individuals. The simulation outputs are alleles for 32 autosomal SSR markers obtained for 10 samples, one per subpopulation, of 100 haploid individuals sampled among a subpopulation. SSR markers evolved according to the default mutation parameter values in the software. By hypothesizing panmictic subpopulations with a strict polysomic inheritance of markers, a sample set of 100 individuals, composed of 20 diploid individuals, 20 triploid individuals, 20 tetraploid individuals, 20 pentaploid individuals, and 20 hexaploid individuals, has been created. The genotype of each individual has been obtained by the sampling of as many alleles as the ploidy level, among alleles of the subpopulation. The matrix of co-dominant genotyping has then been transformed into a matrix of presence/absence for each allele, like the one used for the empiric data set of roses. This sample has been studied by a DAPC, as explained for empirical data.

The difference between genetic groups in empirical data was statistically tested by an analysis of molecular variance (AMOVA) calculated with the Dice genetic distance matrix. AMOVA was performed using GenAlEx 6.5 (Peakall and Smouse, 2006, 2012). This analysis partitions the molecular variance on two levels, among and within genetic groups. Pairwise PhiPT were also estimated with 1000 permutations. PhiPT is an analog of Wright’s *F*_ST_ for dominant binary data (Thummajitsakul *et al.*, 2008). It assesses genetic differentiation between genetic groups based only on the genotypic variance, while suppressing the within-population variance (Yamasaki and Ideta, 2013). One thousand permutations were used to determine whether the variance component partitioning was significant. The mean pairwise distance (MPD, i.e. mean of the pairwise PhiPT per genetic group) is an index of the group differentiation relative to other genetic groups. The within-group sum of squares divided by the number of individuals in the group reflects normalized intragroup variability (nSSWG).

Pearson’s χ^2^ tests of independence on a contingency table were performed with the ‘stats’ package in R 3.1.2 (R Core Team, 2014) to determine whether the genetic groups were linked to the collected informative variables (botanical or cultivated, geographic origin, ploidy, breeding year, and horticultural groups). Additional AMOVAs were performed to test (i) whether roses from different periods in Europe were significantly different from each other and different from Asian roses and (ii) the significance of horticultural groups.

## Results

### Genetic diversity and population structure

To check whether the genotyping data were reliable, genetic distances among controls were verified. Repeated controls had a null genetic distance in 93% of cases and 0.0143 was the highest Dice genetic distance among two controls.

The 32 SSRs used to genotype the 1228 genotypes were highly polymorphic, showing from 10 to 75 alleles per primer pair, with a total of 1284 alleles over 32 primer pairs. The percentage of rare alleles was high, namely from 28.9% for H2F12 to 82.4% for RMS124. *Am* ranged from 1.6 for RMS124 to 3.8 alleles for Rw22A3. Despite the high number of alleles, *Ae* was low, ranging from 0.5 for Rw52D4 to 3.8 for RMS082. This means that there were a lot of low frequency alleles (Table 1).

**Table 1.** Indexes of genetic diversity for the studied population

**SSR**	**LG** ^*a*^	**Motif**	**No. observed**	**Missing data (%**)	***Ao***	***Am***	***Ae***	**Rare alleles**
**H9B07** ^*b*^	1	(AAG)6	1198	2.4	21	2.3	0.9	13 (61.9)
**RMS070** ^*c*^	1	GA	1130	8.0	67	2.5	1.7	40 (59.7)
**RMS015** ^*c*^	1	GA	1184	3.6	49	2.7	1.6	21 (42.9)
**Rw25J16** ^*b*^	1	(GA)14	1137	7.4	75	2	3.1	43 (57.3)
**RMS147** ^*c*^	2	AT&GT	1123	8.6	40	1.7	1.7	25 (62.5)
**RMS082** ^*c*^	2	2×GA	1158	5.7	68	2	3.8	35 (51.5)
**Contig172** ^*b*^	2	(AAG)8	1196	2.6	17	1.8	1.1	8 (47.1)
**CTG329** ^*b*^	2	(GAA)10	1166	5.0	17	2.3	0.7	8 (47.1)
**RMS132** ^*c*^	2	GA	1195	2.7	45	2.8	1.6	18 (40)
**Rh80** ^*c*^	2	–	1172	4.6	42	2	2.6	19 (45.2)
**Rw16E19** ^*b*^	3	(TC)11	1193	2.9	31	2.2	1.3	15 (48.4)
**RMS140** ^*c*^	3	GT	957	22.1	19	2.4	0.7	8 (42.1)
**BFACT47** ^*e*^	3	–	1175	4.3	34	2.4	1.5	13 (38.2)
**RMS144** ^*c*^	3	GT	1181	3.8	26	1.9	1.1	14 (53.8)
**CTG21** ^*b*^	3	(TTC)20	1197	2.5	11	1.8	1.3	4 (36.4)
**Rh58** ^*d*^	3	–	1158	5.7	41	1.9	2.5	19 (46.3)
**H20_D08** ^*b*^	4	(TC)10	1144	6.8	56	1.8	3.6	35 (62.5)
**H2F12** ^*b*^	4	(TC)19	1197	2.5	38	2.8	1.4	11 (28.9)
**Rw55E12** ^*b*^	4	(AG)9	1145	6.8	39	2.5	2.3	15 (38.5)
**Rw53O21** ^*b*^	4	(AAG)7	1178	4.1	10	2.3	0.9	4 (40)
**H22F01** ^*b*^	5	(TC)10	1163	5.3	28	2.1	2.1	10 (35.7)
**RMS034** ^*c*^	5	GA	1161	5.5	42	2.7	1.3	19 (45.2)
**Rw52D4** ^*b*^	5	CT rich	1192	2.9	39	3.4	0.5	23 (59)
**CL2980** ^*b*^	6	(AG)16	1144	6.8	29	1.7	2.2	13 (44.8)
**Rw22A3** ^*f*^	6	(TTC)6	1180	3.9	44	3.8	0.8	18 (40.9)
**CTG623** ^*b*^	6	(CT)16	1131	7.9	51	2.8	2.2	19 (37.3)
**Rog9** ^*b*^	6	(AG)13	1184	3.6	53	2	2	33 (62.3)
**H10D03** ^*b*^	7	(TC)11	1170	4.7	46	2.7	1.2	27 (58.7)
**Rw5G14** ^*b*^	7	(AG)7(G)8	1194	2.8	30	3.2	1	10 (33.3)
**RMS003** ^*c*^	7	GA	1152	6.2	48	2.8	1.8	18 (37.5)
**RMS124** ^*c*^	7	GT	1176	4.2	68	1.6	2.1	56 (82.4)
**Rw15D15** ^*b*^	7	(TC)11	1147	6.6	60	1.9	2.4	45 (75)
**Total**			1228		1284		55	677 (50.9)
**Mean**				5.16	40.1	2.3	1.7	20.5 (50.9)

*Ae*, effective number of alleles; *Am*, mean number of alleles per individuals; *Ao*, number of observed alleles; LG, linkage group; No. observed, number of observed genotypes; rare alleles, number of alleles present in <1% of individuals (percentage of the total number of alleles for this SSR).

^*a*^ Named according to the reference sequence published by Spiller *et al.* (2011).

*b* Hibrand-Saint Oyant *et al.* (2008).

^*c*^ Süss and Schultze (2003).

^*d*^ Yan *et al.* (2005).

^*e*^ Gar *et al.* (2011).

^*f*^ Zhang *et al.* (2006).

^*g*^ Meng *et al.* (2009).

**Fig. 4.**
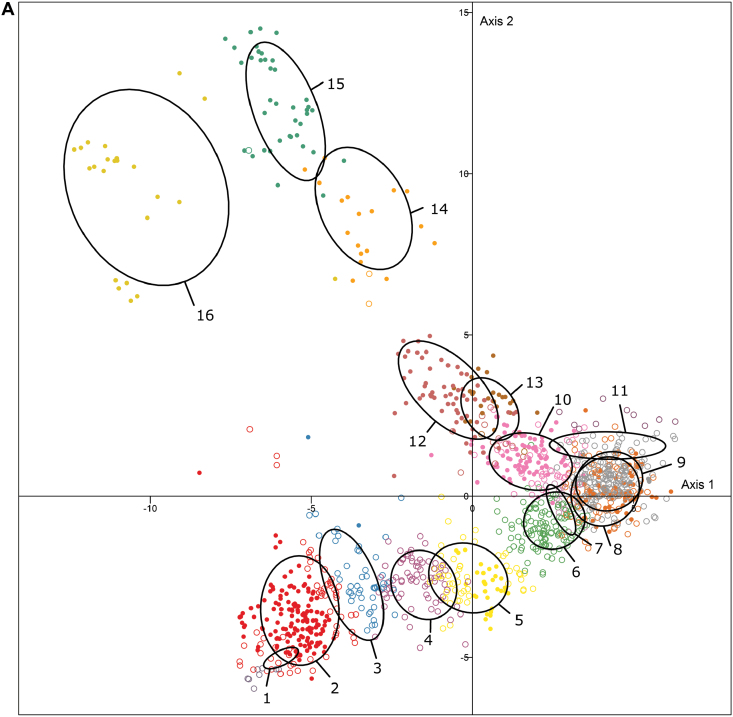

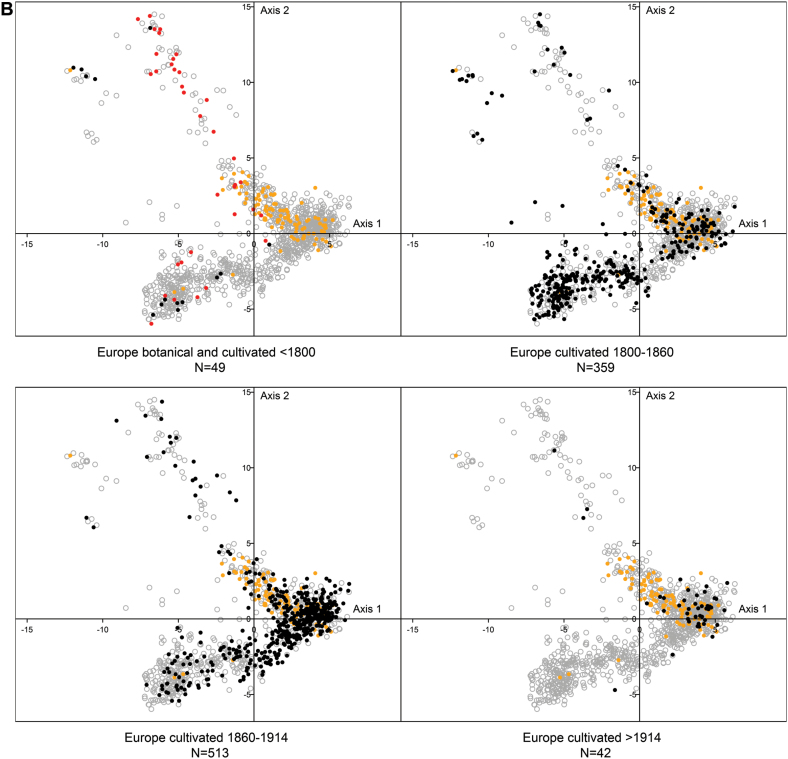

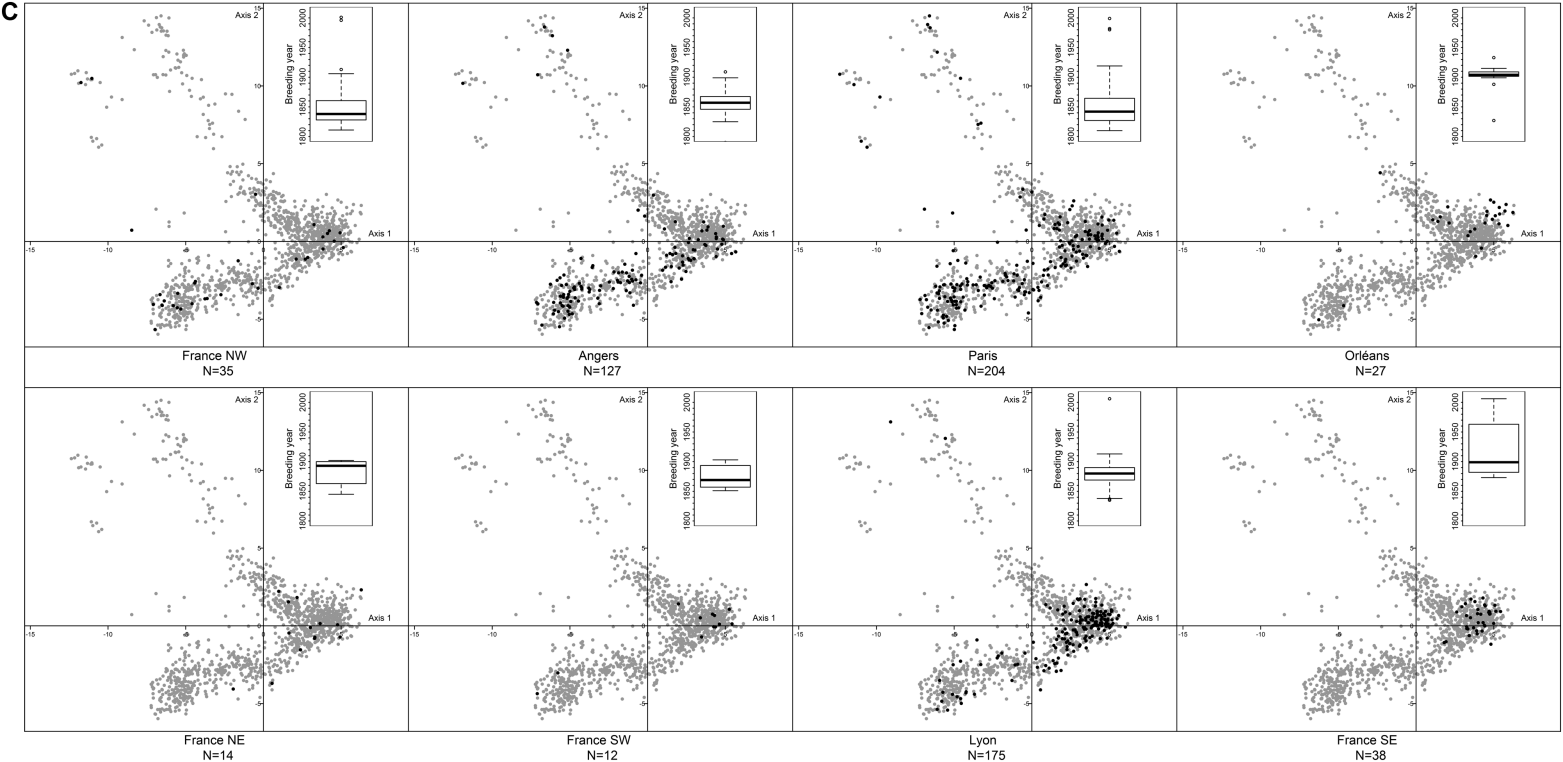

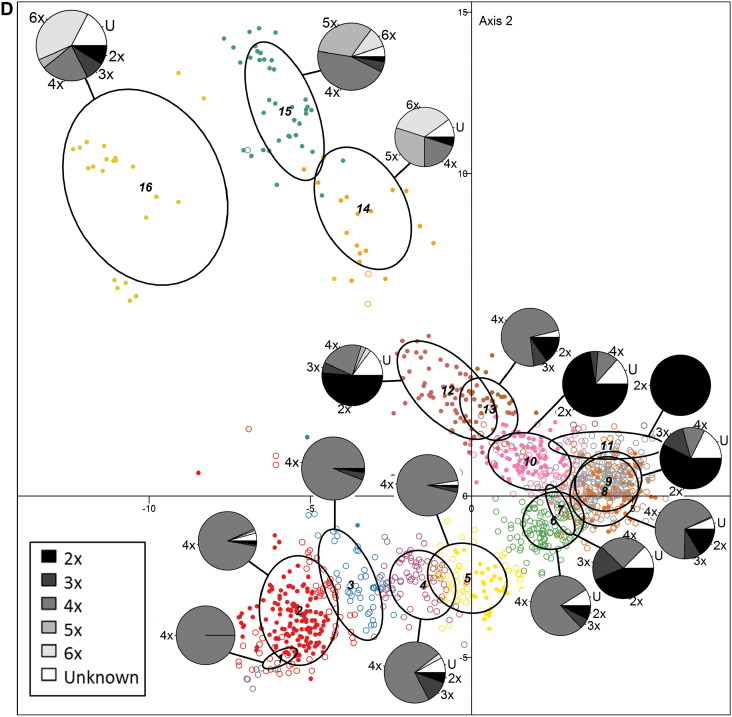
Representation of the first two axes of the discriminant analysis of principal components (DAPC) structuring results of the 1228 individuals. DAPC was made using the first three discriminant functions. The first two axes explain, respectively, 11.2% and 6.6% of the variance. (A) Display of the 16 genetic groups. Each group has a different color; filled circles represent correctly assigned individuals (membership probability ≥0.8), empty circles represent admixed individuals (membership probability <0.8). The ellipses represent the variance of the co-ordinates of the individuals for each group. (B) Geographical and temporal view of the DAPC analysis results. Black filled circles are cultivars created during each period; red filled circles are botanical roses; orange filled circles are Asian roses (botanical and cultivated) and empty circles are roses from other parts of the world or other periods. *n*, number of individuals. (C) Distribution of the French bred roses in the DAPC analysis according to the breeder’s region (black filled circles are cultivars created in each part of France; small circles are cultivars created in other parts of France) with the distribution of the breeding years of roses bred in this region given in the top right-hand corner. *n*, number of individuals. (D) Distribution of ploidy in the genetic groups obtained via DAPC. Individuals with measured ploidy levels and ploidy level found in the literature are presented

The K-means method suggested that the population was structured into 16 genetic groups. The DAPC assigned each individual to the group to which it had the highest probability of belonging and 490 individuals were well assigned (membership ≥0.8) to their group (Fig. 4A; Supplementary Table S2, data available at Zenodo DOI 10.5281/zenodo.56704). The group sizes were unequal, ranging from eight to 210 individuals (Table 2).

**Table 2.** Genetic diversity measures for the 16 genetic groups obtained with DAPC

**Genetic group**	***n***	***Na***	***Nsa***	**MPD** ^***a***^	**nSSWG** ^***a***^
**1**	8	175	0	0.34±0.16	0.13
**2**	210	499	25	0.29±0.13	0.23
**3**	51	460	9	0.21±0.11	0.28
**4**	75	404	8	0.21±0.12	0.26
**5**	79	337	2	0.26±0.14	0.21
**6**	122	425	6	0.27±0.15	0.21
**7**	16	282	5	0.23±0.14	0.28
**8**	142	391	8	0.28±0.15	0.22
**9**	205	472	11	0.25±0.14	0.27
**10**	127	790	89	0.17±0.08	0.37
**11**	10	183	1	0.36±0.15	0.22
**12**	74	811	126	0.22±0.09	0.37
**13**	26	400	23	0.31±0.12	0.29
**14**	20	409	5	0.31±0.12	0.23
**15**	40	417	22	0.35±0.12	0.20
**16**	23	209	1	0.42±0.14	0.11
**Total**	**1228**	**1284**	**341**	**0.3±0.13**	**0.24**

*n*, number of individuals, *Na*, number of alleles; *Nsa*, number of specific alleles; MPD, mean pairwise difference; nSSWG: normalized sum of squares within group

^*a*^Those indexes were calculated with the 1126 individuals having <20% of missing data

Differentiation between genetic groups was statistically significant, as shown by the AMOVA (*P*=0.001), performed on 1126 individuals (Supplementary Tables S3, S4). The variation was partitioned between within-group variability (73%) and intergroup variability (27%). The high within-group variability is a reminder that the genetic groups are constituted by genetically distinct individuals and the significant intergroup variability suggests a limited gene flow between them. The mean pairwise distance ranged from 0.17 for group 10 to 0.42 for group 16 (Table 2).

### Identification of two geographical diversity sources

The first hypothesis tested in this study was the existence of several sources of diversity from at least two areas in the world, namely Europe and Asia. Most of the botanical and bred roses from Asia (77.22%) were in groups 9, 10, and 12, whereas 61% of the European bred roses created before 1860 belonged to groups 2 and 4 (Fig. 4B; Table 3). AMOVA confirmed that the European genetic background of roses bred before 1810 was significantly different from the Asian genetic background (pairwise PhiPT=0.1, *P*<0.01).

**Table 3.** Main composition of the 16 genetic groups revealed by χ^2^ tests

		Botanical/cultivated χ^2^=385.57, *P*<2.2e-16			Horticultural group χ^2^=787.28, *P*<2.2e-16			Breeding year χ^2^=2702.13, *P*< 2.2e-16			Geographic origin χ^2^=1204.21, P<2.2e-16			Ploidy level χ^2^= 1219.27, P< 2 .2e-16		
Genetic group	*n*		% genetic group	% category		% genetic group	% category		% genetic group	% category		% genetic group	% category		% genetic group	% category
1	8				C	37.5	9.4	<1840	100	2.6				4x	100	1.2
2	210	Cultivated	97.6	18.5	HGal	47.6	64.1	[1840–1849]	25	45.5	France Angers	23.3	33.1	4x	94.5	28.4
					M	19.5	46.6	[1810–1819]	15.7	47.8	France NW	8.3	42.9			
											France Paris	29.4	26			
3	51	Cultivated	96.1	4.4	M	23.5	13.6	[1820–1849]	56.3	10.4	France Paris	44.2	9.3	4x	94.1	7.2
					Misc_OGR	5.9	33.3	[1860–1869]	14.6	7.1	Europe S	7	25			
					D	7.8	19									
4	75	Cultivated	98.7	6.7	D	8	28.6	[1850–1859]	19.2	17.3	France Angers	24.6	13.4	4x	78.3	8.1
					M	20	17	<1800	13.7	21.7						
5	79	Cultivated	100	7.1	HP	58.2	27.7	[1850–1889]	69.2	14.3	France Paris	29.9	11.3	4x	96.1	11.1
6	122	Cultivated	99.2	10.9	HP	45.9	33.7	[1860–1899]	70.6	19.6	France Lyon	23.5	16	4x	85.6	14.2
7	16															
8	142	Cultivated	99.3	12.7	HT	36.6	73.2	>1900	42.5	30.5	France SE	9.2	34.2	4x	72.2	14.4
9	205	Cultivated	99	18.3	T	31.2	68.1	[1870–1899]	64.9	28.1	France Lyon	31.8	35.4	2x	69.8	37.5
					Ch	12.7	72.2				France SE	10.8	55.3	3x	16	33.8
10	127	Botanical (*Synstylae*)	20.5	22	HSem	8.7	78.6	>1900	34.7	17.7	China	16.1	29.2	2x	83.6	29.2
		Cultivated	79.5	9.1	Ayr	4.7	100				Asia	11	31.7			
					Pol	11.8	48.4									
11	10	Cultivated	90	0.8	HWich	70	18.9	[1900–1909]	66.7	5.4	France Orleans	40	14.8	2x	100	3.2
12	74	Botanical (*Cinnamomeae* and *Pimpinellifoliae*)	60.8	38.1	Sp	59.5	37.3				China	26.1	27.7	2x	60.3	12.1
											Asia	18.8	31.7			
											America	15.9	17.7			
13	26	Botanical (*Pimpinellifoliae*)	38.5	8.5	HSpn	46.2	70.6				Middle-East	33.3	38.1	4x	76	2.8
											Europe N	37.5	8.3			
14	20	Cultivated	75	2.2	HEg	40	66.7	[1890–1899]	46.7	5.3	Europe N	45	8.3	6x	38.9	25.9
											Europe E	15	16.7	5x	33.3	24
15	40	Botanical (*Caninae*)	40	13.6	Sp	40	13.6				Europe E	23.7	50	5x	34.2	52
16	23	Cultivated	91.3	1.9	A	47.8	40.7	<1700	19.1	19.1	Europe	13.6	25	6x	47.4	33.3
								[1810–1829]	38.1	9.5						

For each characteristic of the genetic group, the percentage of individuals of the category among the genetic group and among the category is displayed.

For example, in genetic group 1 (*n*=8), 100% of the individuals belong to the category <1840 (8/8), while 2.6% of the total sample within the category <1840 belong to genetic group 1 (8/315). When nothing is specified, this means that there is no main trend. Group 7 is too small and too heterogeneous to give significant results.

*n*, number of individuals; A, Alba; Ayr, Ayrshire; C, Centifolia; Ch, China; D, Damask; HCh, Hybrid China; HEg, Hybrid Eglanteria; HGal, Hybrid Gallica; HP, Hybrid Perpetual; HSem, Hybrid Sempervirens; HSpn, Hybrid Spinosissima; HT, Hybrid Tea; HWich, Hybrid Wichurana; Misc OGR, Miscellaneous OGR; M, Moss; Pol, Polyantha; Sp, Species; T, Tea.

These two putative sources of diversity corresponded to genetic groups with high intragroup diversity and are clearly separated on the first axis in Fig. 4A. nSSWG (Table 2) was high in genetic groups 3 and 4 (majority of European roses), situated on the left in Fig. 4A, and much higher in genetic groups 10 and 12 (majority of Asian roses), situated on the right in Fig. 4A. In between, in groups 5–8, there was a decrease in nSSWG, indicating a decrease in intragroup genetic diversity. Group 7 was too small (16 individuals) and too heterogeneous to give significant results.

### A genetic structure linked to breeding periods

The second hypothesis of this study was to determine whether the genetic background of rose cultivars changed during the 19th century. The χ^2^ test findings highlighted the non-independence (*P*<0.01) between genetic groups obtained with DAPC and the cultivar breeding dates (Fig. 4B). Genetic groups 1–5 included 61% of genotypes created before 1860, therefore before the creation of the first Hybrid Tea (‘La France’ in 1867). In contrast, groups 6, 8–11, and 14 included 71% of genotypes created after 1860 (Table 3).

An AMOVA performed on the European pool, divided in 13 temporal classes, indicates that the earlier the roses were obtained in the 19th century, the more they are different from the roses obtained at the end of the 19th century, after 1870 (Table 4). Roses bred before 1810 were clearly distinct from modern European roses bred after 1914 (PhiPT=0.16, *P*≤0.002). These results showed that the genetic diversity of European roses evolved during the 19th century.

**Table 4.**
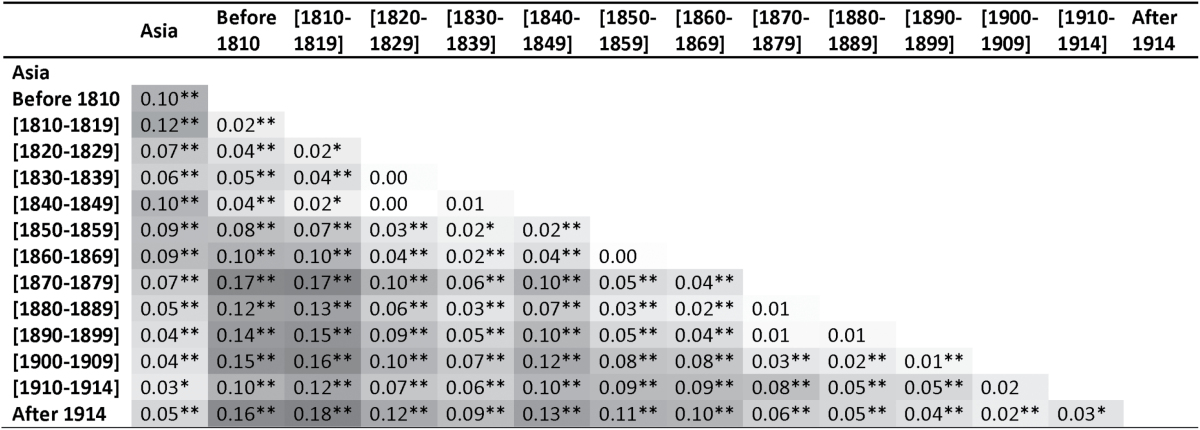
Pairwise PhiPT values based on the Dice genetic distance comparing the European pool divided in 13 temporal classes and the Asian pool

The shading varies from white to dark gray according to the height of the PhiPT value. A high PhiPT means a high distance between groups.

**P≤0.002, *P≤0.005

**Fig. 5.**
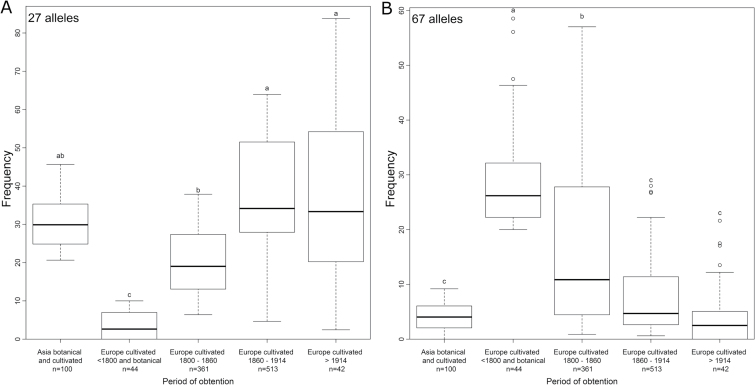
Evolution of the frequency of occurrence of the alleles in the sample. The boxplots show the median and the box delimits the interquartile interval. The letters represent the result of a Tukey’s HSD test comparing each group of individuals (*P*<0.01). *n*, number of individuals in this period. (A) Alleles present at a high frequency (>20%) in roses from Asia and at a null or low frequency (<10%) in European roses bred before 1800. (B) Alleles present at a high frequency (>20%) in European roses bred before 1800 and at a null or low frequency (<10%) in roses from Asia.

The increasing proximity of the genetic groups containing more genotypes bred after 1860 (6, 8–11, and 14, see above) to the genetic groups containing Asian roses (9, 10, and 12) strongly suggested that the late 19th century hybrids were increasingly related to Asian roses. This was confirmed by AMOVA (Supplementary Table S5), showing a decreasing pairwise PhiPT, and therefore a higher genetic proximity, when Asian roses were compared with increasingly recent European roses (from PhiPT=0.1 between Asian roses and European roses bred before 1810 to PhiPT=0.05 between Asian roses and European roses bred after 1914, *P*<0.01, Table 4). This was also noticeable in the allele frequency analysis findings. Twenty-seven alleles were present at high frequency (>20%) in roses from Asia and at a null or low frequency (<10%) in European roses bred before the 19th century. During the 19th century, their frequencies increased until the frequency in roses bred after 1914 reached the frequency in Asian roses (Fig. 5A). The inverse trend was observed for 67 alleles frequent in European roses before the 19th century and rare in roses from Asia (Fig. 5B).

Interestingly, this temporal shift has influenced the genetic diversity used in each French region throughout the 19th century according to their golden age (Fig. 4C). Roses from Angers and Paris, which were bred in the early 19th century, were mainly present in groups 2–5 (53.92% of the Parisian roses and 60.63% of the roses from Angers). Groups 6 and 9 contained 51.43% of the roses selected in the region around Lyon, which has been a rose-breeding hotspot in France since 1825. They were situated close to roses from Asia.

### Ploidy levels and genetic groups

The proportion of different ploidy levels in the sample was 28% diploids, 7% triploids, 60% tetraploids, 2% pentaploids, and 2% hexaploids.

Ploidy level was significantly linked to the genetic groups (χ^2^ test, *P*<2.2e-16; Fig. 4D; Table 3). The clustering method was checked using simulated data sets to ensure an absence of bias for clustering according to ploidy levels. The DAPC analysis of the simulated sample with mixed ploidy levels coded as presence/absence of alleles showed a clustering pattern generally in line with that of the initial simulated subpopulations (Supplementary Fig. S1). AMOVA confirmed that the origin (20% of molecular variance explained among subpopulations, *P*=0.001) of individuals had more impact on the detection of population structure than the ploidy level (4%). The probability of membership in the correct group was higher and showed lower variance for increasing ploidy levels, probably because individuals with high ploidy levels contained more information from the simulated subpopulations (Supplementary Fig. S2). This analysis confirmed that the clustering results obtained from the empirical data set were reliable and that the method could be applied on real data.

Groups 9–12 (on the right in Fig. 4A) contained 84.1% of the diploid genotypes, and groups 1–6, 8, 13, and 15 (towards the left in Fig. 4A) contained 90% of the tetraploid genotypes. At the junction between groups containing tetraploid roses and groups containing diploid roses, groups 4, 8, and 9 contained more than half of the triploid genotypes (65.2%). This may indicate that they were the outcome of crosses between diploid and tetraploid roses (Fig. 4D) and it was interesting to note that 56.6% of the triploid roses were created after 1860. Pentaploid genotypes were mainly included in genetic groups 14 and 15 (76%) and hexaploid genotypes were in genetic groups 14 and 16 (59.3%). These three groups were among the most differentiated groups. Based on the pairwise PhiPT and MPD values (Table 2; Supplementary Table S4), groups 15 and 16 were significantly different from all other groups (MPD=0.35 and 0.42, respectively, *P*<0.01) and group 14 was significantly different from all groups except one (MPD=0.31, *P*<0.01).

### Classification in the light of molecular data

The last hypothesis tested here is whether varieties from a given horticultural group share a common genetic background. The horticultural classification was linked to genetic groups (χ^2^ test, *P*<0.01). The AMOVA results (Supplementary Table S6, S7) indicated that the horticultural groups had a lower MPD (from 0.09 to 0.20; Supplementary Table S7) than the genetic groups (from 0.17 to 0.42; Table 2). Teas, Hybrid Gallica, Hybrid Wichurana. and Chinese roses were the most differentiated horticultural groups (MPD=0.20), followed by Centifolia (MPD=0.19), Damasks, Alba, and Hybrid Teas (MPD=0.18), Hybrid Rugosa, Polyanthas, and Bourbons (MPD=0.17), Hybrid Perpetuals (MPD=0.16), and Moss and Noisette roses (MPD=0.14). As expected, the species and interspecific hybrids had a low MPD (0.13 and 0.12, respectively), as they included highly different roses. Interestingly, Hybrid Multiflora had a low MPD of 0.12, possibly due to a high genetic heterogeneity.

Horticultural groups historically known as being close, such as Hybrid Gallica roses (mainly in genetic group 2) and their mutant Moss roses (mainly in genetic groups 2, 3, and 4), were also genetically close. Hybrid Teas (mainly in genetic group 8) are located between the Teas (mainly in genetic group 9) and Hybrid Perpetuals (mainly in genetic groups 5, 6, and 8) which would be their progenitors (Table 3; Supplementary Table S7 for PhiPT values).

Although those results suggest a partial consistency of the horticultural classification, the low PhiPT values between two (or more) consecutive groups showed that there were no clear separations between genetic groups and therefore between horticultural groups as they were present in different genetic groups. Moreover, some horticultural groups were present in two genetically distant groups, such as, for example, Hybrid Gallica roses, which were also present to a lesser degree in group 15 (seven individuals out of 157 Hybrid Gallica roses). The same was noted with Hybrid Perpetual roses which were also present in genetic group 2.

## Discussion

### A shift from a European to an Asian genetic background in cultivated French roses

Despite frequent changes in genetic sources used in breeding programs, temporal variations of genetic structure in cultivated plants have been rarely reported (but see Roussel *et al.*, 2004; Ovesná *et al.*, 2013; Tondelli *et al.*, 2013).

Old European and Asian roses formed two pools along the first axis of the DAPC analysis (Fig. 4B). This confirmed the work of Koopman *et al.* (2008), who showed that rose cultivars can be divided into a European and an oriental cluster. Groups 3 and 4 (Fig. 4A), close to old European roses, and group 10, close to Asian roses, showed the highest diversity. This could be explained by the presence of two sources of diversity in the sample: an ancestral European and an Asian source of diversity (Vavilov, 1951).

Between both pools, groups 5–8 contained hybrids bred in Europe. The diversity of those hybrids was also clearly structured according to the breeding year. The most recent roses (obtained after 1860 in genetic groups 6, 8–11, and 14) were closer to the Asian pool, showing the increasing importance of the genetic background of Asian roses in breeding during the 19th century. This was especially true for the contemporary roses bred after 1914 (Fig. 4B). The shift in the frequency of occurrence of some alleles supports this observation (Fig. 5). These results strongly suggest that the Asian source of diversity was progressively introgressed in the European genetic background. Wylie (1954) considered that four Chinese genotypes were introduced in Europe around 1800 and were at the origin of modern Hybrid Tea roses: ‘Old blush’, ‘Hume’s Blush Tea-Scented China’, ‘Slater’s Crimson China’, and ‘Parks’-Yellow Tea-Scented China’. Those four, according to their denomination in the prospected rose gardens, were present in genetic groups 9 and 10, towards which the European hybrid genetic background converged.

The structure observed here and the pairwise PhiPT showed that, except for genetic groups 15 and 16, most of the groups were not significantly different from their neighboring groups (Supplementary Table S4) and that some cultivars had a small percentage of assignation to their group (Supplementary Table S2, data available at Zenodo DOI 10.5281/zenodo.56704). This continuous structure, with no clear separation between groups, was probably due to the importance of successive crossings between European rose hybrids and Asian roses, leading to genetically close roses. Several genetic groups summarized the information of intermediate steps during the hybridization process and were not true isolated populations. The presence/absence coding of alleles may have led to a loss of information amplifying this phenomenon.

In a context of Old European garden roses dominated by once-flowering varieties (Hurst, 1941), the introduction of continuous-flowering Chinese roses in Europe triggered considerable interest among European breeders. The supposed disappearance of many old once-flowering varieties from rose gardens after the creation of continuous-flowering hybrids demonstrates that this was a target trait for 19th century breeders. The recessive genetic control (Semeniuk, 1971) of this trait could at least partly explain the continuous shift of the hybrids towards an Asian genetic background. The first hybrids between once-flowering European roses and continuous-flowering Chinese roses must have been once-flowering, requiring supplementary crossings with Chinese roses. Continuous-flowering roses were thus obtained with few generations, implying that they are genetically close to Asian roses. Other traits brought by Asian roses such as perfume (Scalliet *et al.*, 2008) may have amplified this trend.

As already mentioned, contemporary roses (bred after 1914) were genetically close to Asian roses and lost some specific old European alleles. It would be interesting to determine what proportion their genome has an Asian origin. This could be done using sequencing or genotyping in order to attribute ancestry to each genome region. As a breeding perspective, with the aim of finding more disease and pest resistance genes for modern roses, it would be interesting to explore the ancestral European pool which is said to be more rustic (Maia and Venard, 1976).

### The history of bred roses reflected by ploidy levels

Our analysis revealed that ploidy was linked to genetic groups, and triploid roses were midway between diploids and tetraploids, probably as a consequence of their hybridization history (Fig. 4D). Indeed, triploid roses appeared to be important bridges between Chinese roses (generally diploids) and Old European roses (generally tetraploids) showing a confounding effect between ploidy levels and geographical origin or breeding history. Among the main horticultural groups, the first Bourbon, Hybrid China, and Hybrid Tea roses were all triploids. Unreduced gametes produced by these almost sterile triploids may have formed tetraploid roses, thus facilitating breeding in the group (Maia and Venard, 1976; Gudin, 2000).

Most hexaploids belong to the horticultural group Alba. Although their origin remains mysterious, they may have been obtained via crosses between *R. canina* (5*x*) and *R. gallica* (4*x*) or *R*. *damascena* (4*x*) (Hurst, 1941; Maia and Venard, 1976). This hypothesis seems convincing as Alba roses were mostly found in genetic group 16, near genetic group 15 consisting of roses from the *Caninae* section. *Gallicae* and damask roses were mainly found in genetic group 2, close to Alba roses on the first DAPC axis, but more distantly than *Caninae* on the second axis. This higher proximity of Alba roses from *Caninae* than from *Gallicae* may lead to the following hypotheses about their genomic composition. Alba roses may have received four chromosome sets from *Rosa canina* as female parent [during an asymmetric meiosis process, mother cells from this pentaploid species have four chromosome sets, while pollen brings only one (Täckholm, 1920; Nybom *et al.*, 2004, 2006)] and two chromosome sets from the pollen of *R. gallica*, *R. damascena*, or a close tetraploid species, resulting in an hexaploid individual.

Genetic groups 14–16, containing mainly penta- and hexaploid genotypes, were more distant from the ‘hotspot’ of cultivated roses. This suggests that they were less used in breeding, probably because their ploidy level makes them difficult to cross with other diploid or tetraploid roses. This distance may also be due to the fact that they have less interesting traits for breeders.

### Regional exchanges and relationships among French breeders

The genetic proximity of roses from the same region in France may be explained by the fact that breeders in a given region may have used accessions with a close genetic background or have exchanged accessions with other breeders in the region. This has been shown for breeders from the Lyon region (Ferrand, 2015) and may be true for other regions too.

In the sample, nearly 60% of the roses from the Angers region were bred before 1860 and the number declined after this year, with a slight rebound after 1880, whereas in Lyon 90% of the roses were bred after 1860. Combined with the fact that roses from Lyon were closer to the Asian pool, the success of roses bred in Lyon may be one reason for the decline in rose breeding in Angers among other reasons (economic, political, etc.).

### A still controversial classification

Since the early 19th century, there have been many attempts to classify roses, but the resulting classifications are not consensual (Wissemann, 2003). Two types of classifications exist: a botanical classification for wild species and a horticultural one for cultivated roses. Obviously they are not independent.

Scariot *et al.* (2006) studied genetic relationships in 65 old garden roses and concluded that the genetic clustering was in agreement with the botanical classification and with the horticultural literature. In the present sample, although there were some visible trends such as for the Teas or Hybrid Gallica roses (Table 3), our results show a weak correlation between genetic groups and horticultural groups. This could be due to errors in the attribution of roses to horticultural groups, an inaccurate horticultural classification that is not genetically founded or detectable with those markers, or simply the absence of genetic bases for some horticultural groups. The weak correlation is particularly true for Noisette, Moss, and Hybrid Multiflora horticultural groups. The Noisette and Hybrid Multiflora groups are more recently created and the parents may be already highly hybridized. In the horticultural classification, Moss roses were grouped essentially because of the mossy phenotypic trait without taking other traits into account. This trait may have been introduced in different genetic backgrounds.

More generally, breeders do not focus on a single horticultural group and may use very different backgrounds to create their varieties, adding complexity to the genetic relationships (L. Crespel, personal communication). Hence, based on our findings, the current horticultural classification of cultivated roses should be reconsidered from a genetic point of view. As it was created based on phenotypic traits, the classification is well suited for commercial purposes. For breeding and preservation purposes, roses should be classified according to both genetic and distinct morphological markers. However, cultivated plants will always be hard to classify as their relationships are more complex than those of wild plants (Malécot, 2013).

## Supplementary data

Supplementary data are available at *JXB* online, except Table S2.

Table S1. Rose Garden addresses.

Table S2. Informative data about the sample. Data available at Zenodo DOI 10.5281/zenodo.56704

Table S3. AMOVA results based on the Dice genetic distances and the genetic groups obtained with DAPC.

Table S4. Pairwise PhiPT values based on the Dice genetic distance and the genetic groups obtained with DAPC.

Table S5. AMOVA results based on the Dice genetic distances, the European pool divided in 13 temporal classes, and the Asian pool.

Table S6. AMOVA results based on the Dice genetic distances and the horticultural groups.

Table S7. Pairwise PhiPT values based on the Dice genetic distance and the horticultural groups.

Figure S1. Membership probability, obtained by DAPC analysis, of simulated individuals classified according their original subpopulation.

Figure S2. Boxplot for correct assignation of simulated individuals according to their ploidy level.

Notes S1. Historical sources.

Supplementary Data
