# Supplementary material for: Nineteenth century French rose (Rosa sp.) germplasm shows a shift over time from a European to an Asian genetic background
Source: J Exp Bot. 2016 Jul 12;67(15):4711–25. doi: 10.1093/jxb/erw269 (PMC4973750; doi:10.1093/jxb/erw269)
Supplement: Supplementary Data [file supp_67_15_4711__index.html]

Nineteenth century French rose (Rosa sp.) germplasm shows a shift over time from a European to an Asian genetic background — Supplementary Data 

# Nineteenth century French rose (*Rosa* sp.) germplasm shows a shift over time from a European to an Asian genetic background

## Supplementary Data

Data files

- supplementary\_figures\_S1\_S2\_Tables\_S3\_S7.pdf - Supplementary Data
- supplementary\_table\_S2.pdf - Supplementary Data
