# Supplementary material for: Nineteenth century French rose (Rosa sp.) germplasm shows a shift over time from a European to an Asian genetic background
Source: J Exp Bot. 2016 Jul 12;67(15):4711–25. doi: 10.1093/jxb/erw269 (PMC4973750; doi:10.1093/jxb/erw269)
Supplement: Supplementary Data [file supp_erw269_supplementary_figures_S1_S2_Tables_S3_S7.pdf]

Journal of Experimental Botany **Supporting Information**

Article title: Nineteenth century French rose (Rosa sp.) germplasm shows a shift over time from a European to an Asian genetic background

Authors: Mathilde LIORZOU, Alix PERNET, Shubin LI, Annie CHASTELLIER, Tatiana THOUROUDE, Gilles MICHEL, Valéry MALECOT, Cristiana OGHINA-PAVIE, Céline BRIEE, Fabrice FOUCHER, Jérémy CLOTAULT, Agnès GRAPIN

The following Supporting Information is available for this article:

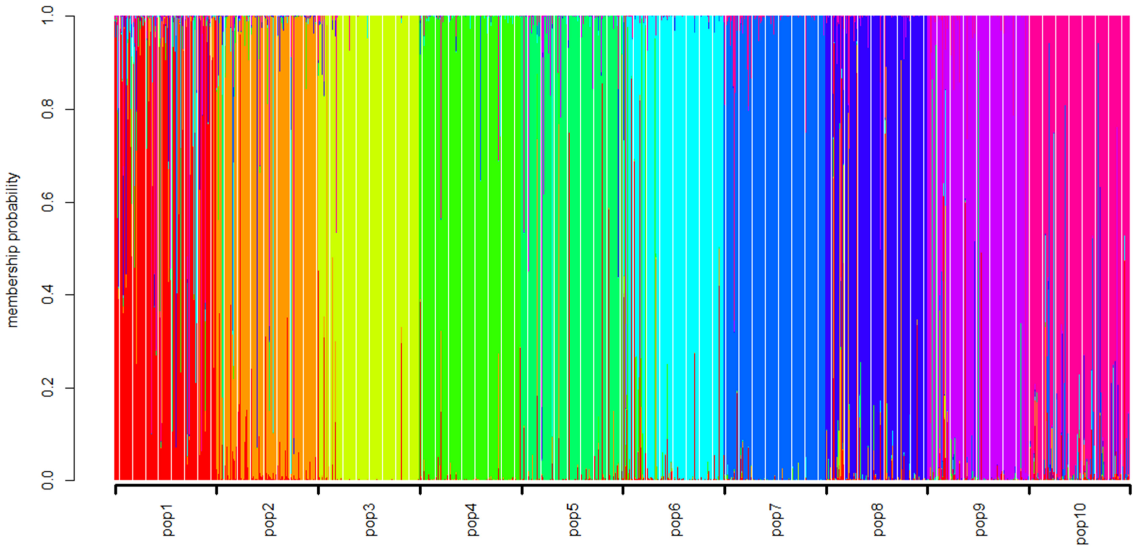

Fig S1 Membership probability, obtained by DAPC analysis, of simulated individuals classified according their original subpopulation ("pop1" to "pop10"). For each subpopulation, 100 simulated individuals are depicted according the following order: 20 diploids, 20 triploids, 20 tetraploids, 20 pentaploids and 20 hexaploids. Each color in the plot represents the membership to a given genetic cluster, determined by DAPC.

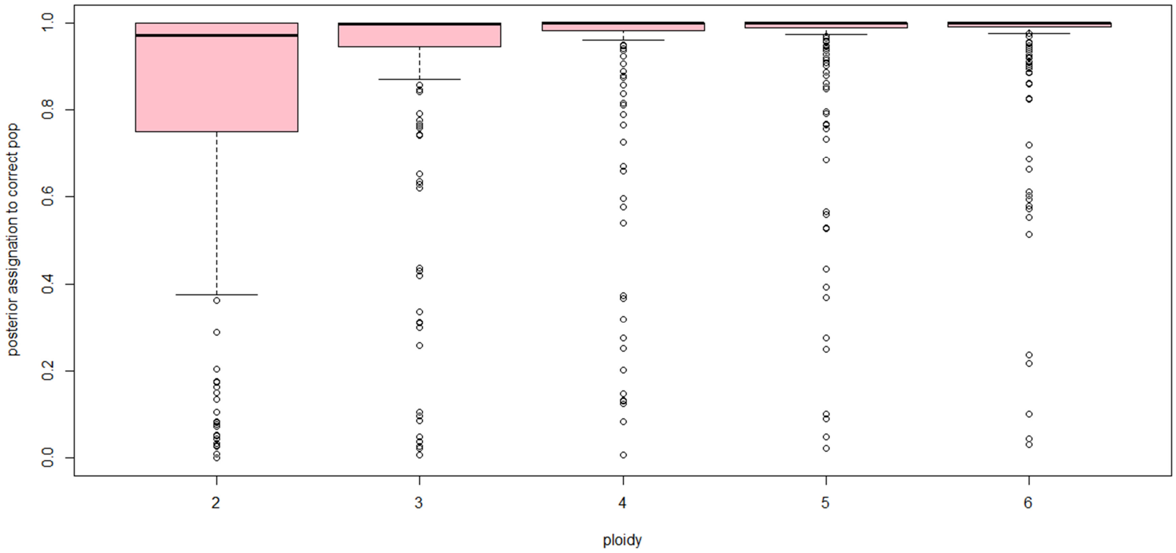

Fig S2 Boxplot for correct assignation of simulated individuals according to their ploidy level. Whatever their original subpopulation, simulated individuals are represented on the x-axis according to their ploidy level. The membership probability for the correct original subpopulation is represented in the y-axis. The box represents the interquartile range (IQR) and the bold trait is the median. The lower whisker represents first quartile - 1,5 IQR. Points are outliers.

Notes S1 Historical sources

The following historical sources were used to check the date of breeding of the different cultivars studied.

Boitard P. **1836**. *Manuel de l'amateur de roses leur monographie, leur histoire et leur culture par M. Boitard*. Paris, France: Roret.

Bosc d'Antic L-A. **1809**. Rosier. In : Thouin, Tessier, Huzard, Silvestre, Bosc, Yvart, Chassiron, Chaptal, De Lacroix, De Perthuis *et al. Nouveau cours complet d'agriculture théorique et pratique, contenant la grande et la petite culture, l'Economie rurale et domestique, la médecine vétérinaire, etc. ou Dictionnaire raisonné et universel d'agriculture*. Paris, France: Déterville, 237–266.

Calvert. **1821**. *Catalogue of Roses cultivated at Calvert and Company's Nursery*. Rouen, France: Bonne nouvelle.

Cochet P. **1877**. *Journal des roses (Rosa inter Flores) Publication mensuelle spéciale fondée par M.S. Cochet horticulteur rosiériste*. Melun, France.

Cochet P, Mottet S. **1909**. *Les Rosiers, historique, classification, nomenclature, description, culture*. Paris, France: Doin.

Desportes N. **1829**. *Roses cultivées en France au nombre de 2562 espèces ou variétés avec la synonymie française et latine*. Le Mans, France: Pesche.

Graveraux J. **1902**. *Roseraie de l'Haÿ (Seine): les roses cultivées à l'Haÿ en 1902 ; essai de classement avant-propos de André Theuriet*. Paris, France: Cochet-Rousset.

Graveraux J. **1912**. *La Malmaison. Les Roses de l'Impératrice Joséphine*. Paris, France: Editions d'art et de littérature.

Guillemeau J-L-M. **1800**. *Histoire naturelle de la rose: où l'on décrit ses différentes espèces, sa culture, ses vertus et ses propriétés; suivie de la corbeille de roses, ou choix de ce que les anciens et les modernes ont écrit de plus gracieux sur la rose; et de l'histoire des insectes qui vivent sur le rosier*. Paris, France: Vatar-Jouannet.

Lindley J. **1820**. *Rosarum Monographia; Or, A Botanical History of Roses. To Which is Added an Appendix for the Use of Cultivators, in Which the Most Remarkable Garden Varieties are Systematically Arranged*. London: Ridgeway.

Lindley J. **1824**. *Monographie du genre rosier: traduite de l'anglais de M. J. Lindley, avec des notes de M. L. Joffrin, et des changements importants, suivie d'un appendice sur les roses cultivées dans les jardins de Paris et environs par M. de Pronville*. Paris, France: Audot.

Noisette LC. **1825**. *Catalogue des rosiers, dahlia, camellia, chrysanthèmes et paeonia cultivés dans les jardins et pépinières*. Paris, France: Rousselon; Mme Huzard.

Noisette L. **1826**. *Catalogue général des arbres, arbustes et plantes, tant de serre que de pleine terre, cultivés dans les jardins et pépinières de L. Noisette*. Paris, France: Rousselon-Huzard.

Prevost. **1829**. *Catalogue descriptif, méthodique et raisonné, des espèces, variétés et sous-variétés du genre rosier, cultivées chez Prévost fils pépiniériste à Rouen*. Rouen, France: Nicétas Périaux le Jeune.

Pronville A de. **1818**. *Nomenclature raisonnée des espèces, variétés et sous-variétés du genre Rosier: observées au Jardin royal des plantes, dans ceux de Trianon, de Malmaison, et dans les pépinières des environs de Paris*. Paris, France: Huzard.

Redouté P-J, Thory C-A. **1817**. *Les Roses*. Paris, France: Firmin Didot.

Rivers T. **1863**. *Catalogue of Roses Edition for Autumn 1863, Thirtieth Edition & Descriptive Catalogue of Selected Roses cultivated for sale by Thomas Rivers & Son, Sawbridgeworth, Herts*. London, UK: Taylor.

Simon L, Cochet P. **1899**. *Nomenclature de tous les noms de roses connus, avec indication de leurs races, obtenteur, année de production, couleur et synonymes*. Metz, France: Béha.

Singer M. **1885**. *Dictionnaire des roses, ou, Guide général du rosiériste*. Tournai, Belgique: Author.

SNHF. **1827**. *Annales de la société d'horticulture de Paris et journal spécial de l'état et des progrès du jardinage*. Paris, France: SNHF.

Souchet C-D. **1846**. *Catalogue et prix-courant pour l'année 1846, à l'Etablissement horticole de Souchet Fils*. Versailles, France: Klefer.

Vibert J-P. **1824**. *Observations sur la nomenclature et le classement des rosiers, suivies du catalogue de celles cultivées par J-P. Vibert à Chennevières-sur-Marne*. Paris, France: Huzard.

Vibert J-P. **1831**. *Observations sur la nomenclature et le classement des rosiers, suivies du catalogue de celles cultivées par J-P. Vibert à Saint-Denis (Seine)*. Paris, France: Huzard.

Table S1: Rose Garden Addresses

Garden	Address	Post code	Town	Country
Loubert rose garden	11, rue de la société	49350	Les Rosiers-sur-Loire	France
Val de Marne rose garden	Rue Albert Watel	94240	L'Haÿ-les-Roses	France
La Cour de Commer rose garden	La Cour	53470	Commer	France
Jumaju rose garden	Les Fieffes	14350	Montchamp	France
Jardin botanique de la Tête d'Or		69006	Lyon	France
Désert rose garden	Lieu dit 'Panjas'	32290	Bouzon-Gellenave	France
Grande roseraie de Lyon		69006	Lyon	France
La Beaujoire rose garden	Rte. de St Joseph	44000	Nantes	France
SCRADH	727 avenue Alfred Décugis	83400	Hyeres	France
Arboretum des Barres	Domaine des Barres	45290	Nogent-sur-Vernisson	France
Flower Research Institute	Yunnan Academy of Agricultural Sciences	650205	Kunming	China

Table S3: AMOVA results based on the Dice genetic distances and the genetic groups obtained with DAPC

Source of variation	df	Sum of Square	Mean sum of square	Estimated Variance	% of the variance	P(rand >= data)
**Between Pops**	15	98.14	6.54	0.09	27%	0.001
**Within Pops**	1110	288.14	0.26	0.26	73%	
**Total**	1125	386.28		0.35	100%	

Table S4: Pairwise PhiPT values based on the Dice genetic distance and the genetic groups obtained with DAPC

	Grp1	Grp2	Grp3	Grp4	Grp5	Grp6	Grp7	Grp8	Grp9	Grp10	Grp11	Grp12	Grp13	Grp14	Grp15	Grp16
**Grp1**																
**Grp2**	0.11															
**Grp3**	0.14	0.08														
**Grp4**	0.2	0.13	0.07													
**Grp5**	0.32	0.25	0.16	0.07												
**Grp6**	0.4	0.34	0.23	0.14	0.08											
**Grp7**	0.37	0.34	0.2	0.15	0.2	0.13										
**Grp8**	0.44	0.39	0.29	0.23	0.21	0.14	0.14									
**Grp9**	0.41	0.38	0.28	0.23	0.24	0.17	0.06	0.09								
**Grp10**	0.27	0.27	0.16	0.14	0.19	0.17	0.05	0.16	0.11							
**Grp11**	0.54	0.46	0.36	0.38	0.45	0.44	0.29	0.38	0.28	0.17						
**Grp12**	0.28	0.29	0.18	0.22	0.29	0.3	0.18	0.3	0.26	0.11	0.24					
**Grp13**	0.41	0.38	0.29	0.32	0.41	0.41	0.29	0.4	0.34	0.2	0.34	0.12				
**Grp14**	0.43	0.35	0.26	0.29	0.37	0.38	0.31	0.39	0.35	0.21	0.41	0.17	0.29			
**Grp15**	0.49	0.4	0.33	0.35	0.43	0.43	0.39	0.43	0.4	0.26	0.46	0.21	0.35	0.23		
**Grp16**	0.6	0.38	0.35	0.39	0.48	0.49	0.5	0.49	0.46	0.32	0.62	0.3	0.46	0.46	0.42	

PhiPT values below diagonal, all probability (based on 1000 permutations) were ≤ 0.001.

In grey: PhiPT< 0.2, less differentiated genetic groups

Table S5: AMOVA results based on the Dice genetic distances, the European pool divided in 13 temporal classes and the Asian pool.

Source of variation	df	Sum of Square	Mean sum of square	Estimated Variance	% of the variance	P(rand >= data)
**Among Regions**	1	3.17	3.17	0.01	2%	0.001
**Among Pops**	12	21.37	1.78	0.02	6%	0.001
**Within Pops**	963	306.15	0.32	0.32	92%	
**Total**	976	330.69		0.34	100%	

Table S6: AMOVA results based on the Dice genetic distances and the horticultural groups

Source of variation	df	Sum of Square	Mean sum of square	Estimated Variance	% of the variance	P(rand >= data)
**Among Pops**	17	64.75	3.81	0.06	17%	0.001
**Within Pops**	1108	321.53	0.29	0.29	83%	
**Total**	1125	386.28		0.35	100%	

Table S7: Pairwise PhiPT values based on the Dice genetic distance and the horticultural groups

	nSSWG	MPD	T	Ch	HT	Pol	HWich	N	B	intersp	HMult	HP	HRg	A	D	HGal	C	M	Sp	Others
**T**	*0.28*	*0.20*																		
**Ch**	*0.36*	*0.20*	0.12																	
**HT**	*0.22*	*0.18*	0.09	0.16																
**Pol**	*0.25*	*0.17*	0.16	0.14	0.19															
**HWich**	*0.23*	*0.20*	0.25	0.24	0.25	0.14														
**N**	*0.26*	*0.14*	0.08	0.08	0.08	0.11	0.19													
**B**	*0.26*	*0.17*	0.18	0.15	0.09	0.20	0.26	0.07												
**intersp**	*0.33*	*0.12*	0.09	0.10	0.06	0.12	0.17	0.06	0.11											
**HMult**	*0.24*	*0.12*	0.18	0.15	0.16	0.04	0.09	0.10	0.15	0.09										
**HP**	*0.33*	*0.16*	0.23	0.19	0.12	0.22	0.26	0.13	0.05	0.12	0.15									
**HRg**	*0.22*	*0.17*	0.24	0.23	0.23	0.17	0.16	0.17	0.22	0.13	0.10	0.21								
**A**	*0.30*	*0.18*	0.33	0.33	0.30	0.25	0.24	0.24	0.26	0.20	0.16	0.21	0.19							
**D**	*0.29*	*0.18*	0.36	0.35	0.32	0.27	0.26	0.25	0.26	0.23	0.18	0.19	0.21	0.07						
**HGal**	*0.29*	*0.20*	0.36	0.34	0.31	0.30	0.29	0.28	0.27	0.26	0.21	0.20	0.26	0.10	0.04					
**C**	*0.28*	*0.19*	0.35	0.34	0.31	0.28	0.28	0.24	0.24	0.24	0.19	0.17	0.23	0.11	0.02	0.06				
**M**	*0.29*	*0.14*	0.29	0.28	0.23	0.23	0.22	0.20	0.18	0.18	0.14	0.12	0.18	0.07	0.02	0.03	0.03			
**Sp**	*0.39*	*0.13*	0.22	0.21	0.21	0.14	0.14	0.16	0.20	0.12	0.09	0.19	0.06	0.09	0.12	0.16	0.15	0.11		
**Others**	*0.24*	*0.09*	0.15	0.13	0.11	0.09	0.11	0.08	0.10	0.05	0.04	0.09	0.08	0.08	0.09	0.12	0.10	0.07	0.04	

PhiPT values below diagonal, all probability (based on 1000 permutations) were ≤ 0.001.

In grey: PhiPT< 0.2, less differentiated horticultural groups
