# Supplementary material for: Nineteenth century French rose (Rosa sp.) germplasm shows a shift over time from a European to an Asian genetic background
Source: J Exp Bot. 2016 Jul 12;67(15):4711–25. doi: 10.1093/jxb/erw269 (PMC4973750; doi:10.1093/jxb/erw269)
Supplement: Supplementary Data [file supp_erw269_supplementary_table_S2.pdf]

Table S2: Informative data about the sample

Name	Origin of the sample	genetic group	Percentage of assignation	Likely year (years of breeding for bred roses years of introduction for botanical roses)	Classification	Botanical rose	Breeder Family	Origin	Ploidy	Ploidy source
a_feuilles_de_chanvre	Val de Marne rose garden	16	100.0	1810	A	no	Flobert	France	6	Bibliography
a_fleurs_rose_de_laffay	Val de Marne rose garden	10	90.2	1835	HSem	no	Laffay	FranceParis	2	Bibliography
a_longs_pedoncules	La Cour de Commer rose garden	15	100.0	1854	M	no	Robert	FranceAngers	4	Bibliography
abbaye_de_cluny	Val de Marne rose garden	8	50.1	1993	HT	no	Meilland	FranceSE	4	Bibliography
abbaye_de_valsaintes	Grande roseraie de Lyon	9	46.5	2006	HT	no	Dorieux	FranceLyon	4	Bibliography
abbe_duchemin	La Cour de Commer rose garden	2	96.4	Unknown	Unknown	no	Unknown	Unknown	Unknown	Unknown
abdul_hamid	Loubert rose garden	10	98.6	1901	HRg	no	Roseraie_de_l'Haÿ-Les-Roses	Unknown	3	Measured
adagio	Val de Marne rose garden	8	74.9	1998	HT	no	Cocker	EuropeN	4	Bibliography
adam	Loubert rose garden	9	64.2	1838	T	no	Adam	FranceNW	2	Measured
adam_messerich	Loubert rose garden	6	69.2	1920	B	no	Lambert	EuropeW	4	Measured
adelaide_d_orleans	Loubert rose garden	10	96.0	1826	HSem	no	Jacques	FranceParis	2	Bibliography
adele	La Cour de Commer rose garden	2	91.1	1814	HGal	no	Descemet	FranceParis	4	Bibliography
adele_heu	La Cour de Commer rose garden	2	81.7	1816	HGal	no	Vibert	FranceParis	4	Bibliography
adele_pavie	La Cour de Commer rose garden	4	63.5	1857	M	no	Vibert	FranceAngers	4	Bibliography
adrienne_de_cardoville	La Cour de Commer rose garden	2	54.1	1865	B	no	Guillot	FranceLyon	4	Bibliography
aennchen_von_tharau	Loubert rose garden	5	83.5	1886	HMult	no	Geschwind	EuropeW	4	Measured
agar	La Cour de Commer rose garden	2	69.6	1843	HGal	no	Vibert	FranceAngers	4	Bibliography
agatha	Val de Marne rose garden	2	77.4	1817	HGal	no	Unknown	Unknown	3	Bibliography
agathe_fatime	La Cour de Commer rose garden	3	77.1	1815	HGal	no	Descemet	FranceParis	4	Bibliography
agathe_incarnata	La Beaujoire rose garden	16	100.0	1811	HGal	no	Miellez	FranceNW	Unknown	Unknown
aglaia	Val de Marne rose garden	11	52.8	1895	HMult	no	Schmitt	FranceNE	2	Bibliography
agnes	Val de Marne rose garden	13	100.0	1900	HRg	no	Saunders	America	2	Bibliography
aimable_amie	Val de Marne rose garden	2	86.2	1805	HGal	no	Unknown	EuropeW	4	Bibliography
aimable_rouge	La Cour de Commer rose garden	15	100.0	1811	HGal	no	Vibert	FranceParis	4	Bibliography
aimee_vibert	Loubert rose garden	10	88.2	1828	N	no	Vibert	FranceParis	2	Measured
alberic_barbier	Val de Marne rose garden	9	66.4	1900	HWich	no	Barbier	FranceOrleans	2	Bibliography
albert_la_blottais	Jumaju rose garden	6	45.9	1881	HP	no	Moreau_&_Robert	FranceAngers	Unknown	Unknown
alcime	Jumaju rose garden	2	82.5	1845	HGal	no	Vibert	FranceAngers	4	Bibliography
alector_cramoisi	La Cour de Commer rose garden	2	85.8	1811	HGal	no	Dupont	FranceParis	4	Bibliography
alexander_hill_gray	Grande roseraie de Lyon	9	70.3	1909	T	no	Dickson	EuropeN	2	Bibliography
alexandre_girault	Val de Marne rose garden	9	48.8	1907	HWich	no	Barbier	FranceOrleans	2	Bibliography
alexandre_tremouillet	Val de Marne rose garden	9	44.5	1901	HWich	no	Barbier	FranceOrleans	2	Bibliography
alfred_colomb	Loubert rose garden	6	52.7	1852	HP	no	Lacharme	FranceLyon	4	Measured
alice_hoffman	Désert rose garden	9	69.2	1897	Ch	no	Hoffmann	EuropeW	Unknown	Unknown
alice_leroi	La Cour de Commer rose garden	2	95.7	1842	M	no	Vibert	FranceAngers	4	Bibliography
alice_vena	La Cour de Commer rose garden	4	47.2	1800	HGal	no	Unknown	Unknown	Unknown	Unknown
alida_lovett	Val de Marne rose garden	8	70.9	1905	HWich	no	Van_Fleet	America	2	Bibliography
aline_rozey	Jumaju rose garden	7	42.3	1884	N	no	Schwartz	FranceLyon	4	Measured
alliance_franco_russe	Grande roseraie de Lyon	8	99.1	1899	T	no	Goinard	FranceAngers	2	Bibliography
alpenfee	Loubert rose garden	5	44.1	1890	HSet	no	Geschwind	EuropeW	4	Measured
alphonse_soupert	Grande roseraie de Lyon	5	51.6	1883	HP	no	Lacharme	FranceLyon	4	Bibliography
alupka	Jumaju rose garden	6	40.9	1830	N	no	Gartois	Asia	4	Measured
amateur_andre_fourcaud	Val de Marne rose garden	8	77.8	1903	HT	no	Puyravaud	FranceSW	4	Bibliography
amazone	Désert rose garden	9	70.4	1872	T	no	Ducher	FranceLyon	2	Bibliography
ambroise_pare	Val de Marne rose garden	2	93.8	1846	HGal	no	Vibert	FranceAngers	4	Bibliography
amelia	Loubert rose garden	2	91.8	1823	A	no	Vibert	FranceParis	4	Measured
amelia1	Loubert rose garden	2	90.0	1823	A	no	Vibert	FranceParis	4	Measured
amelie_de_mansfield	La Cour de Commer rose garden	2	91.7	1842	HGal	no	Unknown	Unknown	4	Bibliography
america	Jardin botanique de la Tête d'Or	8	92.3	1893	HRg	no	Paul	EuropeN	2	Bibliography
american_beauty	Loubert rose garden	6	48.8	1875	HP	no	Lédéchaux	FranceParis	4	Measured
ami_charmet	Val de Marne rose garden	6	63.8	1901	HP	no	Dubreuil	FranceLyon	4	Bibliography
amy_robsart	Loubert rose garden	14	99.9	1894	HEg	no	Penzance	EuropeN	6	Measured
anacreon	La Cour de Commer rose garden	2	79.0	1818	HGal	no	Vibert	FranceParis	4	Bibliography
anatole_de_montesquieu	Jumaju rose garden	10	97.4	1860	HSem	no	Van_Houtte	EuropeW	2	Bibliography
anderken_an_moritz_von_frohlich	Val de Marne rose garden	8	90.2	1904	HT	no	Hinner	EuropeW	4	Bibliography
andre_leroy_d_angers	Loubert rose garden	6	68.1	1866	HP	no	Trouillard	FranceAngers	4	Bibliography
angelique_quetier	La Cour de Commer rose garden	2	74.2	1839	M	no	Quetier	FranceParis	4	Bibliography
anna_benary	Val de Marne rose garden	10	83.6	1902	Unknown	no	Unknown	Unknown	Unknown	Unknown
anna_maria_de_montravel	Loubert rose garden	10	58.5	1879	Pol	no	Rambaux	FranceLyon	2	Measured
anna_olivier	Jardin botanique de la Tête d'Or	8	62.6	1872	T	no	Ducher	FranceLyon	2	Bibliography
anna_scharsach	Loubert rose garden	6	46.1	1890	HP	no	Geschwind	EuropeW	4	Measured
anthony_waterer	Loubert rose garden	10	99.2	1896	HRg	no	Waterer	EuropeN	3	Measured
antoine_ducher	Grande roseraie de Lyon	6	66.2	1866	HP	no	Ducher	FranceLyon	4	Bibliography
antoine_schurz	Loubert rose garden	5	59.9	1890	HP	no	Geschwind	EuropeW	4	Measured
antonia_d_ormois	Loubert rose garden	2	53.9	1835	HGal	no	Vibert	FranceParis	4	Measured
antonia_d_ormois1	Loubert rose garden	2	71.9	1835	HGal	no	Vibert	FranceParis	4	Measured
archiduc_charles	Loubert rose garden	9	72.5	1815	HGal	no	Descemet	FranceParis	3	Measured
archiduchesse_elizabeth	Jumaju rose garden	5	64.4	1881	HP	no	Moreau_&_Robert	FranceAngers	4	Bibliography
archiduchesse_elizabeth_d_autriche	Loubert rose garden	5	60.3	1881	HP	no	Moreau_&_Robert	FranceAngers	4	Bibliography
ards_rover	Loubert rose garden	9	37.1	1898	HP	no	Dickson	EuropeN	3	Measured
ards_rover1	Loubert rose garden	9	46.3	1898	HP	no	Dickson	EuropeN	3	Measured
aristide_(repartee)	Loubert rose garden	12	55.1	1867	HP	no	Trouillard	FranceAngers	3	Measured
aristobule	La Cour de Commer rose garden	2	90.1	1849	M	no	Foulard	FranceAngers	4	Bibliography
arlequin	La Cour de Commer rose garden	2	82.2	1841	HGal	no	Unknown	Unknown	4	Bibliography
arthur_young	La Cour de Commer rose garden	2	89.5	1863	M	no	Portemer	FranceParis	4	Bibliography
asepala	La Cour de Commer rose garden	2	89.4	1837	M	no	Foulard	FranceAngers	4	Bibliography
astronomia	Val de Marne rose garden	9	52.2	2006	S	no	Meilland	FranceSE	3	Bibliography
attraction	Val de Marne rose garden	6	50.5	1886	HT	no	Dubreuil	FranceLyon	4	Bibliography
aurelia_liffa	Loubert rose garden	4	69.7	1885	HSet	no	Geschwind	EuropeW	4	Measured
aviateur_bleriot	Val de Marne rose garden	9	57.9	1909	HWich	no	Fauque	FranceOrleans	2	Bibliography
avoca	Val de Marne rose garden	8	87.6	1907	HT	no	Dickson	EuropeN	4	Bibliography
ayrshire_queen	Loubert rose garden	10	52.4	1835	Ayr	no	Unknown	EuropeN	2	Measured
bacchante	Loubert rose garden	2	93.9	1811	HGal	no	Unknown	Unknown	4	Measured
banksiaeflora	Val de Marne rose garden	10	89.7	1837	HSem	no	Laffay	FranceParis	2	Bibliography
bardou_job	Loubert rose garden	8	54.9	1887	B	no	Nabonnand	FranceSE	3	Measured
barillet	La Cour de Commer rose garden	2	91.7	1850	M	no	Verdier	FranceParis	4	Bibliography
baron_de_bonstetten	Loubert rose garden	15	100.0	1872	HP	no	Liabaud	FranceLyon	4	Measured
baron_de_gossard	La Cour de Commer rose garden	2	67.8	1902	HP	no	Unknown	Unknown	4	Bibliography
baron_de_wassenaer	La Cour de Commer rose garden	2	91.7	1854	M	no	Verdier	FranceParis	4	Bibliography
baron_girod_de_l_ain	Loubert rose garden	15	100.0	1897	HP	no	Reverchon	France	4	Bibliography
baronne_adolphe_de_rothschild	Grande roseraie de Lyon	8	36.3	1868	HP	no	Pernet	FranceLyon	4	Bibliography
baronne_henriette_de_snoy	Loubert rose garden	9	79.1	1897	T	no	Bernaix	FranceLyon	2	Bibliography
baronne_prevost	Loubert rose garden	5	84.2	1842	HP	no	Desprez	FranceParis	4	Bibliography
beau_narcisse	Loubert rose garden	4	70.3	1828	HGal	no	Miellez	FranceNW	4	Bibliography
beaute_virginale	Jumaju rose garden	16	100.0	1815	D	no	Descemet	FranceParis	Unknown	Unknown
bellard	Val de Marne rose garden	2	84.8	1842	HGal	no	Unknown	Unknown	4	Bibliography
belle_de_baltimore	Loubert rose garden	10	66.2	1843	HSet	no	Feast	America	2	Measured
belle_de_crecy	Loubert rose garden	2	90.6	1828	HGal	no	Roeser/Hardy	FranceParis	4	Measured
belle_de_yebles	Val de Marne rose garden	2	92.2	1835	HGal	no	Desprez	FranceParis	4	Bibliography
belle_des_jardins	Val de Marne rose garden	2	94.9	1872	HGal	no	Guillot	FranceLyon	4	Bibliography
belle_helene	Loubert rose garden	2	95.3	1815	HGal	no	Descemet	FranceParis	4	Measured
belle_herminie	Loubert rose garden	2	94.2	1819	HGal	no	Coquerel	FranceNW	4	Measured
belle_isis	Loubert rose garden	2	87.9	1845	HGal	no	Parmentier	EuropeW	4	Measured
belle_lyonnaise	Désert rose garden	9	68.0	1870	T	no	Levet	FranceLyon	2	Bibliography
belle_poitevine	Jardin botanique de la Tête d'Or	8	79.2	1894	HRg	no	Bruant	FranceAngers	2	Bibliography
belle_rosine	Loubert rose garden	3	64.8	1829	HGal	no	Vibert	FranceParis	4	Bibliography
belle_vichyssoise	Loubert rose garden	9	51.3	1895	N	no	Moreau_&_Robert	FranceAngers	3	Measured
belle_villageoise	Val de Marne rose garden	3	60.0	1839	HGal	no	Vibert	FranceParis	4	Bibliography
belle_virginie	Loubert rose garden	2	86.9	1828	HGal	no	Jardin_fleuriste_de_Sèvres	FranceParis	4	Measured
belvedere	Loubert rose garden	10	88.5	1829	HSem	no	Jacques	FranceParis	2	Measured
bengale_cerise	Désert rose garden	9	38.4	1800	Ch	no	Unknown	China	Unknown	Unknown
bengale_ducher	Grande roseraie de Lyon	9	74.9	1869	Ch	no	Ducher	FranceLyon	Unknown	Unknown
bennet_seedling	Loubert rose garden	10	79.2	1840	Ayr	no	Bennett	EuropeN	2	Measured

berengere	Loubert rose garden	2	61.6	1849	M	no	Vibert	FranceAngers	4	Measured
berenice	La Cour de Commer rose garden	2	95.2	1818	HGal	no	Vibert	FranceParis	4	Bibliography
bicolore_incomparable	Loubert rose garden	3	51.5	1866	HP	no	Touvais	FranceParis	4	Bibliography
bijou_des_amateurs	Val de Marne rose garden	1	50.9	1837	HGal	no	Jacquemet-Bonnefont	FranceLyon	4	Bibliography
billard_et_barre	Jumaju rose garden	8	54.8	1898	T	no	Pernet-Ducher	FranceLyon	2	Bibliography
bizarre_triomphant	Val de Marne rose garden	2	63.0	1790	HGal	no	Unknown	EuropeW	4	Bibliography
black_baccara	INRA Angers	8	87.7	2000	HT	no	Meilland	FranceSE	4	Measured
blairii_n_2	La Beaujoire rose garden	4	58.4	1845	HCh	no	Blair	EuropeN	Unknown	Unknown
blanc_de_vibert	Loubert rose garden	3	68.5	1846	P	no	Vibert	FranceAngers	4	Measured
blanc_pur	Loubert rose garden	8	83.4	1827	Misc_OGR	no	Mauget	FranceOrleans	4	Measured
blanche_a_fleurs_pleines	Jardin botanique de la Tête d'Or	10	44.6	1815	HSem	no	Descemet	FranceParis	Unknown	Unknown
blanche_de_belgique	Loubert rose garden	16	100.0	1846	A	no	Unknown	EuropeW	6	Bibliography
blanche_fleur	La Cour de Commer rose garden	2	88.2	1835	C	no	Vibert	FranceParis	4	Bibliography
blanche_moreau	Loubert rose garden	2	89.6	1880	M	no	Moreau_&_Robert	FranceAngers	4	Bibliography
blanche_simon	La Cour de Commer rose garden	3	43.8	1863	M	no	Moreau_&_Robert	FranceAngers	4	Bibliography
bleu_magenta	Jardin botanique de la Tête d'Or	11	55.5	1933	HMult	no	Grandes_Roseraies_du_Val_de_Loire	FranceOrleans	2	Bibliography
blush	Val de Marne rose garden	2	93.4	1902	M	no	Hooker	EuropeN	4	Bibliography
blush_damask	Loubert rose garden	2	66.4	1760	D	no	Unknown	Unknown	4	Measured
blush_hip	Loubert rose garden	5	42.7	1834	A	no	Unknown	EuropeN	4	Measured
blush_noisette	Loubert rose garden	9	69.0	1814	N	no	Noisette	America	2	Measured
boccace	Loubert rose garden	4	65.1	1859	HP	no	Moreau_&_Robert	FranceAngers	4	Measured
bonanza	La Beaujoire rose garden	8	71.5	1982	S	no	Kordes	EuropeW	Unknown	Unknown
bonica	La Beaujoire rose garden	9	29.5	1982	F	no	Meilland	FranceSE	3	Bibliography
bossuet	Loubert rose garden	9	52.9	1836	HP	no	Vibert	FranceParis	4	Measured
botzaris	Val de Marne rose garden	2	92.6	1856	D	no	Robert	FranceAngers	4	Bibliography
bougainville	Jardin botanique de la Tête d'Or	12	99.6	1822	N	no	Cochet	FranceParis	2	Measured
boula_de_nanteuil	Loubert rose garden	2	92.5	1834	HGal	no	Roeser	FranceParis	4	Measured
bouquet_charmant	Val de Marne rose garden	16	100.0	1810	HGal	no	Unknown	EuropeW	4	Bibliography
bouquet_d_or	Val de Marne rose garden	9	50.9	1872	N	no	Ducher	FranceLyon	3	Measured
bouquet_d_or	Val de Marne rose garden	9	52.9	1872	N	no	Ducher	FranceLyon	3	Measured
bouquet_de_venus	Val de Marne rose garden	2	73.6	1814	HGal	no	Unknown	Unknown	4	Bibliography
bridesmaid	Désert rose garden	8	73.1	1893	T	no	Moore	America	2	Bibliography
broceliande	Grande roseraie de Lyon	8	74.9	2001	HT	no	Adam	FranceNW	4	Bibliography
burgundy_rose	Jumaju rose garden	15	100.0	1664	HGal	no	Unknown	France	4	Bibliography
cabbage_rose	Jumaju rose garden	2	75.8	1318	C	no	Unknown	EuropeW	4	Bibliography
camaieu	La Cour de Commer rose garden	2	93.2	1826	HGal	no	Gendron	FranceAngers	4	Bibliography
camelia_rose	Jumaju rose garden	9	61.4	1830	Ch	no	Prévost	FranceNW	Unknown	Unknown
capitaine_basroger	Loubert rose garden	5	51.7	1890	M	no	Moreau_&_Robert	FranceAngers	4	Measured
capitaine_sisolet	Jumaju rose garden	4	56.3	1843	B	no	Unknown	Unknown	Unknown	Unknown
capitaine_williams	Val de Marne rose garden	2	82.2	1843	HGal	no	Unknown	Unknown	4	Bibliography
caprice_de_meilland	Val de Marne rose garden	8	63.1	1999	HT	no	Meilland	FranceSE	4	Bibliography
captain_hayward	Jumaju rose garden	6	62.7	1893	HP	no	Bennett	EuropeN	4	Bibliography
captain_philip_green	Désert rose garden	8	46.4	1899	T	no	Nabonnand	FranceSE	2	Bibliography
capucine_bicolore	Grande roseraie de Lyon	13	100.0	1590	Sp	no	Unknown	Middle_East	4	Bibliography
cardinal_de_richelieu	Loubert rose garden	4	56.5	1840	HGal	no	Parmentier	EuropeW	3	Bibliography
carmen	Val de Marne rose garden	10	95.6	1888	HRg	no	Lambert	EuropeW	4	Bibliography
caroline_bank	Loubert rose garden	6	64.7	1889	HMult	no	Geschwind	EuropeW	4	Measured
caroline_de_sansal	Jumaju rose garden	4	64.1	1849	HP	no	Desprez	FranceParis	4	Bibliography
caroline_marniesse	Jumaju rose garden	9	71.2	1848	N	no	Roeser	FranceParis	3	Measured
catherine_de_wurtemberg	La Cour de Commer rose garden	2	88.6	1843	M	no	Robert	FranceAngers	4	Bibliography
catherine_ghislaine	Val de Marne rose garden	2	90.5	1880	D	no	Unknown	Unknown	Unknown	Unknown
catherine_mermet	Loubert rose garden	8	84.5	1870	T	no	Guillot	FranceLyon	2	Bibliography
catherine_seyton	Loubert rose garden	14	94.1	1895	HEg	no	Penzance	EuropeN	6	Measured
catherine_soupert	Grande roseraie de Lyon	5	86.9	1879	HP	no	Lacharme	FranceLyon	4	Bibliography
celestial	La Cour de Commer rose garden	2	91.2	1739	A	no	Unknown	EuropeW	6	Bibliography
celia	Grande roseraie de Lyon	8	76.3	1906	HT	no	Paul	EuropeN	4	Bibliography
celina_dubos	Val de Marne rose garden	2	89.0	1850	D	no	Dubos	France	4	Bibliography
celine	Loubert rose garden	6	59.1	1824	B	no	Laffay	FranceParis	4	Bibliography
celine_forestier	La Beaujoire rose garden	9	77.2	1842	N	no	Trouillard	FranceAngers	2	Measured
celsiana	Jardin botanique de la Tête d'Or	2	63.3	1732	D	no	Unknown	EuropeW	4	Bibliography
cent_feuilles_descemet	Jumaju rose garden	1	66.5	1814	C	no	Descemet	FranceParis	4	Bibliography
cesonie	La Cour de Commer rose garden	4	70.5	1848	M	no	Moreau_&_Robert	FranceAngers	4	Bibliography
champion_of_the_world	Loubert rose garden	15	100.0	1891	HP	no	Woodhouse	America	Unknown	Unknown
champneys_pink_cluster	Loubert rose garden	4	71.6	1811	N	no	Champneys	America	2	Measured
chapelain_d_arenberg	Val de Marne rose garden	5	65.7	1847	HGal	no	Parmentier	EuropeW	4	Bibliography
charles_de_legrady	Grande roseraie de Lyon	9	49.0	1884	T	no	Pernet-Ducher	FranceLyon	2	Bibliography
charles_lawson	Loubert rose garden	5	80.3	1853	B	no	Lawson	EuropeN	4	Measured
charles_metroz	Val de Marne rose garden	9	37.7	1900	Pol	no	Schwartz	FranceLyon	2	Bibliography
charles_quint	Loubert rose garden	2	86.3	1856	HGal	no	Moreau_&_Robert	FranceAngers	4	Measured
charleston_graveyard	Désert rose garden	10	28.2	Unknown	HCh	no	Unknown	America	Unknown	Unknown
chloris	Loubert rose garden	2	67.4	1814	A	no	Descemet	FranceParis	4	Measured
christine_wright	Val de Marne rose garden	8	95.3	1909	HWich	no	Hoopes	America	4	Bibliography
cibles	Loubert rose garden	12	87.8	1893	HRg	no	Kaufmann	EuropeW	2	Measured
cinderella	Val de Marne rose garden	6	63.7	1859	N	no	Page	America	4	Measured
claire_jacquier	La Cour de Commer rose garden	2	55.8	1887	N	no	Bernaix	FranceLyon	2	Measured
claudia_augusta	Loubert rose garden	10	43.4	1856	N	no	Damaizin	FranceLyon	4	Measured
clemence_marchix	Grande roseraie de Lyon	9	68.1	1900	T	no	Bernaix	FranceLyon	2	Bibliography
clemence_raoux	Jumaju rose garden	6	61.2	1869	HP	no	Moreau_&_Robert	FranceAngers	4	Bibliography
clemence_robert	La Cour de Commer rose garden	2	88.3	1863	M	no	Moreau_&_Robert	FranceAngers	4	Bibliography
clementine	Loubert rose garden	3	51.5	1818	HGal	no	Vibert	FranceParis	4	Measured
clio	Loubert rose garden	6	66.9	1892	HP	no	Paul	EuropeN	4	Measured
clotilde_soupert	Loubert rose garden	10	69.1	1889	Pol	no	Soupert_&_Notting	EuropeW	2	Measured
cocktail	Val de Marne rose garden	9	43.6	1957	S	no	Meilland	FranceSE	3	Bibliography
colibri	Val de Marne rose garden	10	46.9	1885	Pol	no	Lille	FranceLyon	2	Bibliography
columella	Jumaju rose garden	2	83.3	1860	HGal	no	Moreau_&_Robert	FranceAngers	4	Bibliography
commandant_beaurepaire	Loubert rose garden	5	68.4	1864	HP	no	Moreau_&_Robert	FranceAngers	4	Measured
common_moss	Jumaju rose garden	1	56.2	1700	M	no	Unknown	Unknown	4	Bibliography
comte_d_epremesnil	Jumaju rose garden	12	100.0	1881	HRg	no	Lecomte	FranceNW	Unknown	Unknown
comte_de_chambord	Val de Marne rose garden	4	74.0	1855	P	no	Moreau_&_Robert	FranceAngers	4	Bibliography
comtesse_d_oxford	Jumaju rose garden	6	63.4	1869	HP	no	Guillot	FranceLyon	4	Bibliography
comtesse_de_caserta	Loubert rose garden	9	50.0	1877	T	no	Nabonnand	FranceSE	2	Measured
comtesse_de_galard_bearn	Désert rose garden	9	78.8	1893	N	no	Bernaix	FranceLyon	2	Bibliography
comtesse_de_labarthe	Désert rose garden	9	51.2	1857	T	no	Bernède	FranceSW	2	Bibliography
comtesse_de_labarthe_cl	Désert rose garden	9	51.7	1900	T	no	Lewis	Oceania	2	Bibliography
comtesse_de_lacepede	Jumaju rose garden	2	88.3	1838	HGal	no	Adam	FranceNW	Unknown	Unknown
comtesse_de_leusse	SCRADH	8	43.6	1878	T	no	Nabonnand	FranceSE	2	Bibliography
comtesse_de_murinais	Loubert rose garden	2	78.1	1843	M	no	Vibert	FranceAngers	4	Measured
conrad_ferdinand_meyer	Loubert rose garden	9	44.5	1897	HRg	no	Müller	EuropeW	4	Measured
coquette_des_blanches	Jardin botanique de la Tête d'Or	4	65.8	1871	B	no	Lacharme	FranceLyon	4	Measured
coralie	La Cour de Commer rose garden	2	82.4	1828	M	no	Miellez	FranceNW	4	Bibliography
corallina	Grande roseraie de Lyon	9	48.8	1890	T	no	Paul	EuropeN	2	Bibliography
cornelie	Jardin botanique de la Tête d'Or	9	52.1	1858	N	no	Moreau_&_Robert	FranceAngers	2	Bibliography
coronation	Val de Marne rose garden	9	48.7	1911	HWich	no	Turner	EuropeN	2	Bibliography
coronet	Val de Marne rose garden	10	61.3	1912	Pol	no	Paul	EuropeN	2	Bibliography
corporal_johann_nagy	Loubert rose garden	4	72.5	1890	HSet	no	Geschwind	EuropeW	4	Measured
cosimo_ridolphi	La Cour de Commer rose garden	3	59.6	1842	HGal	no	Vibert	FranceAngers	4	Bibliography
coupe_d_hebe	Jardin botanique de la Tête d'Or	8	62.0	1833	B	no	Bizard	FranceAngers	4	Bibliography
cramoisi_eblouissant	Loubert rose garden	2	92.6	1829	Ch	no	Unknown	EuropeW	4	Measured
cramoisi_picote	Val de Marne rose garden	2	77.2	1837	HGal	no	Vibert	FranceParis	4	Bibliography
cramoisi_superieur	Loubert rose garden	9	62.1	1832	Ch	no	Coquerel	FranceNW	3	Measured
creme	Loubert rose garden	10	94.6	1894	S	no	Geschwind	EuropeW	2	Measured
crepuscule	Val de Marne rose garden	9	68.5	1904	N	no	Dubreuil	FranceLyon	2	Measured
crimson_globe	Loubert rose garden	4	52.9	1839	M	no	Paul	EuropeN	4	Measured
crimson_rambler	Jardin botanique de la Tête d'Or	10	91.4	1893	HMult	no	Unknown	Asia	2	Bibliography
cuisse_de_nymphe	La Cour de Commer rose garden	2	91.7	1400	A	no	Unknown	Middle_East	6	Bibliography
cuisse_de_nymphe_emue	Loubert rose garden	16	100.0	1800	A	no	Dumont_de_Courset	EuropeN	3	Measured
cumberland_belle	La Cour de Commer rose garden	8	90.9	1900	M	no	Dreer	America	4	Bibliography
cyclope	Val de Marne rose garden	7	27.5	1909	Pol	no	Dubreuil	FranceLyon	2	Bibliography
daniel_lacombe	Désert rose garden	7	34.8	1885	HMult	no	Allard	FranceAngers	2	Bibliography

dawson	Loubert rose garden	10	54.1	1888	HMult	no	Dawson	America	2	Measured
dawson1	Loubert rose garden	10	52.0	1888	HMult	no	Dawson	America	2	Measured
de_candolle	La Cour de Commer rose garden	2	91.7	1857	M	no	Portemer	FranceParis	4	Bibliography
decoration_de_geschwind	Loubert rose garden	5	72.8	1884	HMult	no	Geschwind	EuropeW	4	Measured
delille	Loubert rose garden	5	57.6	1852	M	no	Robert	FranceAngers	4	Measured
dembrowsky	Val de Marne rose garden	3	70.0	1849	HP	no	Vibert	FranceAngers	4	Bibliography
depute_debussy	Val de Marne rose garden	8	39.2	1902	HT	no	Buatois	FranceNE	4	Bibliography
deschamps	Jumaju rose garden	9	72.4	1877	N	no	Deschamp	France	4	Measured
desire_bergera	Val de Marne rose garden	10	31.9	1909	HWich	no	Barbier	FranceOrleans	2	Bibliography
desire_parmentier	Val de Marne rose garden	3	60.2	1841	HGal	no	Parmentier	EuropeW	4	Bibliography
desprez_a_fleur_jaune	La Beaujoire rose garden	9	63.8	1835	N	no	Desprez	FranceParis	2	Measured
deuil_de_paul_fontaine	Loubert rose garden	4	62.5	1873	M	no	Fontaine	FranceParis	4	Bibliography
devoniensis	Jumaju rose garden	8	90.3	1838	T	no	Foster	EuropeN	2	Bibliography
diabolo	Val de Marne rose garden	9	47.6	1909	HWich	no	Fauque	FranceOrleans	2	Bibliography
directeur_alphand	Jumaju rose garden	5	83.4	1883	HP	no	Lévêque	FranceParis	4	Bibliography
directeur_constant_bernard	Val de Marne rose garden	8	72.5	1886	HT	no	Soupert_&_Notting	EuropeW	4	Bibliography
dis-moi_qui_je_suis	La Cour de Commer rose garden	4	71.7	Unknown	HGal	no	Unknown	Unknown	4	Bibliography
docteur_baillon	Grande roseraie de Lyon	6	63.2	1878	HP	no	Margottin	FranceParis	4	Bibliography
docteur_grill	Désert rose garden	9	61.1	1884	T	no	Bonnaire	FranceLyon	2	Bibliography
docteur_marjolin	La Cour de Commer rose garden	3	43.9	1842	M	no	Moreau_&_Robert	FranceAngers	4	Bibliography
docteur_o_donel_browne	Val de Marne rose garden	6	42.5	1908	HT	no	Dickson	EuropeN	4	Bibliography
docteur_ricaud	Val de Marne rose garden	10	43.7	1906	Pol	no	Corboeuf	FranceOrleans	2	Bibliography
docteur_troendlin	Val de Marne rose garden	8	94.8	1904	HT	no	Kaiser	EuropeW	4	Bibliography
dometil_beccard	Val de Marne rose garden	2	64.3	1839	C	no	Vibert	FranceParis	4	Bibliography
dona_sol	Loubert rose garden	2	88.0	1842	HGal	no	Vibert	FranceAngers	4	Measured
donau	Val de Marne rose garden	10	39.2	1913	HWich	no	Praskac	EuropeW	2	Bibliography
dorothy_perkins	Val de Marne rose garden	10	83.8	1901	HWich	no	Jackson_&_Perkins	America	2	Bibliography
double_brique	Val de Marne rose garden	2	92.1	1842	HGal	no	Unknown	Unknown	4	Bibliography
double_delight	La Beaujoire rose garden	6	63.1	1977	HT	no	Swim	America	4	Bibliography
double_pink	Val de Marne rose garden	13	98.7	1819	HSpn	no	Unknown	EuropeN	4	Bibliography
double_white	Val de Marne rose garden	13	100.0	1808	HSpn	no	Unknown	EuropeN	4	Bibliography
duc_d_angouleme	La Cour de Commer rose garden	15	100.0	1821	C	no	Unknown	EuropeW	3	Bibliography
duc_d_angouleme	Loubert rose garden	4	60.1	1821	C	no	Unknown	EuropeW	3	Measured
duc_d_anjou	Loubert rose garden	2	89.1	1862	HP	no	Boyau	FranceAngers	4	Bibliography
duc_de_bordeaux	Val de Marne rose garden	2	88.8	1820	HGal	no	Vibert	FranceParis	4	Bibliography
duc_de_brabant	La Cour de Commer rose garden	2	93.5	1817	C	no	Verleeuwen	EuropeW	4	Bibliography
duc_de_cambridge	Loubert rose garden	4	55.8	1840	D	no	Laffay	FranceParis	4	Measured
duc_de_constantine	Jardin botanique de la Tête d'Or	10	72.4	1857	Ayr	no	Schmitt	FranceLyon	Unknown	Unknown
duc_de_crillon	Val de Marne rose garden	6	42.1	1860	B	no	Moreau_&_Robert	FranceAngers	4	Bibliography
duc_de_marlborough	Jumaju rose garden	15	97.2	1885	HP	no	Vibert	FranceAngers	4	Bibliography
duchess_of_sutherland	Val de Marne rose garden	8	91.5	1839	HT	no	Dickson	EuropeN	4	Bibliography
duchesse_d_abrantes	La Cour de Commer rose garden	2	88.8	1851	M	no	Robert	FranceAngers	4	Bibliography
duchesse_d_angoulême	La Cour de Commer rose garden	5	58.0	1826	HGal	no	Vibert	FranceParis	4	Bibliography
duchesse_d_istrie	La Cour de Commer rose garden	4	46.7	1855	M	no	Laffay	FranceParis	4	Bibliography
duchesse_de_berry	Loubert rose garden	2	81.8	1825	HGal	no	Vibert	FranceParis	4	Measured
duchesse_de_brabant	Loubert rose garden	9	51.5	1857	T	no	Bernède	FranceSW	2	Bibliography
duchesse_de_buccleugh	Val de Marne rose garden	5	57.0	1837	HGal	no	Vibert	FranceParis	4	Bibliography
duchesse_de_cambaceres	Jumaju rose garden	6	64.8	1854	HP	no	Fontaine	FranceParis	4	Bibliography
duchesse_de_grammont	Loubert rose garden	10	33.8	1838	N	no	Unknown	Unknown	2	Bibliography
duchesse_de_montebello	Loubert rose garden	2	77.4	1824	HGal	no	Laffay	FranceParis	4	Measured
duchesse_de_portland	Loubert rose garden	2	89.8	1799	P	no	Unknown	Unknown	4	Measured
duchesse_de_verneuil	Loubert rose garden	2	94.5	1856	M	no	Portemer	FranceParis	4	Measured
duke_of_connaught	Val de Marne rose garden	8	44.2	1879	HT	no	Bennett	EuropeN	4	Bibliography
duke_of_edinburgh	Jumaju rose garden	6	56.9	1868	HP	no	Paul	EuropeN	Unknown	Unknown
duke_of_york	Jumaju rose garden	9	76.8	1893	Ch	no	Paul	EuropeN	Unknown	Unknown
dumortier	Val de Marne rose garden	5	54.9	1843	HGal	no	Parmentier	EuropeW	4	Bibliography
dupuy_jamain	Loubert rose garden	6	65.2	1868	HP	no	Jamain	FranceParis	4	Bibliography
dutch_fork_china	Désert rose garden	9	49.7	Unknown	Ch	no	Unknown	America	Unknown	Unknown
e_veyrat_hermanos	Grande roseraie de Lyon	9	67.9	1895	T	no	Bernaix	FranceLyon	2	Bibliography
earl_of_eldon	Jumaju rose garden	8	82.2	1872	N	no	Copin	EuropeN	3	Measured
eclair	Jumaju rose garden	9	34.6	1883	HP	no	Lacharme	FranceLyon	4	Bibliography
edmee_et_roger	Val de Marne rose garden	8	85.2	1902	HT	no	Ketten	EuropeW	4	Bibliography
edmond_deshayes	Val de Marne rose garden	8	83.6	1902	HT	no	Bernaix	FranceLyon	4	Bibliography
edmond_proust	Val de Marne rose garden	9	60.4	1903	HWich	no	Barbier	FranceOrleans	2	Bibliography
el_areana	Jumaju rose garden	13	94.1	1912	HSpn	no	Geschwind	EuropeW	Unknown	Unknown
eleonore_berkeley	Val de Marne rose garden	10	97.5	1902	HMult	no	Unknown	EuropeN	2	Bibliography
elie_beauvillain_cl	Val de Marne rose garden	9	48.1	1887	T	no	Beauvillain	FranceSW	2	Bibliography
elina	La Beaujoire rose garden	8	77.0	1984	HT	no	Dickson	EuropeN	4	Bibliography
elisa_boelle	Jumaju rose garden	2	87.7	1869	HP	no	Guillot	FranceLyon	4	Bibliography
elisa_robichon	Val de Marne rose garden	11	65.8	1902	HWich	no	Barbier	FranceOrleans	2	Bibliography
elisabeth_brow	La Cour de Commer rose garden	4	69.9	1883	M	no	Stammler	Unknown	4	Bibliography
elisabeth_d_angleterre	Jardin botanique de la Tête d'Or	14	99.1	1817	Unknown	no	Dupont	FranceParis	5	Bibliography
elise_rovella	Val de Marne rose garden	2	76.6	1842	HGal	no	Unknown	Unknown	4	Bibliography
emera	INRA Angers	8	44.5	1988	S	no	Noak	EuropeW	3	Bibliography
emily_laxton	Jumaju rose garden	5	64.3	1876	HP	no	Laxton	EuropeN	4	Bibliography
emotion	Val de Marne rose garden	6	66.7	1862	B	no	Guillot	FranceLyon	4	Bibliography
empress_of_china	Val de Marne rose garden	9	73.9	1893	Ch	no	Jackson	America	2	Bibliography
enfant_de_france	Grande roseraie de Lyon	9	63.2	1857	HP	no	Lartay	FranceParis	4	Bibliography
erinnerung_an_brod	Jumaju rose garden	5	90.2	1886	HSet	no	Geschwind	EuropeW	Unknown	Unknown
erinnerung_an_schloss_scharfenstein	Loubert rose garden	6	44.4	1892	HT	no	Geschwind	EuropeW	4	Bibliography
ernest_dupre	Jumaju rose garden	6	57.3	1904	HP	no	Boutigny	FranceNW	4	Bibliography
ernest_morel	Loubert rose garden	6	50.6	1898	HP	no	Cochet	FranceParis	4	Measured
ernst_dorell	Loubert rose garden	4	56.1	1887	HMult	no	Geschwind	EuropeW	4	Measured
esther	Val de Marne rose garden	2	91.1	1819	HGal	no	Vibert	FranceParis	4	Bibliography
etienne_levet	Grande roseraie de Lyon	6	64.5	1871	HP	no	Levet	FranceLyon	4	Bibliography
etoile_de_lyon	Loubert rose garden	9	60.1	1881	T	no	Guillot	FranceLyon	2	Measured
etoile_de_portugal	Désert rose garden	9	45.7	1898	HG	no	Cayeux	EuropeS	Unknown	Unknown
eucharis	Val de Marne rose garden	2	94.7	1815	HGal	no	Descemet	FranceParis	4	Bibliography
eugene_de_beauharnais	Désert rose garden	6	65.9	1838	Ch	no	Hardy	FranceParis	Unknown	Unknown
eugene_furst	Loubert rose garden	6	52.5	1875	HP	no	Soupert_&_Notting	EuropeW	4	Measured
eugenie_chamusseau	Jumaju rose garden	3	56.9	1872	C	no	Verdier	FranceParis	4	Bibliography
eugenie_de_guinoisseau	La Cour de Commer rose garden	3	49.8	1846	M	no	Guinoisseau-Flon	FranceAngers	4	Bibliography
eugenius	Val de Marne rose garden	13	100.0	1848	HSpn	no	Unknown	Unknown	4	Bibliography
eulalie_lebrun	Val de Marne rose garden	2	89.9	1844	HGal	no	Vibert	FranceAngers	4	Bibliography
euphrosine	Loubert rose garden	10	80.0	1895	HMult	no	Schmitt	FranceLyon	2	Measured
eurydice	Loubert rose garden	16	89.4	1887	HSet	no	Geschwind	EuropeW	3	Bibliography
evergreen_gene	Désert rose garden	8	50.6	2007	HG	no	Viraraghavan	Asia	Unknown	Unknown
fabvier	Jumaju rose garden	9	54.5	1829	Ch	no	Laffay	FranceParis	3	Bibliography
fanny_essler	La Cour de Commer rose garden	2	65.8	1835	HGal	no	Vibert	FranceParis	4	Bibliography
fanny_pavetot	La Cour de Commer rose garden	5	71.1	1820	HGal	no	Unknown	Unknown	4	Bibliography
fatinitza	Loubert rose garden	9	47.3	1886	HMult	no	Geschwind	EuropeW	2	Measured
felicite_bohain	Loubert rose garden	2	81.2	1866	M	no	Unknown	France	4	Bibliography
felicite_parmentier	Loubert rose garden	16	100.0	1841	A	no	Parmentier	EuropeW	4	Measured
fellemberg	Jumaju rose garden	9	53.6	1835	Ch	no	Fellemberg	EuropeW	3	Measured
fen_zhang_lu	Désert rose garden	9	53.2	Unknown	Ch	no	Unknown	China	Unknown	Unknown
ferdinand_chaffolte	Jumaju rose garden	16	100.0	1878	HP	no	Pernet	FranceLyon	2	Bibliography
ferdinand_de_buck	Val de Marne rose garden	2	51.8	1842	HGal	no	Unknown	Unknown	4	Bibliography
ferdinand_de_lesseps	Private donations	8	67.2	1869	HP	no	Granger	FranceParis	4	Bibliography
ferdinand_roussel	Val de Marne rose garden	11	60.8	1902	HWich	no	Barbier	FranceOrleans	2	Bibliography
fimbriata	Loubert rose garden	10	95.9	1891	HRg	no	Morlet	FranceNE	3	Measured
fisher_et_holmes	Loubert rose garden	6	40.7	1865	HP	no	Verdier	FranceParis	4	Bibliography
fleur_bicolore	Private donations	12	79.2	1810	Unknown	no	Unknown	EuropeW	2	Bibliography
fleur_de_pelletier	Val de Marne rose garden	3	65.7	1832	HGal	no	Unknown	Unknown	4	Bibliography
flocon_de_neige	Loubert rose garden	10	49.6	1898	Ch	no	Lille	FranceLyon	2	Measured
flora	Jardin botanique de la Tête d'Or	10	68.7	1830	HSem	no	Jacques	FranceParis	2	Bibliography
flora_mac_ivor	Loubert rose garden	14	99.9	1830	HEg	no	Lord_Penzance	EuropeN	6	Measured
flore	Val de Marne rose garden	6	27.8	1829	HSem	no	Jacques	FranceParis	2	Bibliography
flush_o_dawn	Val de Marne rose garden	8	81.5	1900	HT	no	Walsh	America	4	Bibliography
foliacee	La Cour de Commer rose garden	2	88.9	1810	C	no	Unknown	EuropeW	4	Bibliography

fortune_s_five_color	Désert rose garden	10	74.8	1844	T	no	Fortune	EuropeN	2	Bibliography
fragrant_cloud	La Beaujoire rose garden	8	85.9	1967	HT	no	Tantau	EuropeW	4	Bibliography
francois_arago	Loubert rose garden	6	49.0	1858	HP	no	Trouillard	FranceAngers	4	Measured
francois_coppee	Jumaju rose garden	9	55.7	1895	HP	no	Ledéchaux	FranceParis	2	Bibliography
francois_de_salignac	La Cour de Commer rose garden	2	90.3	1854	M	no	Robert	FranceAngers	4	Bibliography
francois_guillot	Val de Marne rose garden	9	62.1	1905	HWich	no	Barbier	FranceOrleans	2	Bibliography
francois_juranville	Val de Marne rose garden	9	49.3	1906	HWich	no	Barbier	FranceOrleans	2	Bibliography
francois_levet	Loubert rose garden	5	87.0	1880	HP	no	Levet	FranceLyon	4	Measured
frau_helene_videnz	Val de Marne rose garden	10	88.6	1904	HMult	no	Lambert	EuropeW	2	Bibliography
frau_karl_druschki	Grande roseraie de Lyon	9	67.2	1901	HP	no	Lambert	EuropeW	4	Bibliography
fraulein_octavia_hesse	Val de Marne rose garden	9	52.7	1909	HWich	no	Hesse	EuropeW	2	Bibliography
frederic_ii_de_prusse	Loubert rose garden	4	66.5	1847	HCh	no	Verdier	FranceParis	3	Measured
friesia	La Beaujoire rose garden	9	55.5	1973	F	no	Kordes	EuropeW	4	Bibliography
fulgens_gallique	Loubert rose garden	4	63.7	1828	HGal	no	Vibert	FranceParis	4	Measured
g_nabonnand	Grande roseraie de Lyon	9	48.2	1888	T	no	Nabonnand	FranceSE	2	Bibliography
gabrielle_luizet	Loubert rose garden	6	71.2	1877	HP	no	Liabaud	FranceLyon	4	Measured
gardeniaeflora	Jardin botanique de la Tête d'Or	10	82.1	1900	HMult	no	Benary	EuropeW	2	Bibliography
gaspard_monge	Loubert rose garden	4	54.8	1854	C	no	Robert	FranceAngers	4	Measured
gazella	Val de Marne rose garden	3	52.3	1902	HGal	no	Unknown	Unknown	4	Bibliography
geant_des_batailles	Grande roseraie de Lyon	6	53.9	1846	HP	no	Nérard	FranceLyon	Unknown	Unknown
gelbe_dagmar_hastrup	Loubert rose garden	10	76.1	1897	HRg	no	Moore	America	2	Bibliography
general_barral	Jumaju rose garden	5	71.6	1867	HP	no	Granger	FranceParis	4	Bibliography
general_clerc	La Cour de Commer rose garden	9	58.7	1845	M	no	Portemer	FranceParis	4	Bibliography
general_donnadieu	Val de Marne rose garden	2	85.6	1842	HGal	no	Unknown	Unknown	4	Bibliography
general_drouot	La Cour de Commer rose garden	3	64.8	1845	M	no	Vibert	FranceAngers	4	Bibliography
general_gallieni	SCRADH	9	62.0	1899	T	no	Nabonnand	FranceSE	2	Bibliography
general_jacqueminot	Jumaju rose garden	6	59.9	1853	HP	no	Roussel	France	4	Bibliography
general_kleber	Loubert rose garden	2	83.7	1856	M	no	Robert	FranceAngers	4	Measured
general_schablikine	SCRADH	9	65.3	1878	T	no	Nabonnand	FranceSE	2	Bibliography
georges_cain	Jumaju rose garden	9	55.4	1909	HRg	no	Müller	EuropeW	2	Bibliography
georges_vibert	Val de Marne rose garden	2	89.7	1853	HGal	no	Robert	FranceAngers	4	Bibliography
gertrude	Val de Marne rose garden	8	79.3	1903	HT	no	Dickson	EuropeN	4	Bibliography
geschwind_s_nordland_n°_1	Loubert rose garden	5	82.8	1884	HSet	no	Geschwind	EuropeW	4	Measured
geschwind_s_orden	Loubert rose garden	4	76.3	1886	HMult	no	Geschwind	EuropeW	4	Measured
geschwind_s_schonstee	Loubert rose garden	9	40.2	1900	HMult	no	Geschwind	EuropeW	3	Measured
gilda	Loubert rose garden	4	69.1	1887	HMult	no	Geschwind	EuropeW	2	Bibliography
gloire_d_orient	La Cour de Commer rose garden	2	79.7	1855	M	no	Béluze	FranceLyon	4	Bibliography
gloire_de_dijon	Val de Marne rose garden	8	80.2	1850	T	no	Jacotot	FranceNE	Unknown	Unknown
gloire_de_dijon1	Val de Marne rose garden	9	72.4	1850	T	no	Jacotot	FranceNE	Unknown	Unknown
gloire_de_ducher	Val de Marne rose garden	5	44.9	1865	HP	no	Ducher	FranceLyon	4	Bibliography
gloire_des_mousseux	Loubert rose garden	5	55.2	1852	M	no	Laffay	FranceParis	4	Measured
gloire_des_polyanthas	Jumaju rose garden	9	41.4	1887	Pol	no	Guillot	FranceLyon	Unknown	Unknown
gloire_des_rosomanes	Loubert rose garden	10	54.6	1825	Ch	no	Vibert	FranceParis	4	Measured
gloire_lyonnaise	Loubert rose garden	9	53.7	1884	HP	no	Guillot	FranceLyon	3	Measured
glory_of_waltham	Jumaju rose garden	6	43.3	1865	HP	no	Vigneron	FranceAngers	4	Bibliography
goethe	La Cour de Commer rose garden	15	100.0	1911	M	no	Lambert	EuropeW	3	Bibliography
gourdault	Val de Marne rose garden	8	52.9	1859	B	no	Guillot	FranceLyon	4	Bibliography
grace_molyneux	Val de Marne rose garden	8	72.4	1909	HT	no	Dickson	EuropeN	4	Bibliography
gracilis	La Cour de Commer rose garden	2	83.9	1820	A	no	Calvert	FranceNW	6	Bibliography
graham_thomas	Val de Marne rose garden	8	37.4	1983	S	no	Austin	EuropeN	4	Bibliography
grand_cramoisi	La Cour de Commer rose garden	2	85.0	1818	HGal	no	Vibert	FranceParis	4	Bibliography
grand_duc_alexis	Jumaju rose garden	6	68.8	1892	HP	no	Lévêque	FranceParis	4	Bibliography
grande_cramoisi	Val de Marne rose garden	16	100.0	1818	HGal	no	Vibert	FranceParis	4	Bibliography
grande_renoncule	Val de Marne rose garden	3	62.5	1842	HGal	no	Unknown	Unknown	4	Bibliography
green_mantle	Jardin botanique de la Tête d'Or	14	97.2	1895	HEg	no	Penzance	EuropeN	5	Bibliography
gribaldo_nicola	Jumaju rose garden	6	62.4	1890	T	no	Soupert_&_Notting	EuropeW	2	Bibliography
gruss_an_teplitz	Loubert rose garden	6	62.1	1897	HCh	no	Geschwind	EuropeW	4	Bibliography
haddington	Loubert rose garden	2	68.4	1828	HGal	no	Unknown	Unknown	Unknown	Unknown
hansa	Val de Marne rose garden	12	100.0	1905	HRg	no	Schaum/Van_Tol	EuropeW	2	Bibliography
hector	Val de Marne rose garden	5	69.5	1828	HGal	no	Parmentier	EuropeW	4	Bibliography
helene_granger	Jardin botanique de la Tête d'Or	9	72.0	1910	HMult	no	Granger	FranceParis	Unknown	Unknown
helga_s_dream	Flower Research Institute, China	10	99.5	Unknown	HMult	no	Unknown	China	Unknown	Unknown
henri_4	Loubert rose garden	6	44.3	1862	HP	no	Verdier	FranceParis	4	Bibliography
henri_foucquier	La Cour de Commer rose garden	2	95.3	1842	HGal	no	Unknown	Unknown	4	Bibliography
henri_martin	Val de Marne rose garden	2	67.9	1862	M	no	Laffay	FranceParis	4	Bibliography
henriette	La Cour de Commer rose garden	2	88.2	1811	HGal	no	Unknown	America	4	Bibliography
herodiade	Jardin botanique de la Tête d'Or	9	66.7	1888	N	no	Brassac	FranceSW	2	Bibliography
hiawatha	Val de Marne rose garden	9	30.3	1904	HMult	no	Walsh	America	2	Bibliography
himmelsauge	Loubert rose garden	3	54.0	1893	HSet	no	Geschwind	EuropeW	4	Measured
hippolyte	Loubert rose garden	4	62.7	1832	HGal	no	Parmentier	EuropeW	3	Measured
homere	Désert rose garden	9	63.2	1858	T	no	Moreau_&_Robert	FranceAngers	3	Bibliography
horace_vernet	Loubert rose garden	2	78.3	1866	HP	no	Guillot	FranceLyon	4	Measured
horace_vernet	Loubert rose garden	2	82.8	1866	HP	no	Guillot	FranceLyon	4	Measured
hortense_vernet	La Cour de Commer rose garden	2	86.3	1861	M	no	Moreau_&_Robert	FranceAngers	4	Bibliography
hovyn_de_tronchere	Loubert rose garden	9	43.7	1899	T	no	Puyravaud	FranceSW	2	Bibliography
hume_s_blush_tea_scented_china	Loubert rose garden	9	61.4	1809	T	no	Unknown	China	2	Measured
huntington_la_biche	Désert rose garden	9	70.0	1851	T	no	Robert	FranceAngers	2	Bibliography
imperatrice_eugenie	Val de Marne rose garden	5	60.0	1854	HP	no	Oger	FranceNW	4	Bibliography
imperatrice_josephine	Loubert rose garden	2	91.1	1815	HGal	no	Descemet	FranceParis	4	Bibliography
ina_bingham	Val de Marne rose garden	8	96.9	1906	HP	no	Dickson	EuropeN	4	Bibliography
incomparable_d_auteuil	Val de Marne rose garden	3	85.1	1828	C	no	Laffay	FranceParis	4	Bibliography
inconnu_av_juliette_adam	SCRADH	9	46.9	Unknown	Unknown	no	Unknown	Unknown	Unknown	Unknown
inconnu_golf_juan	SCRADH	9	65.3	Unknown	Unknown	no	Unknown	Unknown	Unknown	Unknown
inconnu_saint_tropez	SCRADH	9	60.8	Unknown	Unknown	no	Unknown	Unknown	Unknown	Unknown
indigo	Loubert rose garden	2	85.1	1830	P	no	Laffay	FranceParis	4	Measured
ingrid_bergman	La Beaujoire rose garden	9	58.9	1985	HT	no	Poulsen	EuropeN	4	Bibliography
irene_bonnet	SCRADH	8	68.6	1920	HT	no	Nabonnand	FranceSE	4	Bibliography
irene_watts	Désert rose garden	8	79.2	1895	Ch	no	Guillot	FranceLyon	Unknown	Unknown
isabelle_nabonnand	SCRADH	9	75.3	1873	T	no	Nabonnand	FranceSE	2	Bibliography
ispahan	Loubert rose garden	3	69.2	1827	D	no	Unknown	Middle_East	4	Measured
jacques_amyot	Désert rose garden	6	49.6	1849	N	no	Varangot	FranceParis	Unknown	Unknown
jacques_cartier	Val de Marne rose garden	4	51.3	1868	P	no	Desprez	FranceParis	4	Bibliography
jacques_cartier_blanc	Loubert rose garden	5	80.7	1868	P	no	Pedersen	EuropeN	4	Bibliography
james_bourgault	Jumaju rose garden	5	66.5	1864	HP	no	Verdier	FranceParis	4	Bibliography
james_mitchel	Loubert rose garden	3	51.7	1861	M	no	Verdier	FranceParis	4	Measured
james_veitch	La Cour de Commer rose garden	16	100.0	1864	M	no	Verdier	FranceParis	4	Bibliography
janet_s_pride	Loubert rose garden	3	52.8	1894	HEg	no	Descemet	FranceParis	4	Measured
jean_andre	Jardin botanique de la Tête d'Or	9	68.3	1893	T	no	Pelletier	FranceParis	2	Bibliography
jean_bach_sisley	Loubert rose garden	9	36.4	1898	Ch	no	Dubreuil	FranceLyon	2	Measured
jean_bodin	La Cour de Commer rose garden	10	96.4	1846	M	no	Vibert	FranceAngers	4	Bibliography
jean_ducher	Loubert rose garden	8	42.7	1874	T	no	Ducher	FranceLyon	2	Bibliography
jean_girin	Val de Marne rose garden	9	44.2	1910	HWich	no	Girin	FranceLyon	2	Bibliography
jean_lorthois	Val de Marne rose garden	9	47.1	1879	HT	no	Ducher	FranceLyon	4	Bibliography
jeanne_d_arc	Loubert rose garden	10	94.2	1818	A	no	Vibert	FranceParis	2	Measured
jeanne_d_arc_alba	Loubert rose garden	16	100.0	1818	A	no	Vibert	FranceParis	6	Measured
jeanne_drivon	Grande roseraie de Lyon	6	60.1	1883	Pol	no	Schwartz	FranceLyon	Unknown	Unknown
jeanne_hachette	La Cour de Commer rose garden	2	86.4	1842	HGal	no	Vibert	FranceAngers	4	Bibliography
jenny_duval	Loubert rose garden	2	91.8	1842	HGal	no	Duval	FranceParis	4	Measured
jersey_beauty	Loubert rose garden	9	57.7	1899	HWich	no	Horvath	America	2	Bibliography
jeune_henry	Jumaju rose garden	8	44.2	1815	P	no	Descemet	FranceParis	4	Bibliography
jiminy_cricket	Jumaju rose garden	5	65.3	1954	F	no	Boemer	America	3	Bibliography
john_cranston	La Cour de Commer rose garden	2	82.3	1861	M	no	Verdier	FranceParis	4	Bibliography
john_hopper	Jumaju rose garden	8	81.8	1862	HP	no	Ward	EuropeN	4	Bibliography
john_ruskin	Val de Marne rose garden	8	76.6	1903	HT	no	Dickson	EuropeN	4	Bibliography
josephine_de_beauharnais	Loubert rose garden	4	72.1	1865	HP	no	Guillot	FranceLyon	4	Measured
josephine_ritter	Loubert rose garden	5	67.9	1900	HMult	no	Geschwind	EuropeW	2	Bibliography
jules_gravereaux	Loubert rose garden	9	48.4	1908	Unknown	no	Pernet-Ducher	FranceLyon	Unknown	Unknown
jules_margottin	Loubert rose garden	5	91.5	1852	HP	no	Margottin	FranceParis	4	Measured
jules_toussaint	Val de Marne rose garden	8	76.2	1900	HT	no	Bonnaire	FranceLyon	4	Bibliography

julie_d_estanges	Val de Marne rose garden	3	45.2	1834	HGal	no	Vibert	FranceParis	4	Bibliography
julie_de_mersan	Loubert rose garden	2	54.4	1854	M	no	Thomas	FranceNW	4	Measured
julie_de_mersan	Loubert rose garden	2	47.5	1854	M	no	Thomas	FranceNW	4	Measured
julie_krudner	Val de Marne rose garden	2	88.5	1847	P	no	Laffay	FranceParis	4	Bibliography
juliette	Val de Marne rose garden	2	92.8	1828	HGal	no	Miellez	FranceNW	4	Bibliography
just_joey	La Beaujoire rose garden	8	91.1	1972	HT	no	Roger_Pawsey_Of_Cants_Of_Colchester	EuropeN	4	Bibliography
justine_ramet	La Cour de Commer rose garden	2	85.8	1845	C	no	Vibert	FranceAngers	4	Bibliography
katharina_zeimet	Val de Marne rose garden	10	95.2	1901	Pol	no	Lambert	EuropeW	2	Bibliography
kazanlik	Jardin botanique de la Tête d'Or	4	48.2	1612	D	no	Unknown	Middle_East	4	Bibliography
kean	La Cour de Commer rose garden	2	70.2	1843	HGal	no	Laffay	FranceParis	4	Bibliography
kiese	Val de Marne rose garden	15	99.9	1910	S	no	Kiese	EuropeW	5	Bibliography
killarney	Loubert rose garden	6	43.3	1898	HT	no	Dickson	EuropeN	3	Measured
king_of_siam	Désert rose garden	8	31.7	1825	HT	no	Laffay	FranceParis	Unknown	Unknown
knock_out	INRA Angers	9	35.6	1988	S	no	Radler	America	3	Bibliography
Konigin_von_Danemark	La Cour de Commer rose garden	6	67.1	1816	A	no	Booth	EuropeW	Unknown	Unknown
kronprinzessin_viktoria	Val de Marne rose garden	8	88.9	1888	B	no	Volvert	Unknown	4	Bibliography
l_enchanteresse	Val de Marne rose garden	2	69.5	1826	HGal	no	Francois	EuropeW	4	Bibliography
l_esperance	Val de Marne rose garden	5	79.6	1871	HP	no	Lartay	FranceParis	4	Bibliography
l_eveque	Jumaju rose garden	4	51.5	1790	HGal	no	Volvert	France	4	Bibliography
l_invincible	La Cour de Commer rose garden	2	88.5	1819	HGal	no	Miellez	FranceNW	4	Bibliography
l_ouche	Loubert rose garden	6	70.4	1901	HCh	no	Buatois	FranceNE	4	Measured
l19_laos	Désert rose garden	10	31.9	2008	Ch	no	Maebara	China	Unknown	Unknown
la_belle_distinguee	Loubert rose garden	2	56.7	1811	HEg	no	Unknown	Unknown	4	Measured
la_belle_sultane	La Cour de Commer rose garden	2	92.2	1795	HGal	no	Unknown	EuropeW	4	Bibliography
la_biche	Loubert rose garden	9	61.5	1832	N	no	Toullier	FranceParis	2	Measured
la_caille	Loubert rose garden	4	63.8	1857	M	no	Moreau_&_Robert	FranceAngers	4	Bibliography
la_favorite	Loubert rose garden	8	80.2	1899	HT	no	Schwartz	FranceLyon	3	Measured
la_france	Loubert rose garden	8	96.0	1867	HT	no	Guillot	FranceLyon	3	Measured
la_france_cl	Val de Marne rose garden	8	96.3	1893	HT	no	Henderson	America	4	Bibliography
la_gloire_des_jardins	La Cour de Commer rose garden	2	92.6	1815	HGal	no	Descemet	FranceParis	4	Bibliography
la_louise	Jumaju rose garden	2	88.8	1840	HGal	no	Parmentier	EuropeW	4	Bibliography
la_nationale	La Cour de Commer rose garden	2	80.7	1836	HGal	no	Unknown	Unknown	4	Bibliography
la_negresse	Loubert rose garden	4	61.8	1842	D	no	Vibert	FranceAngers	3	Measured
la_noblesse	Loubert rose garden	5	58.0	1857	C	no	Soupert_&_Notting	EuropeW	4	Measured
la_noblesse	Loubert rose garden	5	74.6	1857	C	no	Soupert_&_Notting	EuropeW	4	Measured
la_perle	Val de Marne rose garden	11	52.2	1904	HWich	no	Fauque	FranceOrleans	2	Bibliography
la_pucelle	Val de Marne rose garden	4	68.7	1811	HGal	no	Dubourg	FranceParis	4	Bibliography
la_reine	Grande roseraie de Lyon	9	71.5	1842	HP	no	Laffay	FranceParis	4	Bibliography
la_revenante	Val de Marne rose garden	2	98.3	1825	HGal	no	Miellez	FranceNW	4	Bibliography
la_rose_d_york	Jardin botanique de la Tête d'Or	16	100.0	1629	A	no	Unknown	Europe	6	Bibliography
la_rubanee	Loubert rose garden	14	100.0	1839	HGal	no	Vibert	FranceParis	4	Measured
la_sevillana	La Beaujoire rose garden	8	80.5	1978	F	no	Meilland	FranceSE	3	Bibliography
la_sirene	Loubert rose garden	6	42.5	1875	HP	no	Soupert_&_Notting	EuropeW	4	Measured
la_tosca	Grande roseraie de Lyon	8	84.2	1900	HT	no	Schwartz	FranceLyon	4	Bibliography
la_vierzonnaise	Val de Marne rose garden	5	86.3	1893	HP	no	Andre	FranceParis	4	Bibliography
la_virginale	Jumaju rose garden	4	72.7	1400	A	no	Unknown	Middle_East	6	Bibliography
lady_dunmore	Val de Marne rose garden	13	99.1	1906	HSpn	no	Unknown	EuropeN	4	Bibliography
lady_edine	Val de Marne rose garden	13	99.5	1906	HSpn	no	Unknown	EuropeN	4	Bibliography
lady_emily_peel	Jumaju rose garden	6	45.5	1862	N	no	Lacharme	FranceLyon	4	Measured
lady_mary_corry	Loubert rose garden	15	100.0	1901	T	no	Dickson	EuropeN	5	Measured
lady_penzance	Désert rose garden	14	100.0	1894	HEg	no	Penzance	EuropeN	6	Bibliography
lady_waterlow	SCRADH	8	49.3	1902	HT	no	Nabonnand	FranceSE	4	Bibliography
lamotte_sanguin	Loubert rose garden	5	67.9	1869	HP	no	Vigneron	FranceAngers	4	Measured
lane	La Cour de Commer rose garden	3	52.0	1845	M	no	Robert	FranceAngers	4	Bibliography
laneii	Jumaju rose garden	3	62.1	1845	M	no	Laffay	FranceParis	4	Bibliography
las_casas	Loubert rose garden	6	65.3	1828	B	no	Vibert	FranceParis	Unknown	Unknown
laurent_heister	Val de Marne rose garden	2	75.7	1859	P	no	Moreau_&_Robert	FranceAngers	4	Bibliography
le_cid	Val de Marne rose garden	12	95.2	1909	HRg	no	Vigneron	FranceAngers	2	Bibliography
le_loberde	La Cour de Commer rose garden	2	90.1	1800	M	no	Unknown	France	4	Bibliography
le_pactole	Loubert rose garden	9	48.2	1841	T	no	Miellez	FranceNW	2	Bibliography
le_poilu	Jumaju rose garden	2	86.0	1915	HWich	no	Barbier	FranceOrleans	4	Bibliography
le_rire_niais	La Cour de Commer rose garden	15	100.0	1810	C	no	Dupont	FranceParis	4	Bibliography
lea	Loubert rose garden	2	78.1	1825	HGal	no	Vetillard	FranceAngers	4	Measured
leda	Loubert rose garden	2	85.1	1827	D	no	Unknown	EuropeN	4	Measured
lena_turner	Jumaju rose garden	6	34.6	1869	HP	no	Verdier	FranceParis	4	Bibliography
leon_robichon	Val de Marne rose garden	8	58.8	1902	HP	no	Robichon	FranceOrleans	4	Bibliography
leonardo_de_vinci	La Beaujoire rose garden	8	60.7	1994	F	no	Meilland	FranceSE	3	Bibliography
leonie_lamesch	Jumaju rose garden	9	35.2	1900	Pol	no	Lambert	EuropeW	Unknown	Unknown
leontine_gervais	Val de Marne rose garden	9	67.4	1903	HWich	no	Barbier	FranceOrleans	2	Bibliography
leopold_ritter	Loubert rose garden	6	60.2	1899	HMult	no	Geschwind	EuropeW	4	Measured
les_saisons_d_italie	La Cour de Commer rose garden	15	100.0	Unknown	HGal	no	Unknown	Unknown	4	Bibliography
leuchtstern	Loubert rose garden	10	99.6	1889	HMult	no	Kiese	EuropeW	2	Measured
ley_s_perpetual	Désert rose garden	8	40.4	1863	HT	no	Damaizan	EuropeN	2	Bibliography
li_jiang_yellow	Désert rose garden	10	86.4	Unknown	T	no	Maebara	China	2	Bibliography
lieutenant_chaure	Val de Marne rose garden	8	74.3	1909	HT	no	Pernet-Ducher	FranceLyon	4	Bibliography
lijiang_rose	Désert rose garden	10	86.6	1995	HG	no	Osti	China	Unknown	Unknown
lijiang_yellow_tea	Désert rose garden	10	96.0	1995	T	no	Maebara	China	Unknown	Unknown
lily_mertschersky	Jumaju rose garden	6	66.2	1878	N	no	Nabonnand	FranceSE	2	Bibliography
lisette_de_beranger	Val de Marne rose garden	6	62.1	1867	HP	no	Moreau	FranceAngers	4	Bibliography
lord_penzance	Loubert rose garden	14	100.0	1894	HEg	no	Penzance	EuropeN	5	Measured
lord_raglan	Jumaju rose garden	8	79.2	1854	HP	no	Guillot	FranceLyon	4	Bibliography
louis_de_funes	La Beaujoire rose garden	9	36.7	1987	HT	no	Meilland	FranceSE	4	Bibliography
louis_lille	Loubert rose garden	2	90.9	1887	HP	no	Dubreuil	FranceLyon	4	Bibliography
louis_noisette	Val de Marne rose garden	8	62.0	1864	HP	no	Ducher	FranceLyon	4	Bibliography
louis_van_tyll	Val de Marne rose garden	2	90.9	1846	HGal	no	Unknown	EuropeW	4	Bibliography
louise_catherine_breslau_cl	Val de Marne rose garden	4	65.1	1917	HT	no	Kordes	EuropeW	Unknown	Unknown
louise_d_arzens	Loubert rose garden	8	42.0	1861	N	no	Lacharme	FranceLyon	4	Measured
louise_odier	Jardin botanique de la Tête d'Or	6	64.9	1851	B	no	Margottin	FranceParis	4	Bibliography
louise_verger	La Cour de Commer rose garden	8	62.1	1860	M	no	Moreau_&_Robert	FranceAngers	4	Bibliography
lucie_duplessis	La Cour de Commer rose garden	2	91.7	1854	M	no	Robert	FranceAngers	4	Bibliography
lucien_de_lemos	Val de Marne rose garden	8	93.1	1905	HT	no	Lambert	EuropeW	4	Bibliography
lucy_bertram	Val de Marne rose garden	14	100.0	1895	HEg	no	Penzance	EuropeN	5	Bibliography
lycoris	Val de Marne rose garden	2	80.3	1835	HGal	no	Vibert	FranceParis	Unknown	Unknown
m_a_maille	Val de Marne rose garden	6	64.9	1889	B	no	Robert	FranceAngers	4	Bibliography
m_cordeau	Loubert rose garden	8	94.0	1892	B	no	Moreau_&_Robert	FranceAngers	4	Bibliography
m_de_montigny	Val de Marne rose garden	5	84.3	1855	HP	no	Paillet	FranceParis	4	Bibliography
m_francisque_rives	Val de Marne rose garden	8	54.3	1883	HP	no	Schwartz	FranceLyon	4	Bibliography
m_helye	Val de Marne rose garden	10	89.3	1889	HRg	no	Morlet	FranceNE	2	Bibliography
m_louis_ricard	Val de Marne rose garden	6	47.8	1894	HP	no	Boutigny	FranceNW	4	Bibliography
m_tillier	Désert rose garden	9	55.5	1891	T	no	Bernaix	FranceLyon	2	Bibliography
ma_tulipe	Grande roseraie de Lyon	9	65.1	1899	HT	no	Bonnaire	FranceLyon	4	Bibliography
mabel_morrison	Val de Marne rose garden	6	51.8	1878	HP	no	Broughton	EuropeN	4	Bibliography
magna_charta	Loubert rose garden	5	90.2	1876	HP	no	Paul	EuropeN	4	Measured
maiden_s_blush_small	Val de Marne rose garden	4	71.8	1797	A	no	Kew	EuropeN	6	Bibliography
maitland_white	Désert rose garden	10	94.8	Unknown	T	no	Unknown	America	2	Bibliography
malesherbes	La Cour de Commer rose garden	2	94.0	1836	HGal	no	Vibert	FranceParis	Unknown	Unknown
malvina	Loubert rose garden	2	76.7	1841	C	no	Verdier/Vibert	FranceParis	4	Bibliography
manteau_pourpre	Loubert rose garden	2	92.2	1810	HGal	no	Vibert	FranceParis	4	Bibliography
marbree	Loubert rose garden	2	62.8	1858	P	no	Moreau_&_Robert	FranceAngers	4	Bibliography
marcel_bourgoin	Val de Marne rose garden	12	100.0	1899	HGal	no	Corboeuf	FranceOrleans	4	Bibliography
marchionnes_of_salisburry	Loubert rose garden	6	61.8	1890	HT	no	Pernet	FranceLyon	4	Bibliography
marechal_niel	Val de Marne rose garden	9	69.0	1864	N	no	Castel	France	2	Bibliography
margaret_haywood	Val de Marne rose garden	5	73.4	1889	HP	no	Haywood	EuropeN	4	Bibliography
marguerite_de_roman	Loubert rose garden	5	82.5	1882	HP	no	Schwartz	FranceLyon	4	Measured
marguerite_desrayaux	Jardin botanique de la Tête d'Or	9	45.9	1906	N	no	Nabonnand	FranceSE	Unknown	Unknown
marie_accary	Désert rose garden	6	56.9	1872	N	no	Guillot	FranceLyon	3	Measured
marie_baumann	Jumaju rose garden	6	38.3	1863	HP	no	Baumann	FranceNE	Unknown	Unknown
marie_boissee	Val de Marne rose garden	4	51.6	1864	HP	no	Oger	FranceNW	4	Bibliography
marie_de_saint_jean	Val de Marne rose garden	2	79.0	1869	P	no	Damaizin	FranceLyon	4	Bibliography

marie_dermar	Loubert rose garden	6	62.6	1889	N	no	Geschwind	EuropeW	4	Measured
marie_dermar	Loubert rose garden	10	90.3	1889	N	no	Geschwind	EuropeW	4	Measured
marie_ducher	Private donations	9	45.9	1868	T	no	Ducher	FranceLyon	2	Bibliography
marie_leczinska	La Cour de Commer rose garden	3	85.3	1860	M	no	Béluze	FranceLyon	4	Bibliography
marie_pavie	Jumaju rose garden	7	32.9	1888	Pol	no	Alégatière	FranceLyon	2	Bibliography
marie_robert	Val de Marne rose garden	9	57.8	1893	N	no	Cochet	FranceParis	2	Measured
marie_therese_dubourg	Jumaju rose garden	5	46.8	1888	N	no	Godard	FranceLyon	4	Measured
marie-louise	La Cour de Commer rose garden	1	62.2	1811	D	no	Unknown	EuropeW	4	Bibliography
marjolin	Val de Marne rose garden	15	100.0	1842	HGal	no	Roeser	FranceParis	4	Bibliography
marquise_boccella	Jumaju rose garden	5	61.1	1842	HP	no	Desprez	FranceParis	4	Bibliography
marquise_d_exeter	Val de Marne rose garden	5	86.6	1877	HP	no	Laxton	EuropeN	4	Bibliography
marquise_de_mortemart	Val de Marne rose garden	5	86.5	1868	HP	no	Liabaud	FranceLyon	4	Bibliography
mary_pochin	Val de Marne rose garden	2	61.8	1881	HP	no	Pochin	EuropeN	4	Bibliography
maupertuis	Loubert rose garden	2	86.3	1868	M	no	Moreau_&_Robert	FranceAngers	4	Measured
may_queen	Loubert rose garden	7	41.6	1898	HWich	no	Van_Fleet	America	2	Bibliography
meg_merrilies	Loubert rose garden	14	100.0	1893	HEg	no	Penzance	EuropeN	6	Measured
melanie_waldor	La Cour de Commer rose garden	4	55.5	1865	M	no	Moreau_&_Robert	FranceAngers	4	Bibliography
melle_de_sombreuil	Loubert rose garden	9	61.2	1851	T	no	Robert	FranceAngers	4	Measured
menage	La Cour de Commer rose garden	2	93.8	1847	A	no	Vibert	FranceAngers	4	Bibliography
mercedes	Loubert rose garden	2	93.1	1847	HGal	no	Vibert	FranceAngers	4	Measured
merveille_de_lyon	Grande roseraie de Lyon	5	50.0	1882	HP	no	Pernet	FranceLyon	4	Bibliography
meteor	Loubert rose garden	6	63.4	1836	N	no	Geschwind	EuropeW	4	Measured
mignon	Val de Marne rose garden	9	53.4	1881	Pol	no	Ducher	FranceLyon	2	Bibliography
mignonette	Jumaju rose garden	10	97.8	1881	Pol	no	Guillot	FranceLyon	2	Bibliography
minette	Loubert rose garden	3	64.2	1819	C	no	Vibert	FranceParis	4	Measured
miniature	Val de Marne rose garden	10	99.6	1885	Pol	no	Alégatière	FranceLyon	2	Bibliography
minnehaha	Val de Marne rose garden	11	46.5	1902	HWich	no	Walsh	America	2	Bibliography
miranda	Loubert rose garden	3	50.5	1869	P	no	Sansal	FranceParis	4	Measured
mistress_f_w_flight	Val de Marne rose garden	10	45.5	1906	HMult	no	Cutbush	EuropeN	Unknown	Unknown
mistress_g_dickson	Val de Marne rose garden	6	63.4	1885	HP	no	Bennett	EuropeN	4	Bibliography
mistress_reynolds_hole	SCRADH	6	34.6	1901	T	no	Nabonnand	FranceSE	2	Bibliography
mistress_rumsey	Val de Marne rose garden	9	36.4	1899	HP	no	Rumsey	Unknown	4	Bibliography
mistress_w_h_cutbush	Val de Marne rose garden	10	87.2	1904	Pol	no	Levavasseur	FranceOrleans	2	Bibliography
mle_blanche_durrschmidt	Val de Marne rose garden	6	64.5	1877	T	no	Guillot	FranceLyon	4	Measured
mle_cecile_brunner	Loubert rose garden	9	66.2	1880	Pol	no	Ducher	FranceLyon	2	Bibliography
mle_cecile_brunner	Désert rose garden	7	41.9	1880	Pol	no	Ducher	FranceLyon	2	Bibliography
mle_cecile_brunner_cl	Désert rose garden	9	66.8	1894	Pol	no	Hosp	America	2	Bibliography
mle_elisa_lemasson	Val de Marne rose garden	6	38.1	1849	HT	no	Vibert	FranceAngers	4	Bibliography
mle_genevieve_godard	Désert rose garden	9	56.0	1889	T	no	Godard	FranceLyon	2	Bibliography
mle_josephine_guyot	Val de Marne rose garden	6	62.0	1863	B	no	Touvais	FranceParis	4	Bibliography
mle_marie_dauvesse	Jumaju rose garden	4	61.3	1859	HP	no	Vigneron	FranceAngers	Unknown	Unknown
mle_marie_gaze	Jumaju rose garden	9	72.3	1892	N	no	Godard	FranceLyon	2	Measured
mle_marie_van_houtte	Désert rose garden	9	69.8	1871	T	no	Ducher	FranceLyon	2	Bibliography
mme_abel_chatenay	Loubert rose garden	9	71.7	1894	HT	no	Pernet-Ducher	FranceLyon	2	Measured
mme_alexandre_julien	Val de Marne rose garden	8	86.9	1882	HP	no	Vigneron	FranceAngers	4	Bibliography
mme_alexandre_pommery	Val de Marne rose garden	5	54.7	1882	HP	no	Lévêque	FranceParis	4	Bibliography
mme_alfred_carriere	Val de Marne rose garden	9	72.4	1875	N	no	Schwartz	FranceLyon	3	Measured
mme_alfred_de_rougemont	Val de Marne rose garden	9	69.6	1862	HP	no	Lacharme	FranceLyon	Unknown	Unknown
mme_alice_dureau	Val de Marne rose garden	5	72.4	1867	HP	no	Vigneron	FranceAngers	4	Bibliography
mme_alphonse_seux	Jumaju rose garden	2	80.2	1887	HP	no	Liabaud	FranceLyon	4	Bibliography
mme_anatole_leroy	Val de Marne rose garden	6	68.1	1892	HP	no	Leroy	FranceAngers	4	Bibliography
mme_antoine_mari	Loubert rose garden	9	54.7	1890	T	no	Mari	FranceSE	2	Bibliography
mme_azelie_imbert	Jardin botanique de la Tête d'Or	9	46.6	1870	T	no	Levet	FranceLyon	Unknown	Unknown
mme_berard	Grande roseraie de Lyon	8	75.9	1870	T	no	Levet	FranceLyon	2	Bibliography
mme_bertha_mackart	Val de Marne rose garden	6	68.9	1883	HP	no	Verdier	FranceParis	4	Bibliography
mme_blanche_lafitte	Val de Marne rose garden	8	42.3	1851	B	no	Pradel	FranceSW	4	Measured
mme_boll	Loubert rose garden	4	72.9	1858	HP	no	Boll	America	4	Measured
mme_caroline_kuster	Val de Marne rose garden	9	74.2	1872	N	no	Pernet	FranceLyon	3	Measured
mme_charles_verdier	Val de Marne rose garden	5	90.4	1863	HP	no	Lacharme	FranceLyon	4	Bibliography
mme_constans	Val de Marne rose garden	6	72.0	1902	HWich	no	Roseraie_de_l'Haÿ-Les-Roses	FranceParis	2	Bibliography
mme_crosy	Val de Marne rose garden	5	79.9	1881	HP	no	Levet	FranceLyon	4	Bibliography
mme_cunisset_carnot	Val de Marne rose garden	8	57.9	1899	HT	no	Buatois	FranceNE	4	Bibliography
mme_d_arblay	Loubert rose garden	7	36.6	1835	HMult	no	Wells	EuropeN	3	Measured
mme_d_hebray	Loubert rose garden	2	93.8	1857	C	no	Pradel	FranceSW	4	Measured
mme_dailleux	Val de Marne rose garden	8	95.3	1900	HT	no	Buatois	FranceNE	4	Bibliography
mme_de_la_roche_lambert	Loubert rose garden	4	72.4	1851	M	no	Robert	FranceAngers	4	Measured
mme_de_stael	Loubert rose garden	4	58.8	1857	M	no	Moreau_&_Robert	FranceAngers	4	Measured
mme_de_watteville	Grande roseraie de Lyon	9	66.4	1883	T	no	Guillot	FranceLyon	2	Bibliography
mme_desire_giraud	Val de Marne rose garden	5	84.4	1854	HP	no	Haussy	France	4	Bibliography
mme_e_veyrat_hermanos	Loubert rose garden	9	66.5	1895	T	no	Bernaix	FranceLyon	2	Bibliography
mme_edouard_herriot	Grande roseraie de Lyon	8	63.5	1913	HT	no	Pernet-Ducher	FranceLyon	Unknown	Unknown
mme_edouard_michel	Val de Marne rose garden	5	71.1	1886	HP	no	Verdier	FranceParis	4	Bibliography
mme_edouard_ory	Loubert rose garden	5	67.4	1854	M	no	Robert	FranceAngers	4	Measured
mme_emilie_charron	Loubert rose garden	9	60.5	1895	T	no	Perrier	FranceLyon	2	Measured
mme_ernest_calvat	Loubert rose garden	8	96.2	1888	B	no	Schwartz	FranceLyon	4	Bibliography
mme_ernest_piard	Loubert rose garden	8	41.7	1887	HT	no	Bonnaire	FranceLyon	4	Measured
mme_eugene_resal	Grande roseraie de Lyon	9	67.5	1895	Ch	no	Guillot	FranceLyon	Unknown	Unknown
mme_falcot	Désert rose garden	9	57.7	1858	T	no	Guillot	FranceLyon	2	Bibliography
mme_fanny_pauwels	Désert rose garden	9	54.9	1884	T	no	Soupert_&_Notting	EuropeW	2	Bibliography
mme_fillion	Jumaju rose garden	8	54.8	1865	HP	no	Gonod	FranceLyon	4	Bibliography
mme_fortune_besson	Val de Marne rose garden	8	86.8	1881	HP	no	Besson	FranceLyon	4	Bibliography
mme_francois_pittet	Jumaju rose garden	6	64.4	1877	B	no	Lacharme	FranceLyon	4	Measured
mme_georges_delbard	La Beaujoire rose garden	8	74.3	1982	HT	no	Delbard	FranceParis	4	Bibliography
mme_hardy	Loubert rose garden	3	50.1	1832	D	no	Hardy	FranceParis	4	Measured
mme_henry	Jardin botanique de la Tête d'Or	9	66.2	1879	N	no	Ducher	FranceLyon	2	Bibliography
mme_hersilie_ortgies	Val de Marne rose garden	8	96.6	1868	HP	no	Soupert_&_Notting	EuropeW	4	Bibliography
mme_j_bonnaire_pierre	Val de Marne rose garden	6	68.3	1891	HT	no	Bonnaire	FranceLyon	4	Bibliography
mme_jean_dupuy	Grande roseraie de Lyon	9	62.4	1901	T	no	Lambert	EuropeW	2	Bibliography
mme_joseph_schwartz	Désert rose garden	9	50.5	1880	T	no	Schwartz	FranceLyon	2	Bibliography
mme_josephine_burland	Jumaju rose garden	9	44.4	1886	Pol	no	Bernaix	FranceLyon	Unknown	Unknown
mme_jules_bouche	Grande roseraie de Lyon	5	68.5	1910	HT	no	Croibier	FranceLyon	4	Bibliography
mme_jules_finger	Val de Marne rose garden	8	67.6	1893	HT	no	Guillot	FranceLyon	4	Bibliography
mme_jules_franke	SCRADH	9	50.4	1887	N	no	Nabonnand	FranceSE	4	Measured
mme_jules_gravereaux	Grande roseraie de Lyon	8	65.8	1900	T	no	Soupert_&_Notting	EuropeW	2	Bibliography
mme_jules_thibaud	Jumaju rose garden	9	40.9	1830	Pol	no	Unknown	Unknown	2	Bibliography
mme_julie_lasseu	Désert rose garden	6	67.2	1881	N	no	Nabonnand	FranceSE	4	Measured
mme_knorr	Val de Marne rose garden	4	72.1	1865	P	no	Verdier	FranceParis	4	Bibliography
mme_la_duchesse_d_auerstadt	Jardin botanique de la Tête d'Or	9	73.3	1887	N	no	Bernaix	FranceLyon	2	Bibliography
mme_la_princesse_de_radziwill	Désert rose garden	9	51.3	1886	T	no	Nabonnand	FranceSE	2	Bibliography
mme_lacharme	Val de Marne rose garden	8	93.4	1872	HT	no	Lacharme	FranceLyon	4	Bibliography
mme_lambard	Loubert rose garden	9	51.7	1878	T	no	Lacharme	FranceLyon	2	Measured
mme_landeau	La Cour de Commer rose garden	2	91.1	1873	M	no	Moreau_&_Robert	FranceAngers	4	Bibliography
mme_laurent_simons	Désert rose garden	9	46.4	1894	T	no	Lévêque	FranceParis	2	Bibliography
mme_laurette_messimy	Jumaju rose garden	9	55.0	1887	Ch	no	Guillot	FranceLyon	2	Bibliography
mme_lauriol_de_barny	Loubert rose garden	6	63.0	1867	B	no	Trouillard	FranceAngers	3	Bibliography
mme_legrand	La Cour de Commer rose garden	3	52.1	1863	M	no	Fontaine	FranceParis	4	Bibliography
mme_legras_de_st_germain	Loubert rose garden	2	50.9	1846	A	no	Unknown	Unknown	4	Measured
mme_leon_constantin	Grande roseraie de Lyon	8	96.5	1908	T	no	Bonnaire	FranceLyon	2	Bibliography
mme_letuve_de_colnet	Val de Marne rose garden	8	96.0	1887	B	no	Vigneron	FranceAngers	4	Bibliography
mme_lierval	Loubert rose garden	6	59.6	1869	HP	no	Fontaine	FranceParis	4	Measured
mme_louis_blanchet	Val de Marne rose garden	10	99.5	1894	N	no	Godard	FranceLyon	4	Measured
mme_louis_henry	Val de Marne rose garden	9	57.7	1879	N	no	Ducher	FranceLyon	3	Measured
mme_louis_leveque	Loubert rose garden	5	66.4	1898	M	no	Lévêque	FranceParis	4	Measured
mme_lucien_chaure	Val de Marne rose garden	6	64.3	1884	HP	no	Vigneron	FranceAngers	4	Bibliography
mme_lureau_escalais	Val de Marne rose garden	6	50.6	1886	HP	no	Verdier	FranceParis	4	Bibliography
mme_marthe_d_halloy	Jumaju rose garden	6	44.1	1881	HP	no	Lévêque	FranceParis	4	Bibliography
mme_massot	Val de Marne rose garden	9	33.0	1875	B	no	Lacharme	FranceLyon	Unknown	Unknown
mme_maurice_rivoire	Val de Marne rose garden	6	61.2	1878	HP	no	Gonod	FranceLyon	4	Bibliography
mme_montet	Val de Marne rose garden	6	70.3	1880	HP	no	Liabaud	FranceLyon	4	Bibliography

mme_nerard	Val de Marne rose garden	6	59.6	1838	B	no	Nerard	FranceLyon	4	Bibliography
mme_p_perny	Désert rose garden	9	46.6	1879	T	no	Nabonnand	FranceSE	2	Bibliography
mme_philippe_plantamour	Val de Marne rose garden	10	93.7	1898	HRg	no	Unknown	Unknown	2	Bibliography
mme_pierre_cochet	Jumaju rose garden	9	74.3	1891	N	no	Cochet	FranceParis	2	Measured
mme_plantier	Val de Marne rose garden	4	68.4	1835	A	no	Plantier	FranceLyon	3	Bibliography
mme_platz	La Cour de Commer rose garden	4	68.5	1864	M	no	Moreau_&_Robert	FranceAngers	4	Bibliography
mme_renahy	Jumaju rose garden	6	56.9	1889	HP	no	Guillot	FranceLyon	4	Bibliography
mme_rose_cheri	La Cour de Commer rose garden	4	62.1	1850	M	no	Laffay	FranceParis	4	Bibliography
mme_scipion_cochet	Val de Marne rose garden	8	93.8	1872	HP	no	Cochet	FranceParis	4	Bibliography
mme_segond_weber	Val de Marne rose garden	8	87.2	1907	HT	no	Soupert_&_Notting	EuropeW	4	Bibliography
mme_therese_vernes	Val de Marne rose garden	5	86.0	1891	HP	no	Lévêque	FranceParis	4	Bibliography
mme_van_houtte	Val de Marne rose garden	6	48.5	1857	HP	no	Margottin	FranceParis	4	Bibliography
mme_verlot	Val de Marne rose garden	5	85.1	1876	HP	no	Verdier	FranceParis	4	Bibliography
mme_verrier_cachet	Jumaju rose garden	3	77.0	1895	HP	no	Chédane-Guinoisseau	FranceAngers	4	Bibliography
mme_victor_verdier	Grande roseraie de Lyon	8	95.9	1859	HP	no	Verdier	FranceParis	4	Bibliography
mme_vve_alexis_pommery	Jumaju rose garden	5	63.0	1882	HP	no	Lévêque	FranceParis	4	Bibliography
mme_zoetmans	Loubert rose garden	2	81.8	1830	D	no	Marest	FranceParis	4	Measured
mogador	Loubert rose garden	7	33.5	1846	P	no	Unknown	France	2	Measured
moïse	Loubert rose garden	4	76.0	1828	HGal	no	Parmentier	EuropeW	3	Measured
monsieur_cordeau	Loubert rose garden	6	69.4	1892	B	no	Moreau_&_Robert	FranceAngers	4	Measured
monsieur_rosier	Grande roseraie de Lyon	9	65.7	1897	T	no	Nabonnand	FranceSE	2	Bibliography
montalembert	Loubert rose garden	4	60.2	1861	HGal	no	Moreau_&_Robert	FranceAngers	3	Measured
montigny	Val de Marne rose garden	2	89.3	1842	HGal	no	Unknown	Unknown	4	Bibliography
mousseuse_blanche_nouvelle	La Cour de Commer rose garden	4	72.9	1807	M	no	Salter	EuropeN	4	Bibliography
mousseux_du_japon	La Cour de Commer rose garden	2	92.6	1899	M	no	Unknown	Unknown	4	Bibliography
mr_francisque_rive	Jumaju rose garden	2	61.2	1883	HP	no	Schwartz	FranceLyon	4	Bibliography
mr_le_capitaine_louis_frere	Jumaju rose garden	6	66.6	1883	HP	no	Vigneron	FranceAngers	4	Bibliography
mr_pelisson	La Cour de Commer rose garden	10	76.0	1849	M	no	Vibert	FranceAngers	4	Bibliography
mrs_colville	Jumaju rose garden	4	60.5	1869	HSpn	no	Moreau_&_Robert	FranceAngers	4	Bibliography
mrs_dudley_cross	Désert rose garden	9	59.5	1907	T	no	Paul	EuropeN	2	Bibliography
mrs_reynolds_hole	Désert rose garden	6	34.6	1901	T	no	Nabonnand	FranceSE	2	Bibliography
multiflore_de_vaumarcus	Jumaju rose garden	9	48.2	1875	N	no	Menet	Unknown	2	Bibliography
n_j_kicunov	Jumaju rose garden	16	100.0	1898	A	no	Micurin	EuropeE	Unknown	Unknown
nankin	Val de Marne rose garden	13	100.0	1815	HSpn	no	Descemet	FranceParis	4	Bibliography
narcisse_de_salvandy	Loubert rose garden	2	78.3	1843	HGal	no	Parmentier	EuropeW	4	Measured
narrow_water	Loubert rose garden	9	47.3	1883	N	no	Daisy_Hill_Nursery	EuropeN	2	Measured
narrow_water	Loubert rose garden	9	49.7	1883	N	no	Daisy_Hill_Nursery	EuropeN	2	Measured
nastarana	Val de Marne rose garden	10	99.2	1880	N	no	Christ	France	2	Measured
nathalie	Loubert rose garden	8	87.1	1835	N	no	Vibert	FranceParis	4	Measured
neron	Val de Marne rose garden	2	60.4	1841	HGal	no	Laffay	FranceParis	4	Bibliography
new_dawn	Val de Marne rose garden	2	86.0	1930	LCl	no	Somerset_Rose_Nursery	America	3	Bibliography
niphetos	Désert rose garden	8	72.4	1835	T	no	Bougère	FranceAngers	2	Bibliography
noella_nabonnand	SCRADH	9	29.7	1900	T	no	Nabonnand	FranceSE	3	Bibliography
noisette_moschata	Val de Marne rose garden	6	37.9	1873	N	no	Schwartz	FranceLyon	4	Measured
noisette_moschata	Val de Marne rose garden	6	48.0	1873	N	no	Schwartz	FranceLyon	4	Measured
nouveau_intelligible	La Cour de Commer rose garden	2	86.0	1811	HGal	no	Unknown	Unknown	4	Bibliography
nouveau_vulcain	Val de Marne rose garden	2	92.6	1861	HGal	no	Unknown	France	4	Bibliography
nouvelle_pivoine	Val de Marne rose garden	2	82.2	1817	HGal	no	Unknown	EuropeW	4	Bibliography
nouvelle_transparente	Loubert rose garden	2	94.0	1817	HGal	no	Miellez	FranceNW	4	Measured
œillet_de_damas	Loubert rose garden	1	53.9	1835	D	no	Prevost	FranceNW	4	Measured
oeillet_double	Jumaju rose garden	2	62.3	1841	HGal	no	Prevost	FranceNW	4	Bibliography
œillet_flamand	Val de Marne rose garden	8	77.1	1845	HGal	no	Vibert	FranceAngers	4	Bibliography
oeillet_panache	Loubert rose garden	2	76.5	1887	M	no	Verdier	FranceParis	4	Bibliography
œillet_parfait	Val de Marne rose garden	2	61.0	1841	HGal	no	Foulard	FranceAngers	4	Bibliography
ohl	Val de Marne rose garden	2	90.6	1843	HGal	no	Vibert	FranceAngers	4	Bibliography
old_blush	INRA Angers	9	73.9	1793	Ch	no	Parsons	China	2	Measured
old_crimson_china	Jumaju rose garden	9	53.4	1733	Ch	no	Unknown	China	2	Bibliography
olga_marix	Jardin botanique de la Tête d'Or	6	67.3	1873	B	no	Schwartz	FranceLyon	3	Measured
olivet	Loubert rose garden	6	62.0	1892	HMult	no	Vigneron	FranceAngers	4	Measured
ombre_panachee	La Cour de Commer rose garden	15	100.0	1811	HGal	no	Unknown	America	4	Bibliography
ombree_parfaite	La Cour de Commer rose garden	4	50.3	1823	HGal	no	Vibert	FranceParis	4	Bibliography
omer_pacha	Loubert rose garden	8	62.8	1853	B	no	Pradel	FranceSW	4	Bibliography
orleans_rose	Val de Marne rose garden	10	98.4	1909	Pol	no	Levavasseur	FranceOrleans	2	Bibliography
ornement_de_la_nature	La Cour de Commer rose garden	2	95.2	1814	HGal	no	Unknown	EuropeW	4	Bibliography
ornement_des_bosquets	Loubert rose garden	9	71.0	1860	HSem	no	Jamain	FranceParis	Unknown	Unknown
ornement_des_bosquets	Val de Marne rose garden	9	82.7	1860	HSem	no	Jamain	FranceParis	Unknown	Unknown
oscar_chauvry	Val de Marne rose garden	10	99.6	1900	N	no	Chauvry	FranceSW	4	Measured
oscar_leclerc	La Cour de Commer rose garden	15	100.0	1846	M	no	Robert	FranceAngers	4	Bibliography
ovid	Val de Marne rose garden	6	43.4	1890	HSet	no	Geschwind	EuropeW	2	Bibliography
paeonienrose	La Cour de Commer rose garden	2	50.5	1845	HGal	no	Unknown	Unknown	4	Bibliography
panachee_superbe	Loubert rose garden	4	53.5	1811	HGal	no	Unknown	EuropeW	4	Measured
papa_gontier_cl	Désert rose garden	9	45.5	1882	T	no	Hosp	America	2	Bibliography
papa_meilland	Grande roseraie de Lyon	8	52.6	1963	HT	no	Meilland	FranceSE	4	Bibliography
papillon	SCRADH	9	74.9	1878	T	no	Nabonnand	FranceSE	2	Bibliography
paquerette	Loubert rose garden	10	96.4	1875	Pol	no	Guillot	FranceLyon	2	Measured
paquita	La Cour de Commer rose garden	2	71.0	1841	HGal	no	Unknown	Unknown	4	Bibliography
park_wilhelmshöhe	La Cour de Commer rose garden	4	69.4	1987	HGal	no	Kordes	EuropeW	4	Bibliography
parks_yellow_tea_scented_china	Loubert rose garden	10	98.7	1824	T	no	Unknown	China	2	Measured
parmentier	La Cour de Commer rose garden	3	72.3	1860	HP	no	Guillot	FranceLyon	4	Bibliography
paul_nabonnand	Loubert rose garden	8	59.9	1878	T	no	Nabonnand	FranceSE	2	Measured
paul_neyron	Grande roseraie de Lyon	10	30.9	1869	HP	no	Levet	FranceLyon	4	Bibliography
paul_noel	Val de Marne rose garden	2	58.8	1913	HWich	no	Tanne	FranceNW	2	Bibliography
paul_ricault	La Cour de Commer rose garden	5	75.2	1845	C	no	Portemer	FranceParis	4	Bibliography
paul_s_early_blush	Loubert rose garden	6	67.6	1893	HP	no	Paul	EuropeN	4	Measured
paul_s_himalayan_musk	Désert rose garden	10	99.6	1916	HMsk	no	Paul	EuropeN	Unknown	Unknown
paul_transon	Jumaju rose garden	10	99.4	1900	HWich	no	Barbier	FranceOrleans	4	Bibliography
paul_verdier	Loubert rose garden	5	72.6	1866	HP	no	Verdier	FranceParis	4	Measured
pauline_bonaparte	Val de Marne rose garden	8	97.2	1850	B	no	Laffay	FranceParis	Unknown	Unknown
pavillon_de_pregny	Jumaju rose garden	6	46.5	1863	N	no	Guillot	FranceLyon	2	Bibliography
pergolese	Loubert rose garden	3	54.9	1840	P	no	Moreau_&_Robert	FranceAngers	4	Measured
perle_d_angers	Loubert rose garden	6	72.6	1879	B	no	Moreau_&_Robert	FranceAngers	4	Measured
perle_des_blanches	Jumaju rose garden	9	30.8	1872	N	no	Lacharme	FranceLyon	Unknown	Unknown
perle_des_jardins	Désert rose garden	9	67.3	1874	T	no	Levet	FranceLyon	2	Bibliography
persian_yellow	Loubert rose garden	13	100.0	1837	Sp	yes	Willock	Middle_East	4	Measured
persian_yellow	Loubert rose garden	13	100.0	1837	Sp	yes	Willock	Middle_East	4	Measured
persian_yellow	Loubert rose garden	13	100.0	1837	Sp	yes	Willock	Middle_East	4	Bibliography
persian_yellow1	Loubert rose garden	13	100.0	1837	Sp	yes	Willock	Middle_East	4	Measured
petit_louis	Val de Marne rose garden	11	47.8	1919	HWich	no	Nonin	FranceParis	2	Bibliography
petite_de_hollande	La Cour de Commer rose garden	2	56.7	1791	C	no	Unknown	EuropeW	4	Bibliography
petite_ecossaise	Loubert rose garden	13	88.0	1826	HSpn	no	Vibert	FranceParis	4	Measured
petite_leonie	Loubert rose garden	9	45.5	1892	Pol	no	Soupert_&_Notting	EuropeW	2	Measured
petite_lisette	Loubert rose garden	14	100.0	1817	A	no	Vibert	FranceParis	4	Measured
petite_orleanaise	Val de Marne rose garden	3	64.8	1900	HGal	no	Unknown	Unknown	4	Bibliography
philemon_cochet	La Beaujoire rose garden	8	96.4	1895	B	no	Cochet	FranceParis	4	Bibliography
philomele	Val de Marne rose garden	6	63.4	1844	N	no	Vibert	FranceAngers	4	Measured
pierre_de_ronsard	La Beaujoire rose garden	8	70.8	1985	LCl	no	Meilland	FranceSE	Unknown	Unknown
pierre_de_saint_cyr	Val de Marne rose garden	9	37.9	1838	B	no	Plantier	FranceLyon	4	Bibliography
pink_pearl_cl	Val de Marne rose garden	7	38.7	1913	HT	no	Hobbies	EuropeN	4	Bibliography
pink_roamer	Val de Marne rose garden	9	36.1	1890	HWich	no	Horvath	America	2	Bibliography
pink_rover	Val de Marne rose garden	8	87.8	1891	HT	no	Paul	EuropeN	4	Bibliography
pompon_blanc_parfait	Loubert rose garden	6	45.5	1876	A	no	Verdier	FranceParis	4	Measured
pompon_de_bourgogne	Jumaju rose garden	2	73.9	1664	HGal	no	Unknown	France	4	Bibliography
pompon_de_paris	Loubert rose garden	9	70.8	1800	Ch	no	Colville	Unknown	2	Measured
pompon_de_paris_cl	Jardin botanique de la Tête d'Or	9	68.0	1839	Ch	no	Unknown	Unknown	Unknown	Unknown
pompon_panache	Loubert rose garden	2	91.6	1858	HGal	no	Moreau_&_Robert	FranceAngers	4	Measured
ponctuee	Val de Marne rose garden	4	63.4	1829	M	no	Herbert	France	4	Bibliography
poppuis	Loubert rose garden	13	98.8	1838	HSpn	no	Stenberg	Unknown	3	Measured
pourpre	Jumaju rose garden	7	43.7	1823	Ch	no	Vibert	FranceParis	Unknown	Unknown
pourpre_ancien	Loubert rose garden	12	99.7	1820	Misc_OGR	no	Unknown	Unknown	2	Bibliography

pourpre_charmant	La Cour de Commer rose garden	2	89.4	1811	HGal	no	Unknown	America	4	Bibliography
president_dutailly	Val de Marne rose garden	2	91.1	1888	HGal	no	Dubreuil	FranceLyon	4	Bibliography
president_gausen	Val de Marne rose garden	8	96.3	1862	B	no	Pradel	FranceSW	4	Bibliography
prince_albert	Val de Marne rose garden	8	77.0	1852	B	no	Fontaine	FranceParis	4	Bibliography
prince_camille_de_rohan	Loubert rose garden	8	66.2	1861	HP	no	Verdier	FranceParis	4	Measured
prince_charles	Loubert rose garden	4	68.2	1842	B	no	Hardy	FranceParis	Unknown	Unknown
prince_charles_d_arenberg	Jumaju rose garden	3	55.8	1887	HP	no	Soupert_&_Notting	EuropeW	4	Bibliography
prince_eugene_de_beauharnais	Jumaju rose garden	2	87.3	1864	HP	no	Pernet	FranceLyon	Unknown	Unknown
prince_frederic	La Cour de Commer rose garden	3	49.0	1840	HGal	no	Parmentier	EuropeW	4	Bibliography
prince_noir	Loubert rose garden	8	55.5	1854	HP	no	Boyau	FranceAngers	4	Measured
princess_of_wales	Jumaju rose garden	5	87.0	1871	HP	no	Laxton	EuropeN	4	Bibliography
princesse_amelie	La Cour de Commer rose garden	15	100.0	1872	M	no	Robert	FranceAngers	4	Bibliography
princesse_de_lamballe	Loubert rose garden	16	100.0	1830	A	no	Miellez	FranceNW	6	Measured
princesse_de_nassau	Val de Marne rose garden	2	77.9	1829	N	no	Laffay	FranceParis	Unknown	Unknown
princesse_de_sagan	Loubert rose garden	9	64.8	1887	Ch	no	Dubreuil	FranceLyon	2	Measured
princesse_josephine_de_flandre	Val de Marne rose garden	9	45.0	1888	Pol	no	Soupert	EuropeW	2	Bibliography
princesse_louise	Loubert rose garden	10	89.8	1828	HSem	no	Jacques	FranceParis	2	Measured
princesse_marie	Loubert rose garden	10	88.5	1821	HSem	no	Jacques	FranceParis	2	Bibliography
princesse_radziwill	Val de Marne rose garden	8	52.6	1886	HP	no	Lévêque	FranceParis	4	Bibliography
princesse_royale	La Cour de Commer rose garden	9	59.1	1846	M	no	Portemer	FranceParis	4	Bibliography
professeur_ganiviat	Loubert rose garden	9	69.6	1890	T	no	Perrier	FranceLyon	2	Measured
provence_pink	Loubert rose garden	2	73.5	1450	C	no	Unknown	EuropeW	4	Measured
provins_ancien	Val de Marne rose garden	15	100.0	1853	HGal	no	Cochet	FranceParis	4	Bibliography
provins_renoncule	Loubert rose garden	2	87.3	1810	HGal	no	Dupont	FranceParis	4	Measured
pucelle_de_lille	Loubert rose garden	2	92.6	1828	HGal	no	Miellez	FranceNW	4	Measured
pucelle_de_lille1	Loubert rose garden	2	95.0	1828	HGal	no	Miellez	FranceNW	4	Measured
purezza	Désert rose garden	10	97.2	1961	LCl	no	Mansuino	EuropeS	Unknown	Unknown
purpurea_rubra	La Cour de Commer rose garden	3	68.8	1849	M	no	Unknown	Unknown	4	Bibliography
quatre_saisons_blanc_mousseux	Loubert rose garden	3	61.6	1830	M	no	Laffay	FranceParis	4	Measured
quatre_saisons_blanc_mousseux	Loubert rose garden	3	70.1	1830	M	no	Laffay	FranceParis	4	Measured
quatre_saisons_continues	Loubert rose garden	4	65.3	1633	D	no	Unknown	France	4	Measured
queen_des_prairies	Jardin botanique de la Tête d'Or	9	51.7	1843	HSet	no	Feast	America	3	Bibliography
queen_elizabeth	Jardin botanique de la Tête d'Or	15	100.0	1954	Gr	no	Lammerts	America	4	Bibliography
queen_mab	Jumaju rose garden	9	63.8	1896	Ch	no	Paul	EuropeN	Unknown	Unknown
red_damask	Loubert rose garden	2	88.9	1160	Sp	no	Unknown	Europe	4	Bibliography
red_intuition	INRA Angers	10	98.0	1999	HT	no	Delbard	FranceParis	4	Bibliography
reine_d_espagne	Val de Marne rose garden	2	84.6	1861	HP	no	Fontaine	FranceParis	4	Bibliography
reine_de_belgique	Loubert rose garden	10	70.9	1845	Unknown	no	Unknown	Unknown	Unknown	Unknown
reine_des_belges	Jardin botanique de la Tête d'Or	10	69.9	1832	Ayr	no	Jacques	FranceParis	2	Bibliography
reine_des_centfeuilles	Loubert rose garden	2	68.3	1824	C	no	Unknown	EuropeW	4	Measured
reine_des_mousseuses	La Cour de Commer rose garden	2	60.4	1860	M	no	Moreau_&_Robert	FranceAngers	4	Bibliography
reine_des_violettes	Loubert rose garden	5	88.7	1860	HP	no	Millet-Mallet	FranceNW	4	Bibliography
rene_andre	Loubert rose garden	9	46.8	1901	HWich	no	Barbier	FranceOrleans	2	Measured
renoncule	Val de Marne rose garden	10	89.7	1913	Pol	no	Barbier	FranceOrleans	2	Bibliography
repens	Val de Marne rose garden	9	57.1	1829	N	no	Garcon	FranceNW	4	Measured
reve_d_or	Jardin botanique de la Tête d'Or	9	77.0	1860	N	no	Ducher	FranceLyon	2	Measured
river_s_george_iv	Loubert rose garden	8	97.5	1820	HCh	no	Rivers	EuropeN	4	Measured
robert_le_diable	Loubert rose garden	2	85.1	1837	HGal	no	Unknown	Unknown	4	Measured
robusta	Val de Marne rose garden	9	34.0	1877	B	no	Soupert	EuropeW	4	Bibliography
robusta_bourbon	Loubert rose garden	7	36.7	1877	B	no	Soupert_&_Notting	EuropeW	4	Measured
rodhatte	Val de Marne rose garden	8	40.8	1912	F	no	Poulsen	EuropeN	3	Bibliography
roger_lambelin	Grande roseraie de Lyon	6	46.4	1890	HP	no	Schwartz	FranceLyon	4	Bibliography
roi_de_siam	Loubert rose garden	6	37.7	1825	T	no	Laffay	FranceParis	3	Measured
roi_des_aunes	Loubert rose garden	10	65.5	1884	S	no	Geschwind	EuropeW	2	Measured
roi_des_pays_bas	Jumaju rose garden	3	60.8	1824	D	no	Unknown	EuropeW	4	Bibliography
roi_des_pourpres	Loubert rose garden	4	66.9	1819	HGal	no	Descemet	FranceParis	4	Measured
rosa_acicularis	Jardin botanique de la Tête d'Or	12	100.0	Unknown	Sp	yes	Unknown	America	6	Bibliography
rosa_agrestis	Loubert rose garden	14	98.2	1878	Sp	yes	Unknown	Europe	5	Bibliography
rosa_alba	Val de Marne rose garden	16	100.0	1597	Sp	no	Unknown	Europe	6	Bibliography
rosa_alba_maxima	Loubert rose garden	16	100.0	1500	A	no	Unknown	Europe	6	Bibliography
rosa_alba_semi_plena	Loubert rose garden	16	100.0	1629	A	no	Unknown	Europe	6	Bibliography
rosa_albertii	Loubert rose garden	12	100.0	1877	Sp	yes	Regel	Asia	4	Measured
rosa_amblyotis	Jardin botanique de la Tête d'Or	12	99.0	1847	Sp	yes	Unknown	Asia	2	Bibliography
rosa_andrae	Loubert rose garden	12	100.0	1875	Sp	no	Lange	Asia	4	Measured
rosa_anemonaeflora	Loubert rose garden	12	99.7	1844	Sp	no	Fortune	China	4	Measured
rosa_anemonea	Loubert rose garden	12	99.8	1814	Sp	no	Unknown	Unknown	4	Bibliography
rosa_arkansana	Loubert rose garden	16	97.9	1715	Sp	yes	Unknown	America	5	Measured
rosa_arkansana_corsley_form	Loubert rose garden	14	75.9	1880	Sp	yes	Unknown	America	6	Measured
rosa_arvensis	Loubert rose garden	10	96.6	1750	Sp	yes	Unknown	Europe	2	Measured
rosa_atropurpurea	Loubert rose garden	12	100.0	1790	HGal	no	Unknown	EuropeW	Unknown	Unknown
rosa_aveyronensis	Loubert rose garden	14	100.0	1900	intersp	no	Unknown	France	6	Measured
rosa_banksiae_alba_plena	Private donations	10	99.3	1807	Sp	yes	Unknown	China	2	Bibliography
rosa_banksiae_normalis	Désert rose garden	12	94.9	1796	Sp	yes	Unknown	China	2	Bibliography
rosa_banksiopsis	Loubert rose garden	12	99.9	1907	Sp	yes	Baker	China	2	Bibliography
rosa_beggeriana_nigr	Loubert rose garden	12	99.9	1860	Sp	yes	Unknown	Asia	3	Measured
rosa_bracteata	Loubert rose garden	12	68.0	1793	Sp	yes	Macartney	China	2	Measured
rosa_brunonii	Jardin botanique de la Tête d'Or	10	77.9	Unknown	Sp	yes	Unknown	Asia	2	Bibliography
rosa_californica	Loubert rose garden	10	91.3	1878	Sp	yes	Unknown	America	2	Measured
rosa_californica1	Loubert rose garden	10	92.8	1878	Sp	yes	Unknown	America	2	Measured
rosa_calocarpa	Loubert rose garden	12	99.5	1891	HRg	no	Bruant	FranceAngers	2	Measured
rosa_canina_blondaeana	Loubert rose garden	15	100.0	1861	Sp	yes	Unknown	Europe	6	Measured
rosa_cannabifolia	Loubert rose garden	16	100.0	1800	A	no	Flobert	France	6	Bibliography
rosa_carolina_grandiflora_humilis	La Beaujoire rose garden	12	99.9	1826	Sp	yes	Unknown	America	4	Bibliography
rosa_caudata	Loubert rose garden	12	100.0	1896	Sp	yes	Unknown	China	2	Bibliography
rosa_centifolia_bullata	Jumaju rose garden	1	51.6	1801	C	no	Duhamel	EuropeW	4	Bibliography
rosa_centifolia_muscosa	La Cour de Commer rose garden	2	88.6	1696	M	no	Unknown	EuropeW	4	Bibliography
rosa_centifolia_parvifolia	Val de Marne rose garden	6	48.4	1664	HGal	no	Unknown	France	4	Bibliography
rosa_centifolia_rosea	Loubert rose garden	4	50.1	1863	HP	no	Touvais	FranceParis	4	Measured
rosa_centifolia_simplex	La Cour de Commer rose garden	2	88.8	1817	C	no	Dupont	FranceParis	4	Bibliography
rosa_centifolia_white_moss	Loubert rose garden	2	67.7	1788	M	no	Shailer	EuropeN	4	Bibliography
rosa_chinensis	Loubert rose garden	10	99.4	1759	Sp	yes	Unknown	China	2	Bibliography
rosa_chinensis_jacq_var_chinensis	Flower Research Institute, China	10	84.0	1759	Sp	yes	Unknown	China	2	Measured
rosa_chinensis_mutabilis	Jardin botanique de la Tête d'Or	10	35.0	1894	Ch	no	Unknown	Africa	2	Bibliography
rosa_chinensis_old_blush	Loubert rose garden	9	73.9	1796	Ch	no	Unknown	China	2	Measured
rosa_chinensis_old_blush_cl	Jardin botanique de la Tête d'Or	9	70.8	1752	Ch	no	Unknown	China	2	Bibliography
rosa_chinensis_viridiflora	Loubert rose garden	9	64.4	1856	Ch	yes	Smith	America	2	Bibliography
rosa_clinophylla	INRA Angers	8	97.0	1820	Sp	yes	Whitley	Asia	2	Bibliography
rosa_complicata	Loubert rose garden	6	46.1	1864	HGal	no	Unknown	Unknown	4	Bibliography
rosa_coryana	Loubert rose garden	12	100.0	1732	S	no	Hurst	EuropeN	Unknown	Unknown
rosa_cuspidata	Loubert rose garden	15	99.4	1820	Sp	yes	Unknown	EuropeE	4	Measured
rosa_cymosa	Jardin botanique de la Tête d'Or	10	99.6	1904	Sp	yes	Unknown	Asia	2	Bibliography
rosa_dafugui	Flower Research Institute, China	5	91.2	Unknown	Unknown	no	Unknown	Unknown	4	Measured
rosa_damascena	Val de Marne rose garden	4	50.8	1560	D	no	Unknown	Middle_East	4	Bibliography
rosa_damascena_celsiana	La Beaujoire rose garden	2	92.4	1732	D	no	Cels_de_Holl	EuropeW	4	Bibliography
rosa_dumalis	Loubert rose garden	15	100.0	1872	Sp	yes	Unknown	EuropeE	5	Measured
rosa_dumetorum	Loubert rose garden	15	100.0	1889	Sp	yes	Schmidt	EuropeE	5	Measured
rosa_dupontii	Loubert rose garden	4	71.6	1817	Misc_OGR	no	Dupont	FranceParis	4	Measured
rosa_ecae	Loubert rose garden	12	51.5	1880	Sp	yes	Unknown	Middle_East	2	Bibliography
rosa_elegantula	Jardin botanique de la Tête d'Or	12	98.8	1908	Sp	yes	Wilson	China	2	Bibliography
rosa_excelsa	Val de Marne rose garden	11	40.1	1909	HWich	no	Walsh	America	2	Bibliography
rosa_farreri_persetosa	Val de Marne rose garden	12	99.7	1895	Sp	yes	Wilson	China	2	Bibliography
rosa_fedschenkoana	Loubert rose garden	12	99.8	1871	Sp	yes	Fedtschenko	Asia	4	Measured
rosa_ferruginea	Loubert rose garden	12	99.9	1814	Sp	yes	Unknown	Europe	2	Measured
rosa_filipes	Jardin botanique de la Tête d'Or	12	59.4	1908	Sp	yes	Wilson	China	2	Bibliography
rosa_filipes	Loubert rose garden	10	95.4	1908	Sp	yes	Wilson	China	2	Bibliography
rosa_foetida	Loubert rose garden	13	100.0	1597	Sp	yes	Unknown	Asia	4	Bibliography
rosa_foetida_bicolor	Loubert rose garden	13	100.0	1590	Sp	yes	Unknown	Middle_East	4	Bibliography
rosa_fortiniana_de_banks	Private donations	12	98.2	1840	Unknown	no	Fortune	China	2	Bibliography
rosa_froebelii	Loubert rose garden	15	100.0	1889	Sp	yes	Unknown	EuropeE	5	Measured

rosa_gallica	Jardin botanique de la Tête d'Or	2	72.8	1500	Sp	yes	Unknown	EuropeE	4	Bibliography
rosa_gallica	Loubert rose garden	2	62.4	1500	Sp	yes	Unknown	EuropeE	4	Measured
rosa_gallica_officinalis	Loubert rose garden	2	57.0	1160	Sp	yes	Unknown	EuropeE	4	Bibliography
rosa_gallica_pumila	Loubert rose garden	3	71.3	1774	HGal	yes	Unknown	EuropeS	4	Bibliography
rosa_gallica_tricolore	Val de Marne rose garden	2	90.1	1815	HGal	no	Stegerhoek	EuropeW	4	Bibliography
rosa_gallica_velutinaeflora	La Cour de Commer rose garden	6	49.9	1583	Sp	yes	Regel	France	4	Bibliography
rosa_gallica_versicolor	Loubert rose garden	1	69.2	1581	Sp	yes	Unknown	EuropeE	4	Bibliography
rosa_gentiliana	Loubert rose garden	10	97.1	1888	Sp	no	Unknown	China	2	Bibliography
rosa_gigantea	Val de Marne rose garden	9	52.0	1889	Sp	yes	George_Watt/Henry_Collett	Asia	2	Bibliography
rosa_gigantea_x_banksiae_joanna_millar	Désert rose garden	10	98.6	2008	Unknown	no	Millar	France	Unknown	Unknown
rosa_giraldi	Loubert rose garden	12	99.2	1897	Sp	yes	Crepin	China	2	Measured
rosa_glomerata	Loubert rose garden	12	71.9	1908	Sp	yes	Unknown	China	2	Bibliography
rosa_glutinosa	Loubert rose garden	12	100.0	1821	Sp	yes	Unknown	Middle_East	Unknown	Unknown
rosa_haberiana	Loubert rose garden	15	100.0	1864	Sp	yes	Unknown	EuropeS	5	Measured
rosa_helenae	Private donations	16	100.0	1900	Sp	yes	Unknown	Asia	2	Bibliography
rosa_hemisphaerica	Arboretum des Barres	15	99.9	1516	Sp	yes	Unknown	Middle_East	4	Bibliography
rosa_henryi	Jardin botanique de la Tête d'Or	10	99.4	1907	Sp	yes	Wilson	China	2	Bibliography
rosa_heterophylla	Loubert rose garden	3	57.8	1818	Sp	yes	Unknown	EuropeW	4	Measured
rosa_hibernica	Loubert rose garden	15	99.9	1802	HSpn	no	Templeton	EuropeN	6	Bibliography
rosa_holikensis	Loubert rose garden	12	98.5	1884	HSpn	no	Unknown	EuropeW	4	Bibliography
rosa_horrida	Loubert rose garden	14	100.0	1796	Sp	yes	Unknown	EuropeE	5	Measured
rosa_huzhongyue	Flower Research Institute, China	9	42.0	Unknown	intersp	no	Unknown	China	3	Measured
rosa_hypathea	Loubert rose garden	2	75.4	1846	Unknown	no	Roseraie_de_l'Haÿ-Les-Roses	FranceParis	Unknown	Unknown
rosa_iwara	Loubert rose garden	12	99.1	1831	HMult	no	Unknown	Asia	2	Measured
rosa_iwara1	Loubert rose garden	12	99.1	1831	HMult	no	Unknown	Asia	2	Measured
rosa_jelina	Loubert rose garden	12	100.0	1894	HRg	no	Kaufmann	EuropeW	2	Bibliography
rosa_jinfenlian	Flower Research Institute, China	8	52.7	1855	intersp	no	Unknown	China	4	Measured
rosa_jinou_fanlu	Private donations	9	48.0	Unknown	T	no	Maebara	China	3	Measured
rosa_juhongchao	Flower Research Institute, China	8	45.0	Unknown	intersp	no	Unknown	China	4	Measured
rosa_junang	Flower Research Institute, China	9	35.8	Unknown	intersp	no	Maebara	China	4	Measured
rosa_jundzillii	Loubert rose garden	15	100.0	1870	Sp	yes	Unknown	EuropeE	6	Bibliography
rosa_laevigata	Loubert rose garden	10	99.2	Unknown	Sp	yes	Unknown	Asia	2	Bibliography
rosa_leucantha	Loubert rose garden	15	94.2	1557	Sp	yes	Unknown	Europe	5	Bibliography
rosa_longicuspis	Loubert rose garden	10	83.7	1915	Sp	yes	Unknown	Asia	2	Bibliography
rosa_luciae	Jardin botanique de la Tête d'Or	10	71.3	Unknown	Sp	yes	Unknown	Asia	2	Bibliography
rosa_lucidissima	Flower Research Institute, China	10	98.9	Unknown	Sp	yes	Unknown	China	Unknown	Unknown
rosa_macrophylla	Loubert rose garden	12	99.9	1818	Sp	yes	Unknown	Asia	4	Bibliography
rosa_macrophylla_glaucophylla	Loubert rose garden	12	99.7	1895	Sp	yes	Unknown	China	4	Bibliography
rosa_majalis_plena	Loubert rose garden	12	99.1	1583	Misc_OGR	yes	Unknown	EuropeN	2	Bibliography
rosa_manettii	Loubert rose garden	6	48.2	1835	N	no	Manetti	EuropeS	Unknown	Unknown
rosa_marginata	Loubert rose garden	15	100.0	1870	Sp	yes	Unknown	EuropeE	6	Bibliography
rosa_maximowicziana	Loubert rose garden	10	99.5	1880	Sp	yes	Unknown	Asia	2	Measured
rosa_melina	Loubert rose garden	12	100.0	1896	Sp	yes	Greene	America	6	Bibliography
rosa_minutifolia_alba	Loubert rose garden	12	100.0	1882	Sp	yes	Parry	America	2	Bibliography
rosa_moschata_floribunda	Loubert rose garden	12	100.0	1907	HMsk	yes	Unknown	Middle_East	2	Bibliography
rosa_moyesii	Jardin botanique de la Tête d'Or	12	99.6	1894	Sp	yes	Wilson	China	Unknown	Unknown
rosa_mudan_yue_ji	Flower Research Institute, China	8	62.9	Unknown	intersp	no	Unknown	China	4	Measured
rosa_multibracteata	Jardin botanique de la Tête d'Or	12	100.0	1908	Sp	yes	Wilson	China	4	Bibliography
rosa_multiflora	Private donations	2	84.0	Unknown	Sp	yes	Unknown	Asia	2	Bibliography
rosa_multiflora_carnea	Loubert rose garden	10	99.5	1804	Sp	yes	Unknown	China	2	Measured
rosa_multiflora_nana	Val de Marne rose garden	10	79.1	1875	Pol	yes	Unknown	Asia	2	Bibliography
rosa_multiflora_platyphylla	Val de Marne rose garden	4	63.1	1817	HMult	yes	Unknown	Asia	2	Bibliography
rosa_multiflora1	Val de Marne rose garden	2	93.1	1960	Sp	yes	Unknown	Asia	2	Bibliography
rosa_odorata	Jumaju rose garden	9	60.5	1808	T	no	Unknown	Asia	2	Bibliography
rosa_odorata_spontanea	Désert rose garden	10	89.1	Unknown	intersp	no	Unknown	Asia	Unknown	Unknown
rosa_odorata_sweet_var_erubescens	Flower Research Institute, China	10	94.3	Unknown	Misc_OGR	yes	Unknown	Asia	Unknown	Unknown
rosa_odorata_sweet_var_gigantea	Flower Research Institute, China	10	99.4	1889	Sp	yes	George_Watt/Henry_Collett	Asia	2	Measured
rosa_odorata_sweet_var_odorata	Flower Research Institute, China	10	95.3	1808	intersp	no	Unknown	China	2	Measured
rosa_odorata_sweet_var_pseudoindica	Flower Research Institute, China	10	70.6	Unknown	intersp	no	Fortune	China	2	Measured
rosa_oleifolia	Loubert rose garden	10	99.4	1900	HGal	no	Dyck	Unknown	4	Bibliography
rosa_omeiensis_chrysocarpa	Loubert rose garden	12	58.0	1890	Sp	yes	Unknown	China	Unknown	Unknown
rosa_paeonia	Loubert rose garden	4	60.1	1855	HP	no	Lacharme	FranceLyon	4	Bibliography
rosa_palustris	Jardin botanique de la Tête d'Or	12	98.3	1726	Sp	yes	Unknown	America	Unknown	Unknown
rosa_pendulina_amadis	Loubert rose garden	10	98.4	1829	Unknown	no	Laffay	FranceParis	4	Bibliography
rosa_pendulina_gracilis	Loubert rose garden	10	97.7	1830	Sp	yes	Wood	Unknown	3	Measured
rosa_pendulina_pyrenaica	Loubert rose garden	12	100.0	1815	Sp	yes	Unknown	EuropeS	4	Measured
rosa_phoenicea	Loubert rose garden	10	94.6	1885	Sp	yes	Unknown	Middle_East	2	Measured
rosa_pimpinellifolia_chlorocarpa	Val de Marne rose garden	12	97.4	1904	HSpn	no	Lévêque_de_Vilmorin	France	4	Bibliography
rosa_pimpinellifolia_double_white	Loubert rose garden	13	99.8	1815	HSpn	no	Unknown	EuropeN	4	Bibliography
rosa_polliniana	Loubert rose garden	3	67.1	1800	Misc_OGR	no	Unknown	EuropeS	4	Bibliography
rosa_polmeriana	Loubert rose garden	15	100.0	1904	Unknown	no	Pollmer	EuropeW	5	Bibliography
rosa_pomifera	Loubert rose garden	15	99.9	1770	Sp	yes	Unknown	EuropeE	Unknown	Unknown
rosa_primula	Jardin botanique de la Tête d'Or	13	50.9	1890	Sp	yes	Meyer	Asia	2	Bibliography
rosa_primulii_rosea	Loubert rose garden	12	84.5	1912	Unknown	no	Unknown	Unknown	2	Bibliography
rosa_proliferea	Val de Marne rose garden	3	78.2	1817	C	no	Unknown	Unknown	4	Bibliography
rosa_pruhoniciana	Loubert rose garden	14	100.0	1927	HMoy	no	Zeman	EuropeE	Unknown	Unknown
rosa_pteragonis	Loubert rose garden	14	100.0	1922	S	no	Hurst	EuropeN	2	Bibliography
rosa_pufuhong	Flower Research Institute, China	9	47.2	Unknown	intersp	no	Unknown	China	3	Measured
rosa_qinglian_xueshi	Flower Research Institute, China	8	60.3	Unknown	intersp	no	Unknown	China	3	Measured
rosa_rapini	Loubert rose garden	13	100.0	1859	Sp	yes	Unknown	Middle_East	4	Bibliography
rosa_reversa	Loubert rose garden	12	95.4	1820	Misc_OGR	yes	Unknown	EuropeS	3	Measured
rosa_rhodonica	Loubert rose garden	12	100.0	Unknown	Sp	yes	Unknown	Unknown	Unknown	Unknown
rosa_richardii	Loubert rose garden	3	53.7	Unknown	S	no	Unknown	Middle_East	4	Bibliography
rosa_robrosa_carmenata	Loubert rose garden	14	63.9	1923	intersp	no	Preston	America	4	Bibliography
rosa_rousseauriorum	Loubert rose garden	12	100.0	Unknown	Sp	yes	Unknown	America	2	Bibliography
rosa_roxburghi_pourpre_ancien	Loubert rose garden	3	73.8	1829	Misc_OGR	no	Unknown	Unknown	4	Measured
rosa_roxburghii_plena	Jardin botanique de la Tête d'Or	10	69.1	1824	Sp	yes	Unknown	Asia	2	Bibliography
rosa_ruanxianghong	Flower Research Institute, China	6	48.3	Unknown	intersp	no	Unknown	China	4	Measured
rosa_rubiginosa_goldblu.	Loubert rose garden	8	60.4	1954	Unknown	no	Kordes	EuropeW	5	Bibliography
rosa_rubrifolia_mechlins	Loubert rose garden	14	96.0	1788	Sp	yes	Villars	EuropeE	4	Bibliography
rosa_rubrotincta	Loubert rose garden	2	88.0	1846	HEg	no	Lee	EuropeN	5	Measured
rosa_rubrotincta1	Loubert rose garden	2	88.7	1846	HEg	no	Lee	EuropeN	5	Measured
rosa_rubus	Jardin botanique de la Tête d'Or	10	99.2	1888	Sp	yes	Unknown	China	2	Bibliography
rosa_rugosa	Arboretum des Barres	12	61.5	Unknown	Sp	yes	Unknown	Asia	2	Bibliography
rosa_rugosa_germanica	Loubert rose garden	12	99.9	1890	HRg	no	Müller	EuropeW	2	Bibliography
rosa_rugosa_scabrosa	Loubert rose garden	13	79.5	1939	HRg	no	Harkness	EuropeN	2	Bibliography
rosa_rugotida	Loubert rose garden	15	100.0	1950	HRg	no	Darthuis_Nursery	EuropeW	2	Bibliography
rosa_scabriuscula	Loubert rose garden	15	100.0	1896	Sp	yes	Unknown	France	5	Measured
rosa_scabriuscula1	Loubert rose garden	15	100.0	1896	Sp	yes	Unknown	France	5	Measured
rosa_sempervirens_prostata	Jardin botanique de la Tête d'Or	10	99.6	1824	Sp	yes	Noisette	EuropeS	2	Bibliography
rosa_sericea	Jardin botanique de la Tête d'Or	12	87.6	1822	Sp	yes	Unknown	Asia	2	Bibliography
rosa_sericea_pteracantha	Jardin botanique de la Tête d'Or	12	50.4	1890	Sp	yes	Delavay	China	2	Bibliography
rosa_setipoda	Jardin botanique de la Tête d'Or	12	99.4	1895	Sp	yes	Unknown	China	4	Bibliography
rosa_sherardii	Loubert rose garden	14	100.0	Unknown	Sp	yes	Unknown	Europe	Unknown	Unknown
rosa_simianjing	Flower Research Institute, China	7	29.2	Unknown	intersp	no	Unknown	China	3	Measured
rosa_spectabilis	Loubert rose garden	10	40.8	1833	HSem	no	Unknown	EuropeS	2	Measured
rosa_spherica	Loubert rose garden	12	97.3	1557	Sp	yes	Unknown	Europe	5	Bibliography
rosa_spithamea	Loubert rose garden	12	99.7	Unknown	Sp	yes	Unknown	America	4	Bibliography
rosa_splendens	Loubert rose garden	10	37.2	1837	Ayr	no	Unknown	Unknown	2	Bibliography
rosa_stellata	Private donations	12	99.8	1897	Sp	yes	Unknown	America	2	Measured
rosa_stylosa	Loubert rose garden	15	100.0	1838	Sp	yes	Unknown	EuropeE	5	Measured
rosa_subgallicoïdes	Loubert rose garden	10	99.3	1900	Sp	yes	Unknown	France	2	Measured
rosa_sulphurea	Loubert rose garden	13	100.0	1516	Sp	yes	Unknown	Middle_East	4	Bibliography
rosa_tenuicarpa	Val de Marne rose garden	10	60.1	1877	HSem	no	Lévêque_de_Vilmorin	France	2	Bibliography
rosa_tomentosa	Loubert rose garden	15	100.0	1808	Sp	yes	Unknown	EuropeE	5	Bibliography
rosa_uncinella	Loubert rose garden	15	100.0	1811	Sp	yes	Unknown	Middle_East	5	Measured
rosa_villosa_recondita	Loubert rose garden	3	52.7	1771	Sp	no	Unknown	EuropeE	4	Measured
rosa_violacea	Loubert rose garden	2	93.5	1795	HGal	no	Unknown	EuropeW	4	Bibliography
rosa_virginiana	Jardin botanique de la Tête d'Or	12	99.9	1807	Sp	yes	Unknown	America	4	Bibliography

rosa_wichurana	Loubert rose garden	10	98.2	1891	Sp	yes	Unknown	Asia	2	Measured
rosa_willmottiae	Arboretum des Barres	12	89.1	1904	Sp	yes	Unknown	China	2	Bibliography
rosa_woodsi_fendleri	Loubert rose garden	12	99.9	1888	Sp	yes	Unknown	America	5	Measured
rosa_x_borboniana	Jardin botanique de la Tête d'Or	9	29.7	1819	B	no	Unknown	Africa	4	Bibliography
rosa_x_calocarpa	Jardin botanique de la Tête d'Or	7	35.4	1891	HRg	no	Bruant	FranceAngers	2	Bibliography
rosa_x_centifolia	Jardin botanique de la Tête d'Or	1	60.8	1450	C	no	Unknown	EuropeW	4	Bibliography
rosa_x_centifolia_pomponia	La Cour de Commer rose garden	2	87.2	1659	C	no	Unknown	France	4	Bibliography
rosa_x_damascena	Jardin botanique de la Tête d'Or	4	72.3	1200	D	no	Unknown	Middle_East	4	Bibliography
rosa_x_iwara	Val de Marne rose garden	12	83.7	1830	HMult	no	Unknown	Asia	2	Bibliography
rosa_x_malyi	Loubert rose garden	12	99.9	1869	S	no	Maly	EuropeW	Unknown	Unknown
rosa_x_noisettiana	Val de Marne rose garden	9	61.5	1814	N	no	Noisette	America	2	Measured
rosa_x_noisettiana_manettii	Val de Marne rose garden	9	53.8	1835	N	no	Manetti	EuropeS	Unknown	Unknown
rosa_X_odorata_ochroleuca	Loubert rose garden	10	99.5	1824	T	no	Unknown	China	2	Bibliography
rosa_x_polliniana	Jardin botanique de la Tête d'Or	3	75.7	1800	Misc_OGR	no	Unknown	EuropeS	4	Bibliography
rosa_x_wichurana	INRA Angers	11	66.8	1891	Sp	yes	Unknown	Asia	2	Measured
rosa_xanthina	Jardin botanique de la Tête d'Or	13	100.0	1906	Sp	yes	Meyer	Asia	2	Bibliography
rosa_xanthina_duplex	Loubert rose garden	12	57.4	1906	Sp	yes	Meyer	Asia	2	Bibliography
rosa_xanthina_spontanea	Loubert rose garden	12	70.7	1907	S	yes	Meyer	Asia	Unknown	Unknown
rosa_yijifen	Flower Research Institute, China	9	51.2	Unknown	intersp	no	Unknown	China	3	Measured
rosa_yingri_hehua	Flower Research Institute, China	8	92.0	Unknown	intersp	no	Unknown	China	4	Measured
rosa_yiping_zhuyi	Flower Research Institute, China	6	65.4	Unknown	intersp	no	Unknown	China	4	Measured
rosa_yunzheng_xiawei	Flower Research Institute, China	8	92.7	Unknown	intersp	no	Unknown	China	4	Measured
rosa_yushzuang	Flower Research Institute, China	8	90.8	Unknown	intersp	no	Unknown	China	3	Measured
rosa_zihongxiang	Flower Research Institute, China	8	87.5	Unknown	intersp	no	Unknown	China	4	Measured
rosabelle	Val de Marne rose garden	9	54.6	1899	T	no	Bruant	FranceAngers	2	Measured
rose_a_parfum_de_l_hay	Jardin botanique de la Tête d'Or	10	87.1	1901	HRg	no	Gravereaux	FranceParis	3	Bibliography
rose_d_herbey_s	Grande roseraie de Lyon	9	74.3	1902	T	no	Schwartz	FranceLyon	2	Bibliography
rose_de_france	Val de Marne rose garden	6	48.3	1894	HP	no	Verdier	FranceParis	4	Bibliography
rose_de_meaux_blanc	Val de Marne rose garden	2	80.8	1799	C	no	Unknown	Unknown	4	Bibliography
rose_de_provins	Val de Marne rose garden	2	94.5	1160	Sp	no	Unknown	Europe	4	Bibliography
rose_edouard	Loubert rose garden	6	69.5	1820	B	no	Unknown	Africa	4	Measured
rose_foucheaux	Val de Marne rose garden	2	82.7	1846	HGal	no	Unknown	Unknown	4	Bibliography
rose_nabonnand	Désert rose garden	9	46.8	1882	T	no	Nabonnand	FranceSE	2	Bibliography
rosier_d_amour	Val de Marne rose garden	4	71.2	1880	HGal	no	Crantz	Unknown	4	Bibliography
rosier_des_alpes	Loubert rose garden	12	100.0	1770	Sp	yes	Unknown	Unknown	3	Measured
rosier_eveque	Val de Marne rose garden	4	55.0	1797	HGal	no	Unknown	Unknown	4	Bibliography
rosita_mauri	Val de Marne rose garden	8	63.3	1913	HT	no	Ketten	EuropeW	4	Bibliography
rote_hermosa	Loubert rose garden	6	32.5	1899	Ch	no	Geissler	EuropeW	4	Measured
rotkapchen	Jumaju rose garden	9	56.3	1887	Pol	no	Geschwind	EuropeW	Unknown	Unknown
rotrou	La Cour de Commer rose garden	2	91.3	1848	M	no	Vibert	FranceAngers	4	Bibliography
rouge_adam	La Beaujoire rose garden	8	76.7	1996	HT	no	Adam	FranceNW	4	Bibliography
roulettii	Jumaju rose garden	9	72.1	1801	Ch	no	Unknown	China	Unknown	Unknown
roxelane	Jumaju rose garden	3	49.0	1829	HCh	no	Noisette	FranceParis	3	Measured
royat_mondain	Val de Marne rose garden	5	90.3	1902	HP	no	Veysset	FranceNE	4	Bibliography
ruby_queen	Val de Marne rose garden	8	57.8	1901	HWich	no	Manda	America	2	Bibliography
russell_s_cottage	Val de Marne rose garden	3	57.5	1826	HMult	no	Vineux	Unknown	2	Bibliography
sachsengruss	Jumaju rose garden	8	54.6	1912	HP	no	Neubert	EuropeW	4	Bibliography
safrano	Grande roseraie de Lyon	6	61.0	1839	T	no	Beauregard	FranceAngers	2	Bibliography
salet	Loubert rose garden	5	87.1	1854	M	no	Lacharme	FranceLyon	4	Measured
sally_holmes	La Beaujoire rose garden	8	48.4	1976	S	no	Holmes	EuropeN	2	Bibliography
sanchette	La Cour de Commer rose garden	2	96.0	1837	HGal	no	Vibert	FranceParis	4	Bibliography
sanders_white	Jardin botanique de la Tête d'Or	10	99.1	1912	HWich	no	Sanders	EuropeN	2	Bibliography
Sanguinea	Jumaju rose garden	10	61.2	1837	Ch	no	Vibert	China	2	Bibliography
sans_petales	La Cour de Commer rose garden	4	68.4	1790	C	no	Poilpré	FranceAngers	4	Bibliography
sapho	Jumaju rose garden	16	100.0	1843	P	no	Vibert	FranceAngers	Unknown	Unknown
schneelicht	Loubert rose garden	12	99.9	1896	HRg	no	Geschwind	EuropeW	2	Measured
schneewittchen	Val de Marne rose garden	10	95.9	1901	Pol	no	Lambert	EuropeW	2	Bibliography
seguier	Val de Marne rose garden	4	60.4	1853	HGal	no	Robert	FranceAngers	4	Bibliography
shailer_s_white_moss	La Cour de Commer rose garden	4	72.3	1788	M	no	Shailer	EuropeN	4	Bibliography
shubin34	Flower Research Institute, China	9	64.7	Unknown	Unknown	no	Unknown	China	Unknown	Unknown
sidonie	Loubert rose garden	4	70.3	1845	HP	no	Dorisy	FranceNE	4	Measured
silver_queen	Loubert rose garden	4	41.8	1886	HP	no	Paul	EuropeN	3	Measured
single_pink_china	Désert rose garden	9	78.4	Unknown	Ch	no	Unknown	China	2	Bibliography
sir_garnet_wolseley	Val de Marne rose garden	6	30.6	1875	HP	no	Cranston	EuropeN	4	Bibliography
sir_joseph_paxton	Loubert rose garden	6	41.0	1851	B	no	Laffay	FranceParis	4	Measured
slater_s_crimson_china	Flower Research Institute, China	9	69.2	1759	Ch	no	Unknown	China	3	Measured
sœur_marthe	Loubert rose garden	2	80.1	1848	M	no	Vibert	FranceAngers	4	Measured
solfatare	Loubert rose garden	9	70.5	1843	N	no	Boyau	FranceAngers	2	Measured
soupert_et_notting	La Cour de Commer rose garden	8	88.6	1874	M	no	Pernet	FranceLyon	4	Bibliography
source_d_or	Val de Marne rose garden	9	56.3	1912	LCl	no	Turbat	FranceOrleans	2	Bibliography
south_orange_perfection	Val de Marne rose garden	9	59.6	1898	HWich	no	Horvath	America	2	Bibliography
souv_d_auguste_riviere	Val de Marne rose garden	5	91.5	1877	HP	no	Verdier	FranceParis	4	Bibliography
souv_de_catherine_guillot	Désert rose garden	9	41.5	1895	T	no	Guillot	FranceLyon	Unknown	Unknown
souv_de_francois_gaulain	Désert rose garden	9	69.3	1889	T	no	Guillot	FranceLyon	2	Bibliography
souv_de_germain_de_saint_pierre	Jardin botanique de la Tête d'Or	9	46.5	1882	T	no	Nabonnand	FranceSE	Unknown	Unknown
souv_de_gilbert_nabonnand	SCRADH	9	75.4	1920	T	no	Nabonnand	FranceSE	4	Bibliography
souv_de_henry_leveque_de_vilmorin	Val de Marne rose garden	8	48.6	1889	HP	no	Lévêque	FranceParis	4	Bibliography
souv_de_j_b_guillot	Grande roseraie de Lyon	9	55.2	1897	T	no	Guillot	FranceLyon	Unknown	Unknown
souv_de_la_malmaison	Loubert rose garden	8	86.8	1843	B	no	Béluze	FranceLyon	3	Bibliography
souv_de_la_malmaison_cl	Loubert rose garden	8	87.4	1893	B	no	Bennett	EuropeN	4	Bibliography
souv_de_la_reine_d_angleterre	Val de Marne rose garden	5	72.2	1855	HP	no	Cochet	FranceParis	4	Bibliography
souv_de_mac_kinley	Jumaju rose garden	5	58.2	1893	HT	no	Schwartz	FranceLyon	Unknown	Unknown
souv_de_mme_auguste_charles	Loubert rose garden	7	41.2	1866	B	no	Moreau_&_Robert	FranceAngers	4	Bibliography
souv_de_mme_ladvocat	Val de Marne rose garden	6	60.0	1899	N	no	Veysset	FranceNE	4	Measured
souv_de_mme_sablayrolles	Désert rose garden	9	80.6	1890	T	no	Bonnaire	FranceLyon	2	Bibliography
souv_de_pierre_sionville	Private donations	8	76.7	1906	HP	no	Boutigny	FranceNW	4	Bibliography
souv_de_pierre_vibert	La Cour de Commer rose garden	2	93.8	1867	M	no	Moreau_&_Robert	FranceAngers	4	Bibliography
souv_de_victor_landeau	Val de Marne rose garden	8	85.3	1890	B	no	Moreau_&_Robert	FranceAngers	4	Bibliography
souv_de_yeddo	Loubert rose garden	10	92.3	1874	HRg	no	Morlet	FranceNE	2	Bibliography
souv_du_comte_de_cavour	Val de Marne rose garden	6	46.3	1861	HP	no	Margottin	FranceParis	4	Bibliography
souv_du_docteur_jamain	Val de Marne rose garden	6	37.1	1865	HP	no	Lacharme	FranceLyon	4	Bibliography
souv_du_rosieriste_rambaux	Grande roseraie de Lyon	9	43.3	1884	T	no	Rambaux	FranceLyon	2	Bibliography
souvenir_alphonse_lavallee	Jumaju rose garden	6	37.4	1884	HP	no	Turc	France	Unknown	Unknown
souvenir_de_brod	Loubert rose garden	5	91.7	1886	HSet	no	Geschwind	EuropeW	4	Measured
souvenir_de_lucie	Loubert rose garden	5	51.2	1893	N	no	Schwartz	FranceLyon	4	Measured
souvenir_de_mme_berthier	Loubert rose garden	6	68.9	1881	HP	no	Berthier	FranceLyon	4	Measured
souvenir_de_philemon_cochet	Loubert rose garden	12	78.0	1899	HRg	no	Cochet	FranceParis	2	Measured
souvenir_du_president_carnot	Loubert rose garden	8	97.3	1894	HT	no	Pernet-Ducher	FranceLyon	3	Measured
souvenir_du_president_carnot	Loubert rose garden	8	95.0	1894	HT	no	Pernet-Ducher	FranceLyon	3	Bibliography
spencer	Loubert rose garden	5	57.0	1902	HP	no	Paul	EuropeN	3	Measured
staffa	Loubert rose garden	13	99.3	1832	HSpn	no	Unknown	EuropeN	4	Bibliography
stanwell_perpetual	Loubert rose garden	12	100.0	1835	HSpn	no	Lee	EuropeN	4	Bibliography
sterckmans	Val de Marne rose garden	2	85.6	1842	HGal	no	Unknown	EuropeW	4	Bibliography
sunset	Désert rose garden	8	84.2	1883	T	no	Henderson	America	Unknown	Unknown
surpasse_tout	Val de Marne rose garden	2	52.1	1811	HGal	no	Unknown	EuropeW	4	Bibliography
tausendschon	Val de Marne rose garden	10	83.0	1906	HMult	no	Kiese	EuropeW	2	Bibliography
tendresse	Val de Marne rose garden	8	83.0	1980	HT	no	Delbard	FranceParis	4	Bibliography
thalia	Val de Marne rose garden	10	85.3	1895	HMult	no	Schmitt	FranceLyon	2	Bibliography
the_fairy	INRA Angers	10	33.7	1932	Pol	no	Bentall	EuropeN	2	Measured
the_garland	Loubert rose garden	10	56.1	1835	HMult	no	Wells	EuropeN	2	Measured
the_new_century	Loubert rose garden	12	100.0	1900	HRg	no	Van_Fleet	America	Unknown	Unknown
the_wallflower	Val de Marne rose garden	8	31.4	1901	HMult	no	Paul	EuropeN	2	Bibliography
theano	Loubert rose garden	10	61.3	1895	HWich	no	Geschwind	EuropeW	Unknown	Unknown
thoresbyana	Jardin botanique de la Tête d'Or	10	62.0	1840	Ayr	no	Bennett	EuropeN	2	Bibliography
tian_nu_guang	Désert rose garden	8	72.9	2008	T	no	Maebara	Asia	2	Bibliography
tiergarten	Val de Marne rose garden	10	87.3	1904	HMult	no	Lambert	EuropeW	2	Bibliography
tom_wood	Loubert rose garden	6	63.2	1896	HP	no	Dickson	EuropeN	4	Bibliography
tour_de_malakoff	La Cour de Commer rose garden	15	52.0	1857	C	no	Soupert_&_Notting	EuropeW	4	Bibliography
toussaint_l_ouverture	Loubert rose garden	2	84.8	1849	B	no	Miellez	FranceNW	4	Measured

tricolore	Jardin botanique de la Tête d’Or	4	69.9	1863	HMult	no	Moreau_&_Robert	FranceAngers	2	Bibliography
trier	Val de Marne rose garden	10	91.1	1904	HMult	no	Lambert	EuropeW	2	Bibliography
triomphe_de_caen	Loubert rose garden	6	71.2	1861	HP	no	Oger	FranceNW	4	Bibliography
triomphe_de_l_exposition	Loubert rose garden	5	92.4	1855	HP	no	Margottin	FranceParis	4	Bibliography
triomphe_de_la_duchere	Jardin botanique de la Tête d’Or	9	44.0	1846	B	no	Béluze	FranceLyon	Unknown	Unknown
triomphe_de_pernet_pere	Val de Marne rose garden	8	45.9	1890	HT	no	Pernet	FranceLyon	4	Bibliography
triomphe_des_noisettes	Loubert rose garden	8	87.8	1887	N	no	Pernet	FranceLyon	4	Measured
turenne	Val de Marne rose garden	2	72.1	1846	HGal	no	Vibert	FranceAngers	4	Bibliography
tuscany	La Cour de Commer rose garden	2	93.9	1820	HGal	no	Unknown	Unknown	4	Bibliography
tuscany_superb	Val de Marne rose garden	2	88.5	1837	HGal	no	Rivers	EuropeN	4	Bibliography
ulrich_brunner_fils	Grande roseraie de Lyon	6	44.3	1881	HP	no	Levet	FranceLyon	4	Bibliography
una	Loubert rose garden	6	52.4	1898	S	no	Paul	EuropeN	4	Measured
unique_rose	Val de Marne rose garden	2	84.9	1810	C	no	Cels	FranceParis	4	Bibliography
van_artevelde	La Cour de Commer rose garden	2	89.9	1847	HGal	no	Parmentier	EuropeW	4	Bibliography
veilchenblau	Val de Marne rose garden	10	37.3	1909	HMult	no	Schmidt	EuropeW	2	Bibliography
velours_pourpre	Loubert rose garden	15	100.0	1866	HGal	no	Unknown	EuropeW	4	Bibliography
venus	La Cour de Commer rose garden	4	76.5	1845	HGal	no	Welter	EuropeW	4	Bibliography
vesuvia	La Beaujoire rose garden	9	64.1	2001	S	no	Noak	EuropeW	Unknown	Unknown
vick_s_caprice	Val de Marne rose garden	5	70.1	1889	HP	no	Vick	America	4	Bibliography
vicomtesse_d_avesne	La Beaujoire rose garden	7	32.1	1847	N	no	Roeser	FranceParis	3	Measured
victor_parmentier	La Cour de Commer rose garden	2	87.6	1847	HGal	no	Parmentier	EuropeW	4	Bibliography
victor_verdier	Grande roseraie de Lyon	5	65.4	1859	HP	no	Lacharme	FranceLyon	4	Bibliography
vierge_de_clery	La Cour de Commer rose garden	2	63.2	1888	C	no	Baron-Veillard	FranceOrleans	4	Bibliography
ville_de_bruxelle	Loubert rose garden	3	56.6	1836	D	no	Vibert	FranceParis	3	Measured
ville_de_saint_denis	Loubert rose garden	5	75.4	1853	HP	no	Thomas	FranceNW	4	Bibliography
ville_de_toulouse	Val de Marne rose garden	2	82.4	1876	HGal	no	Brassac	FranceSW	4	Bibliography
vinoca	Val de Marne rose garden	8	69.1	1906	HT	no	Fonseca	America	4	Bibliography
violacee	La Cour de Commer rose garden	2	90.7	1876	M	no	Soupert_&_Notting	EuropeW	4	Bibliography
virago	Loubert rose garden	5	56.9	1887	HSet	no	Geschwind	EuropeW	4	Measured
vivid	Loubert rose garden	4	52.8	1853	B	no	Paul	EuropeN	4	Measured
waltham_climber_2	Val de Marne rose garden	8	39.4	1885	HT	no	Paul	EuropeN	4	Bibliography
wasily_chludoff	SCRADH	9	38.7	1896	N	no	Nabonnand	FranceSE	2	Bibliography
white_dorothy_perkins	Val de Marne rose garden	11	39.4	1908	HWich	no	Cant	EuropeN	2	Bibliography
white_scotch	Val de Marne rose garden	13	100.0	1906	HSpn	no	Unknown	EuropeN	4	Bibliography
william_allen_richardson	Val de Marne rose garden	9	82.9	1878	N	no	Ducher	FranceLyon	2	Measured
william_c._egan	Loubert rose garden	6	61.5	1900	HWich	no	Dawson	America	3	Measured
william_lobb	La Cour de Commer rose garden	16	100.0	1855	M	no	Laffay	FranceParis	4	Bibliography
williams_double_yellow	Loubert rose garden	13	100.0	1838	HFt	no	Williams	EuropeN	3	Bibliography
wodan	Loubert rose garden	5	84.9	1890	HMult	no	Geschwind	EuropeW	4	Measured
yuki_s_dream	Flower Research Institute, China	7	35.7	Unknown	Unknown	no	Unknown	China	Unknown	Unknown
yvonne_rabier	Val de Marne rose garden	9	40.6	1910	Pol	no	Turbat	FranceOrleans	2	Bibliography
zaire	La Cour de Commer rose garden	2	91.3	1849	M	no	Vibert	FranceAngers	4	Bibliography
zephirine_drouhin	Loubert rose garden	6	52.9	1868	B	no	Bizot	France	3	Bibliography
zoe	Loubert rose garden	2	76.3	1861	M	no	Forest	France	4	Measured

Legend

Classification

A: Alba
Ayr: Ayrshire
B: Bourbon
C: Centifolia
Ch: China
D: Damask
F: Floribunda
Gr: Grandiflora
HCh: Hybrid
HEg: Hybrid Eglanteria
HFt: Hybrid Foetida
HG: Hybrid Gigantea
HGal: Hybrid Gallica
HMoy: Hybrid Moyesii
HMsk: Hybrid Musk
HMult: Hybrid Multiflora
HP: Hybrid Perpetual
HRg: Hybrid Rugosa
HSem: Hybrid Sempervirens
HSet: Hybrid Setigera
HSpn: Hybrid Spinosissima
HT: Hybrid Tea
HWich: Hybrid Wichurana
intersp: interspercies
LCl: Large-Flowered Climber
MiscOGR: Miscellaneous OGR
M: Moss
N: Noisette
P: Portland
Pol: Polyantha
S: Shrub
Sp: Species
T: Tea

Origin

EuropeE: Europe East
EuropeN: Europe North
EuropeS: Europe South
EuropeW: Europe West
FranceNE: France North East
FranceNW: France North West
FranceSE: France South East
FranceSW: France South West
